# Meta‐replication, sampling bias, and multi‐scale model selection: A case study on snow leopard (*Panthera uncia*) in western China

**DOI:** 10.1002/ece3.6492

**Published:** 2020-07-06

**Authors:** Luciano Atzeni, Samuel A. Cushman, Defeng Bai, Jun Wang, Pengju Chen, Kun Shi, Philip Riordan

**Affiliations:** ^1^ Wildlife Institute School of Ecology and Nature Conservation Beijing Forestry University Beijing China; ^2^ US Forest Service Rocky Mountain Research Station Flagstaff AZ USA; ^3^ Faculty of Science and Engineering Manchester Metropolitan University Manchester UK; ^4^ Eco‐Bridge Continental Beijing China; ^5^ Marwell Wildlife Winchester UK

**Keywords:** MaxEnt, meta‐replication, multi‐scale, *Panthera uncia*, sampling bias, scale selection, snow leopard, species distribution model

## Abstract

Replicated multiple scale species distribution models (SDMs) have become increasingly important to identify the correct variables determining species distribution and their influences on ecological responses. This study explores multi‐scale habitat relationships of the snow leopard (*Panthera uncia*) in two study areas on the Qinghai–Tibetan Plateau of western China. Our primary objectives were to evaluate the degree to which snow leopard habitat relationships, expressed by predictors, scales of response, and magnitude of effects, were consistent across study areas or locally landcape‐specific. We coupled univariate scale optimization and the maximum entropy algorithm to produce multivariate SDMs, inferring the relative suitability for the species by ensembling top performing models. We optimized the SDMs based on average omission rate across the top models and ensembles’ overlap with a simulated reference model. Comparison of SDMs in the two study areas highlighted landscape‐specific responses to limiting factors. These were dependent on the effects of the hydrological network, anthropogenic features, topographic complexity, and the heterogeneity of the landcover patch mosaic. Overall, even accounting for specific local differences, we found general landscape attributes associated with snow leopard ecological requirements, consisting of a positive association with uplands and ridges, aggregated low‐contrast landscapes, and large extents of grassy and herbaceous vegetation. As a means to evaluate the performance of two bias correction methods, we explored their effects on three datasets showing a range of bias intensities. The performance of corrections depends on the bias intensity; however, density kernels offered a reliable correction strategy under all circumstances. This study reveals the multi‐scale response of snow leopards to environmental attributes and confirms the role of meta‐replicated study designs for the identification of spatially varying limiting factors. Furthermore, this study makes important contributions to the ongoing discussion about the best approaches for sampling bias correction.

## Graphical abstract

Meta‐replication in distribution modeling represents a powerful approach to identify either the locally specific or broad ecological responses of a focal species. We compared two landscapes of western China, which differ in limiting factors, testing the multi‐scale response of snow leopard (*Panthera uncia*) to several environmental predictors. We suggest a new method for model selection, and explore the effect of two correction types on datasets showing different bias intensities.

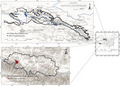

## 1 INTRODUCTION

Species distribution models (SDMs) are empirical quantitative methods that relate species occurrence data to environmental predictors and identify the suite of environmental conditions in which a species can be maintained (Guisan, Thuiller, & Zimmermann, 2017; Guisan & Zimmermann, 2000; McGarigal, Wan, Zeller, Timm, & Cushman, 2016). Predictions from SDMs are centrally important to both theoretical and applied ecology and are the main tool used to predict potential occurrence of a species in the absence of systematic surveys or over wide areas (Bai, Chen, & Atzeni, 2018; Watts, McCarthy, & Namgail, 2019), to evaluate degrees of niche overlap (Aryal et al., 2016; Hearn et al., 2018; Khosravi, Hemami, & Cushman, 2019; Vergara, Cushman, Urra, & Ruiz‐González, 2015), or predict distributional shifts under climatic changes (Aryal et al., 2016; Li et al., 2016; Shirk et al., 2018).

Much emphasis has been placed on presence‐only (PO) distribution models and models using pseudo‐absence data (Barbet‐Massin, Jiguet, Albert, & Thuiller, 2012; Phillips, Anderson, & Schapire, 2006). Such models are often used where robust information about species absence is lacking due to sampling and financial limitations (Pearson, Raxworthy, Nakamura, & Peterson, 2007; Phillips et al., 2006). However, PO data can be geographically biased, often reflecting differences in sampling intensity across areas (Bystriakova, Peregrym, Erkens, Bezsmertna, & Schneider, 2012; Kramer‐Schadt et al., 2013; Phillips et al., 2009; Syfert, Smith, & Coomes, 2013; Veloz, 2009). For this reason, much attention has focused on assessing methods that account for and correct uneven occurrence distribution, to generate reliable predictions from a nonsystematic sampling (Acevedo, Jiménez‐Valverde, Lobo, & Real, 2012; Boria, Olson, Goodman, & Anderson, 2014; Fourcade, Engler, Rödder, & Secondi, 2014; Hijmans, 2012; Kramer‐Schadt et al., 2013; Phillips et al., 2009; Syfert et al., 2013; Varela, Anderson, García‐Valdés, & Fernández‐González, 2014; Veloz, 2009; Vergara et al., 2015). Since each study will differ in bias intensity and spatial configuration of records, Fourcade et al. (2014) advocated the assessment of multiple correction strategies in improving model accuracy and prediction.

One method of species distribution modeling with PO data employs the maximum entropy machine learning algorithm, implemented in the software MaxEnt (Phillips et al., 2006; Phillips & Dudík, 2008). This approach uses presence against background data to provide estimates of relative suitability (Elith et al., 2011; Guillera‐Arroita, Ridout, & Morgan, 2014; Merow, Smith, & Silander, 2013). MaxEnt has gained popularity due to its intuitive use and due to its high predictive performance relative to several other algorithms (Elith et al., 2006; Phillips & Dudík, 2008) and has been successfully applied to modeling the habitat of cryptic species (Aryal et al., 2016; Bai et al., 2018; Erfanian, Mirkarimi, Mahini, & Rezaei, 2013; Khosravi et al., 2019; Kittle, Watson, Cushman, & Macdonald, 2018; McCarthy, Wibisono, McCarthy, Fuller, & Andayani, 2015; Watts et al., 2019; Wilting et al., 2010).

Typically, SDMs are developed using all the predictors measured at a fixed scale. However, single‐scale modeling risks incorrectly describing a species’ responses to features of the environment (Mateo‐Sánchez, Cushman, & Saura, 2013; McGarigal et al., 2016; Timm, McGarigal, Cushman, & Ganey, 2016). Each species will experience their environment at a range of different scales (Levin, 1992) in relation to life history traits and ecological requirements (Addicott et al., 1987; Johnson, 1980; Wiens, 1989). The correct identification of scales at which animals perceive and respond to landscape features should therefore be an important focus of ecological and distribution studies (Levin, 1992; McGarigal et al., 2016; Wiens, 1989).

Failure to optimize observational scales in studies of pattern‐process relationships can result in predictive errors and incorrect inferences (McGarigal et al., 2016; Thompson & McGarigal, 2002; Wasserman, Cushman, Wallin, & Hayden, 2012; Wiens, 1989). In many cases, correctly identifying the multi‐scale nature of such relationships provides a more accurate description of the ecological processes of interest (Bellamy, Scott, & Altringham, 2013; Khosravi et al., 2019; Mateo‐Sánchez et al., 2013; Thompson & McGarigal, 2002; Timm et al., 2016; Vergara et al., 2015; Wan et al., 2017; Wasserman, Cushman, Wallin, et al., 2012).

Previous research has shown that multi‐scale optimization is critical in producing reliable predictions of carnivore habitat (e.g., Mateo‐Sánchez et al., 2013; Vergara et al., 2015; Wasserman, Cushman, Wallin, et al., 2012) and felids in particular (e.g., Ashrafzadeh et al., 2020; Elliot, Cushman, Macdonald, & Loveridge, 2014; Hearn et al., 2018; Krishnamurthy et al., 2016; Macdonald et al., 2018, 2019). For example, Ashrafzadeh et al. (2020) employed a multi‐scale, multi‐species approach to model habitat suitability and connectivity for six felids across Iran, finding that each species' habitat use was influenced in a scale‐dependent manner by different sets of environmental variables. Similarly, Hearn et al. (2018) modeled multi‐scale habitat suitability of four felids across Sabah, Borneo, and found species‐specific differences in the scale of habitat associations, with most species associated with broad scales of environmental variation. Therefore, multi‐scale SDMs, which describe how the contribution of each variable varies across scales, produce more accurate, organism‐centered, distribution models (McGarigal et al., 2016).

However, there is no methodology to define, a priori, the scales at which a given predictor exerts the strongest influence on species (McGarigal et al., 2016; Shirk, Wasserman, Cushman, & Raphael, 2012). In this context, it is important to apply scale optimization approaches to identify the prevailing scale of statistical response (Bellamy et al., 2013; Khosravi et al., 2019; Mateo‐Sánchez et al., 2013; Shirk, Raphael, & Cushman, 2014; Shirk et al., 2012; Timm et al., 2016; Vergara et al., 2015; Wan et al., 2017; Wasserman, Cushman, Wallin, et al., 2012).

With this study, we assess the performance of multi‐scale models versus single‐scale models, in terms of accuracy and predictive ability, with data from a wide‐ranging top predator, the snow leopard (*Panthera uncia*). The snow leopard is a species of conservation concern, listed as Vulnerable by IUCN (McCarthy, Mallon, Jackson, Zahler, & McCarthy, 2017), and is regarded as a flagship species for the mountainous habitats of Central Asia. The global population is estimated to be 2,710–3,386 mature individuals (McCarthy et al., 2017), though there is substantial uncertainty. Estimates of local abundance and studies on distribution remain scarce (Mallon & Jackson, 2017; McCarthy, Mallon, Sanderson, Zahler, & Fisher, 2016; Robinson & Weckworth, 2016). To understand population distribution, status, and trend, it is important to identify areas whose characteristics are most favorable to snow leopard presence and persistence, which might result in strengthened survey efforts and conservation measures.

We investigated snow leopard habitat relationships in two landscapes of Western China: the Qilian Mountains (Gansu and Qinghai Provinces) and in the Himalayas of the Tibetan Autonomous Region. Of the few published studies from these areas, most have focused on site occupancy, density estimation, and human perception toward snow leopards (Alexander, Chen, et al., 2015; Alexander, Gopalaswamy, Shi, Hughes, & Riordan, 2016; Alexander, Gopalaswamy, Shi, & Riordan, 2015; Alexander, Shi, Tallents, & Riordan, 2016; Alexander, Zhang, Shi, & Riordan, 2016; Chen et al., 2016, 2017). Bai et al. (2018) produced the first habitat suitability model for snow leopard using the MaxEnt algorithm in the Qomolangma National Nature Reserve, in the Chinese Himalayas.

Comparing species–habitat relationships across meta‐replicated study areas can provide more reliable and generalizable information about the factors and scales that drive species occurrence and distribution patterns (e.g., Cushman et al., 2011; Shirk et al., 2012; Short Bull et al., 2011; Wan et al., 2017), and the effects of landscape patterns on ecological processes generally (e.g., McGarigal & Cushman, 2002).

Previous distribution models on snow leopards covered the areas of Ladakh, India (Watts et al., 2019), Southern Russia (Kalashnikova, Karnaukhov, & Dubinin, 2019), the northwestern part of the range in central Asia (Holt, Nevin, Smith, & Convery, 2018) and the Nepalese Himalayas (Aryal et al., 2016). Earlier, Li (2012) modeled the range‐wide distribution of this species. Further SDMs described range expansion and contraction under past (Li et al., 2016) and future (Aryal et al., 2016; Li et al., 2016) climate change scenarios. Recently, Li et al. (2020), modeled range‐wide snow leopard habitat as a means to infer a resistance map to guide management recommendations. None of these studies, however, explicitly considered scale issues (sensu McGarigal et al., 2016), nor adopted a formal meta‐replication framework (sensu Shirk et al., 2012).

With this study, we focus on the multi‐scale habitat relationships of snow leopards in two study areas characterized by different topography, land cover, and climatic attributes to: (a) identify the landscape‐specific predictors of snow leopard relative habitat suitability; (b) assess the influence of scale on snow leopard habitat relationships and identify the scales at which their effect is most pronounced; (c) assess the performance of multi‐scale models versus single‐scale models, comparing relative accuracy and predictive ability; (d) provide a framework for model selection and correction of biased occurrence records; (e) create predictions from ensembles of competing distribution models, built with different variables, to probabilistically infer relative suitability. As a further objective (provided as Appendix material), we (f) assess the efficacy of two correction methods under different bias intensities. To our knowledge, this is the first SDM on snow leopard adopting a multi‐scale and meta‐replicated approach.

## 2 METHODS

### 2.1 Study areas

**FIGURE 1:**
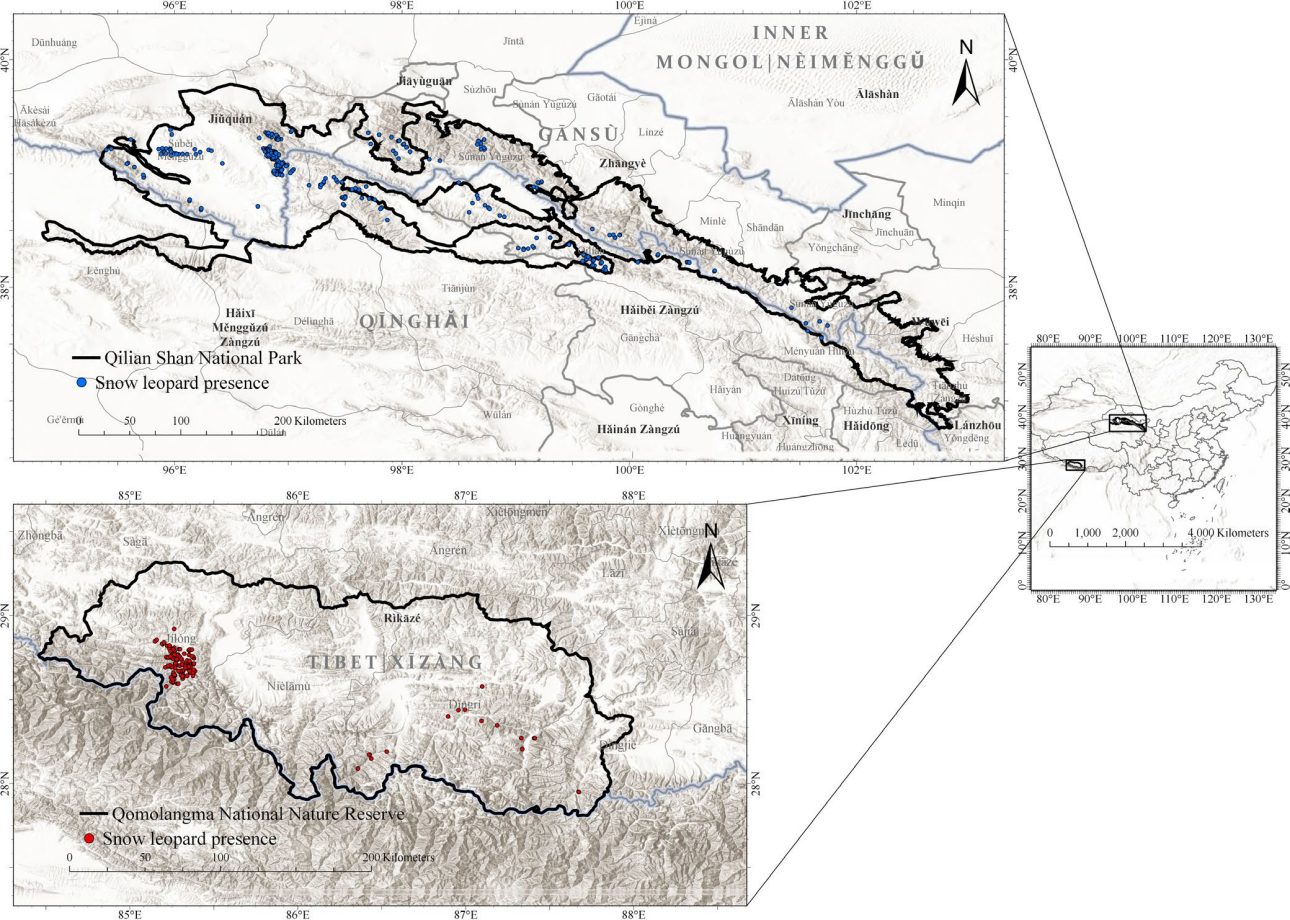
Map of the two study areas. Top = Qilianshan National Park, Gansu and Qinghai, China. Bottom = Qomolangma National Nature Reserve, Xizang (Tibet) Autonomous Region, China

The first study area lies within the Qomolangma National Nature Reserve (QMLNR, N27°48′ – 29°19′, E84°27′ – 88°23′, in the Xizang (西藏, Tibet) Autonomous Region of China. In its 33,814 km^2^, the reserve encompasses semi‐humid mountain forest in the southern part and semi‐arid shrub in the northern part (Bai et al., 2018). From 2014 to 2017 we surveyed Jilong County (吉隆县), Dingjie County (定结县), and Dingri County (定日县) (Bai et al., 2018; Chen et al., 2017) for snow leopard occurrence. The second study area is located in Gansu (甘肃) and Qinghai (青海) Provinces. From 2014 to 2017 we surveyed three mountain ranges in Yanchiwan (盐池湾) National Nature Reserve, Gansu (YNR, N38°33′ – 39°10′, E95°19′ – 97°13′). YNR is located in Subei Mongolian Autonomous County (肃北蒙古族自治县), and is inhabited mainly by semi‐nomadic herders of Mongolian ethnicity. The protected area is about 13,600 km^2^ with an average elevation of 4,800 m. In the same Province, we surveyed parts of Qilian Shan (祁连山) National Nature Reserve (QNR, N36°29'57′′－39°43′39′′, E97°23′34′′－103°45′49′′), in Sunan Yugur County (肃南裕固族自治县), in 2013 (Alexander, Gopalaswamy, et al., 2015) and 2017, and Minle County (民乐县) in 2017 and 2018. In Qinghai, we surveyed QNR from May 2017 to October 2018 in the counties of Tianjun (天峻县), Qilian (祁连县) and Menyuan Hui Autonomous County (门源回族自治县). QNR extends for an area of 19,872 km^2^, with maximum elevation of 5,564 m, and its habitat is mainly characterized by open sparse grass and herbaceous vegetation and shrubs, and to a lesser extent coniferous forests (Alexander, Gopalaswamy, et al., 2015) YNR and QNR hereafter will be referred to as Qilian Shan National Park (QLSNP, N36°45′16″ ‐ 39°47′14″, E94°50′7″ ‐ 102°59′9″). This park, formally established in 2018, covers a total area of 50,237 km^2^, of which 68.47% in Gansu and 31.53% in Qinghai (Qilian Shan National Park Masterplan, 2018, in Chinese) (Figure 1).

### 2.2 Presence data

We used two sources of presence data: photographic captures from remote camera trapping, and genetically verified fecal samples. In QMLNR, camera trap locations and positions were reported in Bai et al., (2018), and cover the period of 2014–2016. Fecal samples were collected in Zhalong, Jilong County (2014–2016) (Bai et al., 2018) and Zhaxizong, Dingri County (2017, Wildlife Institute at Beijing Forestry University (WIBFU), unpublished). In YNR, camera‐trapping and scat collection were conducted from 2014–2015 in Shule Nan Shan, 2017 in Yema Nan Shan and 2015–2017 in Danghe Nan Shan (WIBFU, unpublished). In QNR, Gansu, camera trapping data were collected from Qifeng area (Sunan Yugur County) (Alexander, Gopalaswamy, et al., 2015) and Minle County (WIBFU, unpublished). Surveys in Sunan Yugur County provided additional fecal samples (WIBFU, unpublished). In QNR, Qinghai, occurrence data from Tianjun, Qilian, and Meiyuan Hui Autonomous Counties were exclusively from photographic captures (WIBFU, unpublished). Details on fecal sample collection, preservation, and laboratory methods are reported in Bai et al. (2018).

### 2.3 Environmental layers

We selected 27 variables, divided into five categories (Table 1). Altitude was obtained from NASA's SRTM (Shuttle Radar Topography Mission, version 4) 3 arc‐seconds resolution digital elevation model (Jarvis, Reuter, Nelson, & Guevara, 2008). We calculated Slope Position (SLP), roughness (ROUGH), dissection (DISS), and compound topographic index (CTI) (Appendix S1) using the Geomorphometric and Gradient Metrics Toolbox 2.0 (Evans, Oakleaf, & Cushman,  2014) in ArcGIS v.10.5. Focal mean of Elevation (ELEV) was calculated using the Focal Statistics tool in ArcGIS.

**TABLE 1:** List of the 27 variables used in the current study. Each landcover class‐level metric has been calculated for each land type. Abbreviations for categories: Bare Land (Br), Grassland (Gr), Needle‐leaved forest (NLF), Shrubland (Shr), Snow and Ice (Sn)

Category	Variable	Abbreviation	Layer Source
**Linear and point features**	Density of highways and national roads	DENS_rd	Berman, 2009
	Density of human settlements	DENS_set	OpenStreetMap
	Density of rivers	DENS_riv	www.DIVA‐GIS.org
**Topographic**	Slope position	SLP	
	Focal mean of elevation	ELEV	
	Roughness	ROUGH	NASA’s SRTM v.4 (Jarvis et al., 2008)
	Compound topographic index	CTI	
	Dissection	DISS	
**Climatic**	Annual mean temperature	TEMP	WorldClim Version 2 (Fick & Hijmans, 2017)
**Landcover (Landscape level)**	Aggregation index	AI	
	Contrast‐weighted edge index	CWED	ESA GlobCover 2009 v2.3 (Arino et al., 2008)
	Patch density	PD	
**Landcover (Class Level)**	Area‐weighted mean	AREA_AM_(Br, Sn, Gr, Shr, NLF)	
	Percentage of landscape	PLAND_(Br, Sn, Gr, Shr, NLF)	ESA GlobCover 2009 v2.3 (Arino et al., 2008)
	Radius of gyration (mean area)	GYR_AM_(Br, Sn, Gr, Shr, NLF)	

We reclassified the ESA GlobCover 2009 v2.3 landcover raster (Arino et al., 2008) from 22 to 10 cover classes (Table 2), retaining the categories bare land (Br), grassland (Gr), shrubland (Shr), needle‐leaved forest (NLF), and permanent snow and ice (Sn). FRAGSTATS v 4.2 (McGarigal, Cushman, & Ene, 2012) was used to calculate three landscape‐level metrics characterizing composition, configuration, and edge contrast (Aggregation Index, AI; Patch Density, PD; Contrast Edge Weighted Density Index, CWED), and three class‐level composition and configuration level metrics (Percentage of Landscape, PLAND; Area‐weighted mean patch size, AREA_AM; patch radius of Gyration area‐weighted mean, GYR_AM) (Table 1, Appendix S2).

**TABLE 2:** Reclassification scheme of original Globcover 2009 v2.3 dataset. Reclassified categories in bold have been retained for modeling snow leopard distribution in the current study

Globcover 2009 v2.3		Reclassified	
Category	Description	Category	Description
11	Irrigated croplands	**1**	Cropland/Vegetation Mosaic
14	Rainfed croplands		
20	Mosaic Cropland/Vegetation		
30	Mosaic Vegetation/Cropland		
40	Closed to open broadleaved forest	**2**	Broadleaved Forest
50	Closed broadleaved forest		
60	Open broadleaved forest		
70	Closed needle‐leaved forest	**3**	**Needle‐leaved Forest**
90	Open needle‐leaved forest		
100	Closed to open mixed forest		
110	Mosaic Forest‐Shrubland/Grassland	**4**	**Shrubland**
120	Mosaic Grassland/Forest‐Shrubland		
130	Closed to open Shrubland		
140	Closed to open grassland	**5**	**Grassland**
150	Sparse Vegetation		
160	Closed to open broadleaved forest regularly flooded	**6**	Flooded Forest/Vegetation
170	Closed broadleaved forest permanently flooded		
180	Closed to open vegetation regularly flooded		
190	Artificial areas	**7**	Urban Areas
200	Bare Areas	**8**	**Bare Land**
210	Water bodies	**9**	Water bodies
220	Snow and Ice	**10**	**Snow and Ice**
230	No data	**11**	No data

We downloaded annual mean temperature (TEMP) from WorldClim Version 2 (Fick & Hijmans, 2017) at 30 arc‐seconds resolution. We used the National Roads and Highways of China layer (Berman, 2009) to calculate density of major traffic routes in the two landscapes. Density of rivers was calculated using inland water layers of China, downloaded from www.DIVA‐GIS.org (accessed 15/03/2018). OpenStreetMap (www.openstreetmap.org through http://download.geofabrik.de/asia/, accessed 15/03/2018) was used to download layers of human settlements, from which we retained the categories “city”, “town”, “village”, and “hamlet”, which we weighted using a scale from 4 to 1, to compute a density raster.

We resampled all variables to a UTM projection, with a 90 m cell size. Each variable was calculated at nine scales, with radii (in m) of 300, 600, 1,200, 2,400, 4,800, 9,600, 14,400, 19,200, 28,800. We chose the increments based on the original resolution of GlobCover 2009 v2.3 raster layer (300 m). We set the limit to 28,800 m to approximate a plausible daily distance moved by snow leopards (27.9 km in McCarthy, Fuller, & Munkhtsog, 2005), in absence of telemetry information from our study locations.

### 2.4 Snow leopard SDMS

#### 2.4.1 Univariate scaling

We conducted a univariate scaling for each variable (Mateo‐Sánchez et al., 2013; Vergara et al., 2015) to identify the scales most strongly related to snow leopard presence, using MaxEnt v.3.4.1 (Phillips, Anderson, Dudík, Schapire, & Blair, 2017; Phillips et al., 2006). The choice of background (pseudo‐absence points) should be based on previous ecological knowledge of the focal species (Phillips et al., 2009) and should reflect the geographic space accessible to a species in a given amount of time (Barve et al., 2011; Merow et al., 2013). Therefore, we used SDMtoolbox 2.2 (Brown, Bennett, & French, 2017) to create a buffer around occurrences with a radius of 28,800 m within which background points were selected, which is approximately the radius of snow leopard home ranges (McCarthy et al., 2005).

Where occurrences are clustered, the performance of the model can be increased by limiting the background to the fraction of the area in which presence points occur (Acevedo et al., 2012; Chefaoui & Lobo, 2008; Phillips et al., 2009). Models trained in this way tend to show better environmental potential when projected beyond the calibration areas, suggesting reduced tendency toward overfitting (Acevedo et al., 2012; Chefaoui & Lobo, 2008).

Following Vergara et al. (2015) and Mateo‐Sánchez et al. (2013), we ran MaxEnt with 20,000 background points, 5,000 iterations, linear and quadratic features, default regularization multiplier, and logistic output without a threshold. We used a 75–25 partition to train and test the models, respectively. The Area under the Curve of the Receiver Operator Characteristic (AUC curve; Fielding & Bell, 1997) was used to assess the performance of the univariate models across scales. For each predictor, we selected the scale at which the AUC value was highest. Where two scales showed equal performance in terms of AUC, we selected the scale at which the difference between the training and test partition (AUC_diff_) was minimized (Warren & Seifert, 2011). AUC_diff_ is calculated by subtracting the evaluation AUC from the calibration AUC and represents a measure of overfitting, since overfitted models tend to discriminate with great accuracy when using the training partition, but performs poorly on the test fraction of the data (Warren & Seifert, 2011). To avoid multi‐collinearity, we ran pairwise Pearson's correlations on the set of best performing scales for each variable, using the Band Collection Statistics tool in ArcGIS. When the correlation coefficient was ≥ 0.7 (Bellamy et al., 2013; Vergara et al., 2015), we retained the variables with the highest AUCs (Vergara et al., 2015).

#### 2.4.2 Multivariate models and scales comparison

The predictors retained were included in the multivariate models at the scale identified in the univariate scaling step. We built models composed of five predictors, including one variable from each category (e.g., Mateo‐Sánchez et al., 2013) (Table 1). We retained a maximum of five predictors in order to avoid overfitting of the models by adding potentially spurious variables (Mateo‐Sánchez et al., 2013; Vergara et al., 2015) and to allow comparability of the models in terms of variance explained by each of the predictors (Mateo‐Sánchez et al., 2013; Vergara et al., 2015).

To estimate how the multi‐scale optimization affected the predictive performance of each model, we compared the top performing multivariate models with models built using the same environmental predictors at each of the nine scales considered (Mateo‐Sánchez et al., 2013; Timm et al., 2016; Vergara et al., 2015). We retained the ten best multivariate models for each study area (based on AUC) and ran them in MaxEnt with the same parameters as before, with 10‐fold crossvalidation of the data and jackknifing of variables, evaluating them based on AUC and AUC_diff_.

#### 2.4.3 Sampling bias correction—simulated and real data

Simulating habitat relationships of a virtual species is often used in SDMs to assess the predictive power of modeling settings and/or bias correction methods (Fourcade et al., 2014; Hijmans, 2012; Kramer‐Schadt et al., 2013). We implemented a simulation experiment to evaluate the effects of bias intensities and performance of correction methods. We used the uncorrected top five models in each study area, at their best performing scales, as starting scenarios, considering them provisional “*reference*” models.

These reference models provided a relative suitability value at each pixel which we used to generate simulated occurrence points. This provides training data to rebuild models, given a known probability of occurrence, and to assess the performance of the models in correctly identifying the variables and scales that drive the occurrence probability, the accuracy and/or bias of the resulting predicted probability map, and performance of alternative bias correction maps.

To implement this simulation experiment we first created a uniform random raster for each study area, with values ranging from 0 to 1, and subtracted this raster from the probability surface of each of the best five reference models. We then overlaid the subtracted outputs creating a cumulative potential surface for the five models combined. On each of these raster layers, we created a cloud of 50,000 random points on the whole extent of both study areas. As these points were placed randomly by the algorithm, we selected a subset of only those occurring on pixels with positive values (representing probabilistic potentially suitable sites with values bigger than zero), creating a set of potential occurrences (4,602 in QLNSP, 555 in QMLNR). From these points, we randomly selected an equal number of presence points as the original datasets, using SDMtoolbox 2.2 (Brown et al., 2017). We thereby created two full random unbiased simulated sets of pseudo‐occurrences, representing the whole suite of potential habitats for snow leopards in the two landscapes (QLSNP_FR and QMLNR_FR).

We also produced a situation in which the pseudo‐occurrences were created with the same geographic bias as the original datasets, but were more spatially uniformly distributed (simulated‐biased datasets, QLNP_SB and QMLNR_SB). To do this, we clipped the original cloud of 50,000 random occurrences to an extent delimited by a buffer of 28,800 m radius built around the original occurrences (real datasets, QLNSP_RD and QMLNR_RD). This produced a set of 2047 points in QLSNP and 271 points in QMLNR. As described previously, we used SDMtoolbox 2.2 (Brown et al., 2017) to randomly select as many presence points as the original datasets.

To assess the effect of bias correction in three sampling scenarios, characterized by decreasing bias intensity, we used SDMToolbox 2.2 (Brown et al., 2017) to apply spatial rarefactions (SR) and Gaussian density kernel surfaces (GK) at scales of 1,200, 2,400, 4,800, and 9,600 m. Spatial rarefaction and density kernels are commonly used and highly effective correction methods in SDMs (Fourcade et al., 2014; Kramer‐Schadt et al., 2013; Mateo‐Sánchez et al., 2013; Veloz, 2009; Vergara et al., 2015). To ensure consistency with the previous steps, and place optimizations across the same training areas, we applied the two correction categories to all datasets, constraining the background to 28,800 m from the full sets of points.

We ran the RD datasets at each of the eight corrections. We further ran the top five models in each study area using the two new simulated sets of occurrences (FR and SB), with and without corrections. All MaxEnt parameters were set as described for the multivariate models’ evaluation. We assessed the performance of these simulations and bias corrections using threshold independent measures (AUC and AUC_diff_) for discrimination accuracy and overfitting proxies, and through the maximum training sensitivity plus specificity logistic threshold (MTSS) omission rate (Liu, White, & Newell, 2013; Syfert et al., 2013; Vergara et al., 2015), which is a preferable evaluation metric in presence‐only and presence‐background frameworks. We averaged all values across the five models for each bias situation (corrected and uncorrected) and dataset. Results and discussion on the simulated occurrences are provided as Appendix material.

#### 2.4.4 Sampling bias correction—niche overlap in geographic space

We anticipated that models built with different variables may respond differently to dataset type and bias correction (Randin et al., 2006), yielding different predictions (Guisan et al., 2017), even when they show similar performances based on evaluation metrics (Burnham, Anderson, & Burnham, 2002). In these circumstances, it is difficult to unequivocally rely on one single model to predict species distribution (Araújo & New, 2007; Marmion, Parviainen, Luoto, Heikkinen, & Thuiller, 2009). Since models are an approximation of a true underlying relationship, there can be many candidate models whose evaluation criteria meet the conditions required to be considered a likely representation of reality (Araújo & New, 2007; Marmion et al., 2009).

Ensembling is a modeling technique that allows combination of several optimized outputs to account for the different information generated by each prediction, allowing assessment of different methods, or different models based on the same algorithm, displaying similar performances for a given evaluation metric (Araújo & New, 2007; Guisan et al., 2017; Khosravi, Hemami, & Cushman, 2017; Marmion et al., 2009; Meller et al., 2014; Rodríguez‐Soto et al., 2011). The variation within models is thus preserved, and the modeled distributions are inferred probabilistically (Araújo & New, 2007). This approach has been applied to predict species range contraction under different climatic scenarios (Araújo, Alagador, Cabeza, Nogués‐Bravo, & Thuiller, 2011; Shirk et al., 2018), prioritize conservation measures (Meller et al., 2014; Rodríguez‐Soto et al., 2011) and evaluating niche overlaps (Khosravi et al., 2017).

**FIGURE 2:**
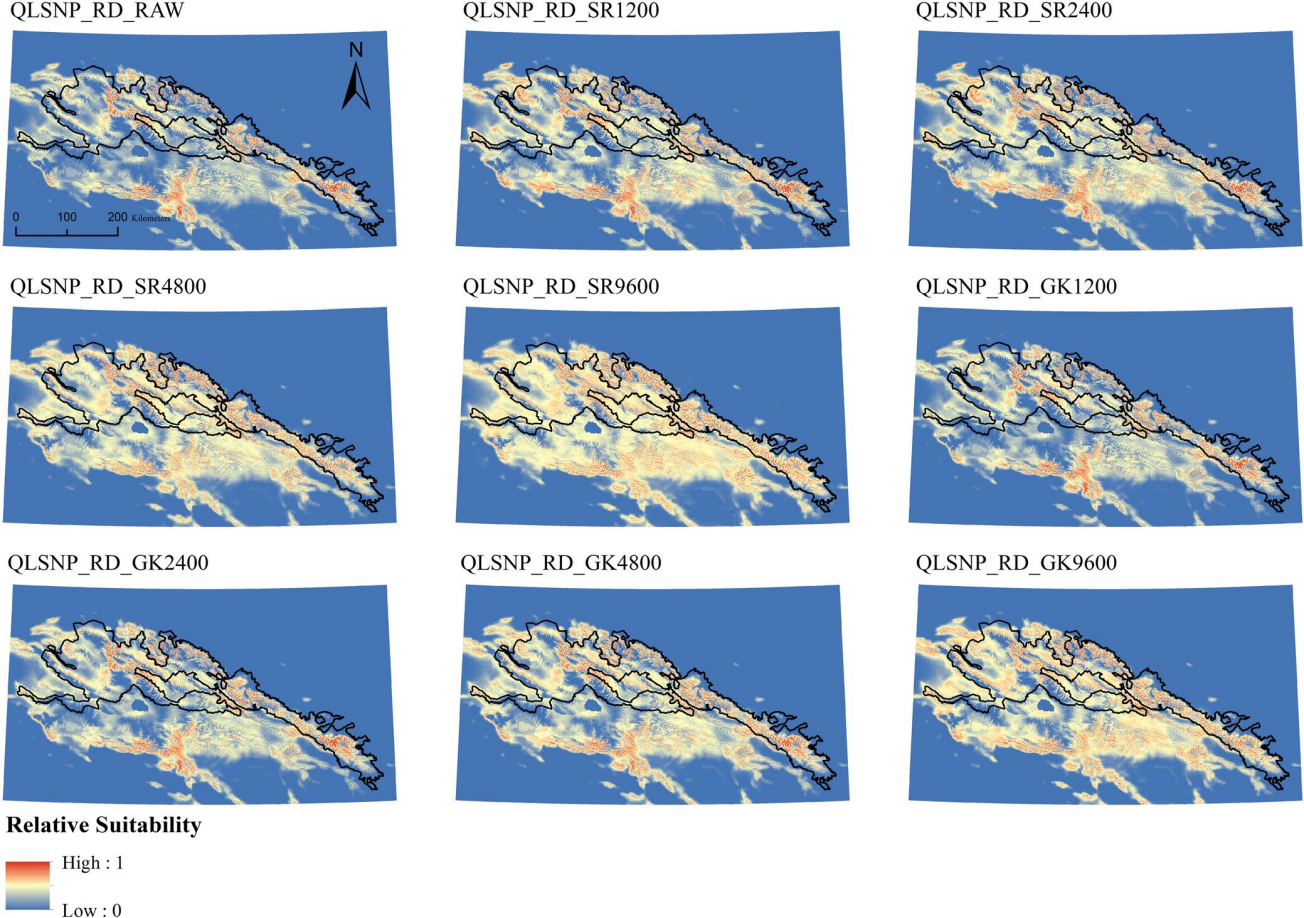
Uncorrected and corrected ensembles in Qilianshan National Park using, real occurrence data (QLSNP_RD). RAW = uncorrected model. SR = Spatial Rarefaction, followed by radius in meters. GK = Gaussian Kernel, followed by radius in meters. Inland water layers have been overlaid on top, using the color scheme indicating the lowest suitability

**FIGURE 3:**
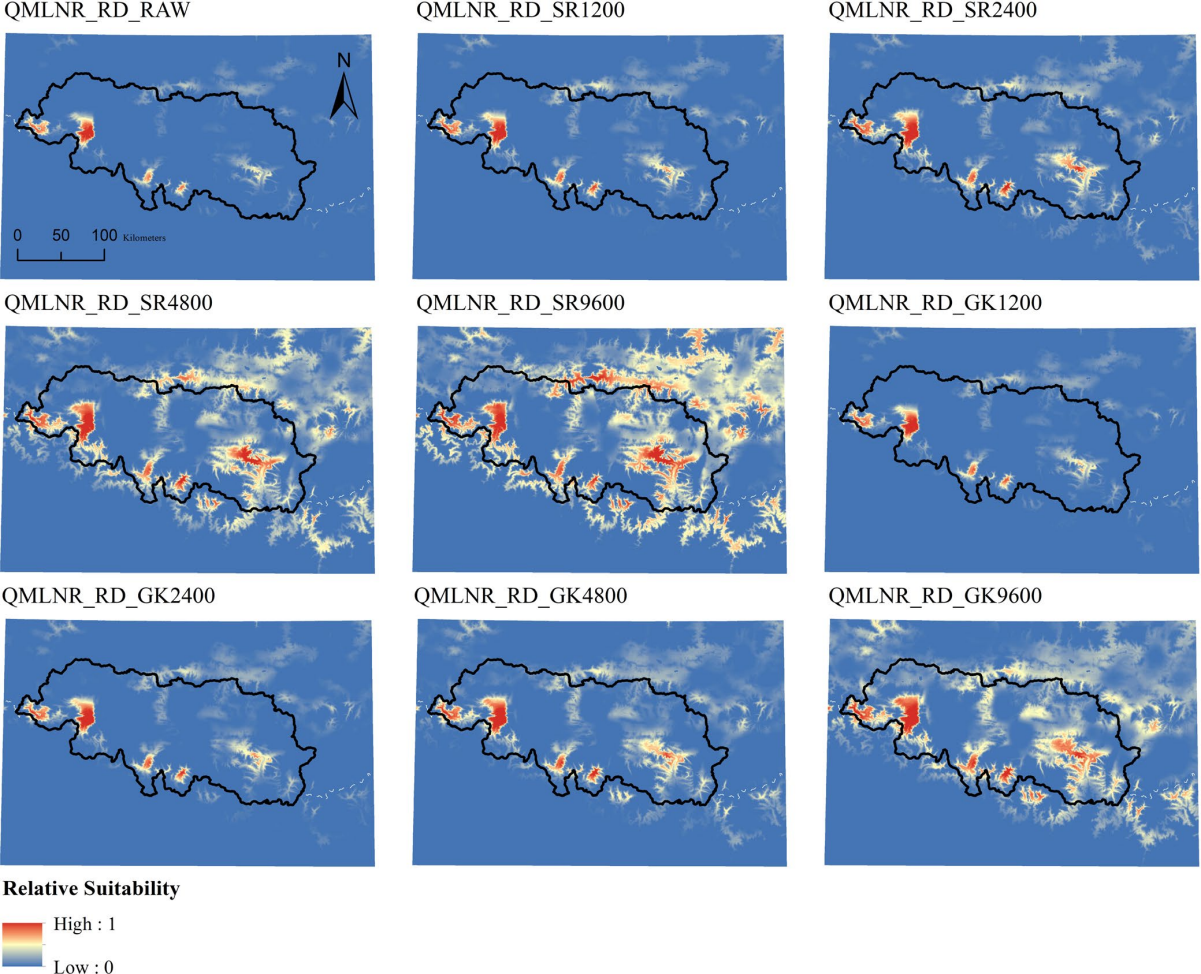
Uncorrected and corrected ensembles in Qomolangma National Nature Reserve, using real occurrence data (QMLNR_RD). RAW = uncorrected model. SR = Spatial Rarefaction, followed by radius in meters. GK = Gaussian Kernel, followed by radius in meters. Dashed white lines represent National Borders. Inland water layers have been overlaid on top, using the color scheme indicating the lowest suitability

We therefore report results from an ensembling strategy to assess which correction, across five models, would most improve the uncorrected biased scenarios (RD_RAW). For the RD datasets, we overlaid the top five models according to their correction (SR and GK) at the four radii analyzed, or absence of correction (RAW). We normalized the outputs to values ranging from 0 to 1, using the Geomorphometric and Gradient Metrics Toolbox 2.0 (Evans et al., 2014; Figures 2 and 3). We used ENMtools 1.3 (Warren, Glor, & Turelli, 2010) to calculate Schoener's D index of niche overlap (Schoener, 1968) to assess, for each ensemble, which correction would give, on average, the highest overlap with respect to a simulated, unbiased ensemble of models (FR_RAW). Before this step, raster layers in QLSNP were upscaled to 300 m cell size, in order to prevent memory failure caused by large extent and small pixel size.

The D statistic (Schoener, 1968),

$$D\left(p_{x},p_{y}\right)=1‐\frac{1}{2}\sum_{i}\left|p_{x,i}‐p_{y,i}\right|$$

describes the probability distribution of the absolute difference on the geographic space between each pixel *i* of two SDMs *x* and *y,* and ranges from 0 to 1, which is the highest degree of overlap (Warren, Glor, & Turelli, 2008). After Fourcade et al. (2014), we evaluated the performance of bias correction using the indicator ΔD_geo_,

$$\Delta D_{\text{geo}}=\left(D_{\text{corrected}}‐D_{\text{biased}}\right)/\left(1‐D_{\text{biased}}\right)$$

which expresses the degree of how an uncorrected raw model is improved after correction, with 1 corresponding to the correction yielding a model identical to an unbiased (or reference) one. We selected the best correction as the one maximally increasing ΔD_geo_, (indicating actual correction), while at the same time reducing the average MTSS omission rate with respect to the average of the uncorrected raw models (RD_RAW).

## 3 RESULTS

### 3.1 Presence data

We collected 464 and 475 fecal samples in QMLNR and QLSNP, respectively. We were able to genetically identify 68.3% and 77.4% of samples in the two study areas (Table S1). We found 134 snow leopard samples in QMLNR (28.8% of the total, 42.2% of identifications) and 230 in QLSNP (48.4% of the total and 62.5% of identifications) (Table S1). In QMLNR, snow leopards were detected in 120 out of 286 cameras in three counties (Bai et al., 2018). In YNR, snow leopards were detected by 116 out of 153 cameras (WIBFU, unpublished). In QNR, Qifeng, snow leopards were captured in 30 out of 60 cameras (Alexander, Gopalaswamy, et al., 2015). Surveys conducted continuously from May 2017 to October 2018 in Tianjun, Qilian, Menyuan Hu Counties (Qinghai Province) and Minle County (Gansu Province) consisted in a total effort of 453 cameras, of which 108 detected snow leopards. After pooling these several sources of occurrence data, we removed duplicate coordinates and samples with missing spatial information, retaining 220 occurrences in QMLNR (108 from fecal samples, 112 from cameras) and 393 in QLSNP (183 from fecal samples and 210 from cameras).

### 3.2 Predictors of snow leopard occurrence

We evaluated 243 univariate models per study area, assessing each of the 27 variables at the nine scales analyzed. In QMLNR, the majority of variables (85.1%) were selected at scales ≥14,400 m, while QLSNP scale selection exhibited more heterogeneity, with roughly 48.1% of variables at scales ≥14,400, and 33.3% from 4,800 to 9,600 m. (Table 3, Table S2).

**TABLE 3:** AUC values of the variables selected after the univariate scale selection, conducted on different predictors at each of the nine scales considered

Category	Variables	Class	QLSNP		QMLNR	
			Best scale	AUC	Best scale	AUC
Linear and Point features	DENS_riv		**4,800**	**0,627**	**19,200**	**0,794**
	DENS_rd		**19,200**	**0,613**	**28,800**	**0,748**
	DENS_set		**28,800**	**0,71**	**9,600**	**0,769**
Topographic	CTI		**9,600**	**0,741**	**14,400**	**0,726**
	SLP		**2,400**	**0,801**	**28,800**	**0,839**
	ROUGH		**600**	**0,717**	19,200	0,78
	DISS		**600**	**0,756**	14,400	0,826
	ELEV		**19,200**	**0,722**	1,200	0,819
Climatic	TEMP		**300**	**0,732**	**300**	**0,843**
Landcover (Landscape level)	CWED		4,800	0,647	**28,800**	**0,856**
	AI		**28,800**	**0,675**	**19,200**	**0,776**
	PD		**28,800**	**0,673**	**19,200**	**0,819**
Landcover (Class level)	PLAND	Br	**14,400**	**0,631**	**28,800**	**0,873**
	AREA_AM		**9,600**	**0,669**	28,800	0,878
	GYR_AM		9,600	0,639	**28,800**	**0,908**
	PLAND	Sn	9,600	0,626	9,600	0,715
	AREA_AM		9,600	0,627	**28,800**	**0,796**
	GYR_AM		**9,600**	**0,629**	28,800	0,788
	PLAND	Gr	**14,400**	**0,686**	**14,400**	**0,819**
	AREA_AM		2,400	0,628	19,200	0,807
	GYR_AM		**4,800**	**0,638**	**19,200**	**0,823**
	PLAND	Shr	19,200	0,595	14,400	0,759
	AREA_AM		**19,200**	**0,613**	**28,800**	**0,827**
	GYR_AM		19,200	0,608	28,800	0,826
	PLAND	NLF	19,200	0,568	14,400	0,754
	AREA_AM		28,800	0,573	19,200	0,767
	GYR_AM		**28,800**	**0,574**	**14,400**	**0,808**

Note

In bold the predictors selected after collinearity test (*r* ≥ 0.7).

Abbreviations: QLSNP, Qilianshan National Park; QMLNR, Qomolangma National Nature Reserve.

Differences in scales were more pronounced among topographic descriptors, selected from fine to medium scales in QLSNP and at large scales in QMLNR (Table 3). ELEV was the only topographic metric for which this trend is reversed (Table 3). There were also scale differences in the selection of area (AREA_AM) and extensiveness (GYR_AM) class‐level metrics associated with Grassland (Gr), Bare Land (Br), and Snow cover (Sn), with fine ‐medium scales in QLSNP and large scales in QMLNR. The same metrics for Shrubland (Shr) and needle‐leaved forest (NLF) showed a general agreement between the areas, being selected at coarse scales. We observed broad similarities between the two landscapes in the scales describing the response to the percentage of landscape (PLAND) relative to all landcover types at large scales (Table 3). The main difference in lansdcape‐level metrics was in terms of the scale at which contrast‐weighted edge density (CWED) was selected (medium scale in QLNSP (4,800 m) and at the largest in QMLNR (28,800 m), while aggregation (AI) and patch density (PD) metrics were selected consistently at the broadest scales across areas (28,800 m in QLSNP, 19,200 in QMLNR) (Table 3). In both landscapes, density of roads was selected at the largest scales (19,200 in QLSNP, 28,800 in QMLNR). DENS_riv was selected at an intermediate scale in QLSNP (4,800 m) and at 19,200 m in QMLNR. In contrast, density of human settlements (DENS_set) was most influential at 28,800 m in QLSNP and 9,600 m in QMLNR (Table 3). We assessed only one climatic variable (TEMP) which was selected at the smallest scale in both areas (Table 3).

### 3.3 Multivariate SDMS

We evaluated all subset combinations composed of five variables, keeping one variable per category at a time. This resulted in 210 multivariate models in QLSNP (AUC range 0.769–0.892) and 126 models in QMLNR (AUC range 0.922–0.975) (Table 4, Tables S3 and S4). The top 10 performing models showed AUC values > 0.875 in QLSNP and > 0.971 in QMLNR (Table 4, Tables S3 and S4). In QLSNP these models included variables measuring human footprint (DENS_rd_19200, two models, and DENS_set_28800, six models) and water sources (DENS_riv_4800, two models). In QMLNR, density of water sources (DENS_riv_19200) was selected in seven out of ten models, with the remaining built with DENS_set_9600. Among topographic descriptors, DISS_600 (two models) and SLP_2400 (eight models) were included in QLSNP, and CTI_14400 (eight models) and SLP_28800 (two models) in QMLNR (Table 4). PD was included in only one model in each study area. The remaining top models were built with AI_28800 in QLSNP and CWED_28800 in QMLNR. Among landscape categories, metrics associated with Gr (GYR_AM_Gr_4800, PLAND_Gr_14400) were selected in nine out of ten models in QLSNP, with the remaining built with AREA_AM_Shr_19200. In QMLNR, five models were built with metrics associated with Gr (PLAND_Gr_14400, GYR_AM_ Gr_19200) four with Br (PLAND_Br_28800, GYR_AM_Br_28800) and one with NLF (GYR_AM_NLF_14400) (Table 4).

**TABLE 4:** Ten top performing multivariate models in each study area, ranked by performance of AUC and AUC_diff_ values

Model number	Linear/point features	Topographic	Climatic	Landcover Landscape level	Landcover Class level	AUC test	AUC_diff_
QLSNP							
QLSNP_1	DENS_set_28800	SLP_2400	TEMP_300	AI_28800	GYR_AM_Gr_4800	0,892	−0,027
QLSNP_2	DENS_set_28800	SLP_2400	TEMP_300	AI_28800	PLAND_Gr_14400	0,89	−0,018
QLSNP_3	DENS_riv_4800	SLP_2400	TEMP_300	AI_28800	GYR_AM_Gr_4800	0,887	−0,022
QLSNP_4	DENS_riv_4800	SLP_2400	TEMP_300	AI_28800	PLAND_Gr_14400	0,886	−0,011
QLSNP_5	DENS_set_28800	SLP_2400	TEMP_300	PD_28800	GYR_AM_Gr_4800	0,881	−0,033
QLSNP_6	DENS_rd_19200	SLP_2400	TEMP_300	AI_28800	PLAND_Gr_14400	0,879	0,001
QLSNP_7	DENS_rd_19200	SLP_2400	TEMP_300	AI_28800	GYR_AM_Gr_4800	0,878	−0,005
QLSNP_8	DENS_set_28800	DISS_600	TEMP_300	AI_28800	GYR_AM_Gr_4800	0,877	−0,020
QLSNP_9	DENS_set_28800	DISS_600	TEMP_300	AI_28800	PLAND_Gr_14400	0,875	−0,016
QLSNP_10	DENS_set_28800	SLP_2400	TEMP_300	AI_28800	AREA_AM_Shr_19200	0,875	−0,015
QMLNR							
QMLNR_1	Dens_riv_19200	CTI_14400	TEMP_300	CWED_28800	PLAND_Gr_14400	0,975	0,002
QMLNR_2	Dens_riv_19200	CTI_14400	TEMP_300	CWED_28800	GYR_AM_ Gr_19200	0,973	−0,001
QMLNR_3	Dens_riv_19200	SLP_28800	TEMP_300	CWED_28800	PLAND_Br_28800	0,972	−0,004
QMLNR_4	Dens_riv_19200	CTI_14400	TEMP_300	CWED_28800	GYR_AM_Br_28800	0,972	0,000
QMLNR_5	Dens_riv_19200	CTI_14400	TEMP_300	CWED_28800	GYR_AM_NLF_14400	0,972	0,001
QMLNR_6	Dens_riv_19200	CTI_14400	TEMP_300	PD_19200	PLAND_Gr_14400	0,972	0,002
QMLNR_7	Dens_set_9600	CTI_14400	TEMP_300	CWED_28800	PLAND_Gr_14400	0,972	0,003
QMLNR_8	Dens_riv_19200	SLP_28800	TEMP_300	CWED_28800	GYR_AM_Br_28800	0,971	−0,008
QMLNR_9	Dens_set_9600	CTI_14400	TEMP_300	CWED_28800	GYR_AM_ Gr_19200	0,971	−0,001
QMLNR_10	Dens_set_9600	CTI_14400	TEMP_300	CWED_28800	GYR_AM_Br_28800	0,971	0,000

Abbreviations: QLSNP, Qilianshan National Park; QMLNR, Qomolangma National Nature Reserve.

### 3.4 Multi‐scale versus single‐scale models

We assessed the ten best multivariate models at each of the nine scales considered and compared their performances with those of their multi‐scale equivalents. This produced 100 models per study area. We observed that multi‐scale models had the highest discrimination ability in almost all cases (Table 5). In all but two models in QLSNP (QLSNP_5 and QLSNP_10), the multi‐scale evaluation increased AUC values, outperforming the models built with the same variables at a fixed scale. In this area, the top performing single scale was 2,400 for all models except QLSNP_4, QLSNP_8, and QLSNP_9, whose best single scale was 600 m. In QMLNR, all models except QMLNR_8, exhibited the highest performance using the multi‐scale approach. In this study area, the best performing single scale was 19,200 m in all cases except QMLNR_9, which was at 14,400 m (Table 5). Averages of the ten models highlighted the overall higher discrimination ability of multi‐scale approach in both study areas, compared to unscaled models, with the poorest performing scales being 28,800 m in QLSNP and 300 m in QMLNR, and average best single scales of 1,200 m in QLSNP and 19,200 in QMLNR. In both study areas, the top performing five models were always selected in their multi‐scale versions. AUC values for these models ranged 0.853–0.864 in QLSNP and 0.97–0.975 in QMLNR (Table 5).

**TABLE 5:** Comparison of performance between multi‐scale models and their corresponding unscaled models for each of the scales considered, evaluated through AUC and AUC_diff_. Values in bold represent the best performing scale for that model. Models marked with an asterisk represent the five best models in terms of AUC evaluation metric

	Multi‐scale		300		600		1,200		2,400		4,800		9,600		14,400		19,200		28,800	
	AUC test	AUC**_diff_**	AUC test	AUC**_diff_**	AUC test	AUC**_diff_**	AUC test	AUC**_diff_**	AUC test	AUC**_diff_**	AUC test	AUC**_diff_**	AUC test	AUC**_diff_**	AUC test	AUC**_diff_**	AUC test	AUC**_diff_**	AUC test	AUC**_diff_**
**QLSNP**																				
QLSNP_1*	**0.853**	0.008	0.815	0.005	0.833	0.004	0.844	0.004	0.848	0.003	0.83	0.002	0.809	0.003	0.796	0.003	0.795	0.003	0.776	0.008
QLSNP_2*	**0.856**	0.009	0.815	0.005	0.831	0.005	0.839	0.004	0.844	0.003	0.83	0.002	0.809	0.002	0.795	0.003	0.792	0.003	0.774	0.007
QLSNP_3	**0.851**	0.008	0.823	0.005	0.839	0.005	0.844	0.004	0.847	0.004	0.831	0.003	0.825	0.004	0.823	0.006	0.807	0.006	0.777	0.009
QLSNP_4*	**0.856**	0.009	0.791	0.006	0.808	0.004	0.807	0.009	0.802	0.009	0.79	0.004	0.783	0.004	0.781	0.004	0.766	0.004	0.743	0.004
QLSNP_5	0.845	0.005	0.816	0.005	0.834	0.005	0.837	0.005	**0.848**	0.004	0.828	0.003	0.81	0.003	0.794	0.003	0.787	0.553	0.765	0.006
QLSNP_6*	**0.864**	0.008	0.815	0.005	0.831	0.005	0.839	0.004	0.844	0.003	0.831	0.003	0.814	0.002	0.804	0.003	0.808	0.003	0.784	0.007
QLSNP_7*	**0.859**	0.008	0.815	0.005	0.832	0.005	0.843	0.004	0.849	0.003	0.83	0.003	0.813	0.003	0.807	0.003	0.808	0.004	0.783	0.007
QLSNP_8	**0.848**	0.007	0.83	0.003	0.841	0.002	0.839	0.002	0.817	0.003	0.778	0.003	0.766	0.003	0.777	0.003	0.793	0.003	0.793	0.008
QLSNP_9	**0.849**	0.006	0.83	0.003	0.84	0.002	0.837	0.003	0.814	0.003	0.778	0.002	0.765	0.002	0.78	0.004	0.795	0.004	0.795	0.008
QLSNP_10	0.842	0.009	0.816	0.005	0.829	0.005	0.841	0.003	**0.847**	0.004	0.839	0.002	0.813	0.002	0.792	0.003	0.789	0.003	0.778	0.007
*Average*	0.852	0.008	0.817	0.005	0.832	0.004	0.837	0.004	0.836	0.004	0.817	0.003	0.801	0.003	0.795	0.004	0.794	0.059	0.777	0.007
**QMLNR**																				
QMLNR_1*	**0.975**	0.001	0.903	0.004	0.905	0.003	0.912	0.003	0.914	0.002	0.9	0.003	0.946	0.002	0.964	0.002	0.967	0.003	0.944	0.002
QMLNR_2*	**0.971**	0.001	0.902	0.003	0.909	0.003	0.913	0.003	0.912	0.004	0.898	0.005	0.927	0.002	0.954	0.002	0.958	0.003	0.933	0.003
QMLNR_3	**0.967**	0.001	0.881	0.008	0.887	0.006	0.888	0.003	0.871	0.008	0.851	0.007	0.892	0.004	0.951	0.002	0.963	0.003	0.957	0.002
QMLNR_4*	**0.97**	0.001	0.886	0.005	0.899	0.004	0.917	0.002	0.924	0.005	0.929	0.004	0.939	0.003	0.957	0.002	0.962	0.003	0.949	0.002
QMLNR_5*	**0.971**	0.002	0.89	0.004	0.909	0.003	0.921	0.003	0.933	0.003	0.935	0.002	0.923	0.007	0.954	0.003	0.961	0.002	0.931	0.004
QMLNR_6*	**0.972**	0.001	0.9	0.002	0.9	0.002	0.91	0.004	0.918	0.003	0.908	0.002	0.946	0.002	0.956	0.003	0.963	0.002	0.938	0.001
QMLNR_7	**0.969**	0.005	0.9	0.004	0.907	0.003	0.917	0.002	0.914	0.002	0.9	0.003	0.939	0.004	0.953	0.006	0.957	0.004	0.934	0.005
QMLNR_8	0.963	0.001	0.897	0.004	0.888	0.005	0.884	0.006	0.876	0.009	0.885	0.003	0.923	0.003	0.958	0.001	**0.964**	0.003	0.957	0.003
QMLNR_9	**0.966**	0.004	0.903	0.003	0.906	0.002	0.915	0.002	0.912	0.003	0.901	0.004	0.918	0.006	0.946	0.003	0.943	0.01	0.922	0.006
QMLNR_10	**0.967**	0.004	0.886	0.005	0.901	0.003	0.915	0.003	0.924	0.005	0.93	0.003	0.934	0.006	0.95	0.003	0.958	0.005	0.939	0.004
*Average*	0.969	0.002	0.895	0.004	0.901	0.003	0.909	0.003	0.910	0.004	0.904	0.004	0.929	0.004	0.954	0.003	0.960	0.004	0.940	0.003

Abbreviations: QLSNP, Qilianshan National Park; QMLNR, Qomolangma National Nature Reserve.

### 3.5 Bias correction and overlap with reference unbiased models

Here, we present results from real datasets (RD), with details of correction methods across the three different bias situations provided in Appendix S4. We ran 40 models per study area, evaluating the eight correction methods (Figures 2 and 3; Table 6, Appendix S4) on real datasets (RD) for each of the top five models. These correction methods consisted of four radii of spatial rarefaction (SR) and four radii of Gaussian density kernels (GK), as explained in the Methods section.

**TABLE 6:** Performances of correction methods, reported as average of the five top models, for the real datasets (RD), in Qilianshan National Park (QLSNP) and Qomolangma National Nature Reserve (QMLNR). MTSS = Maximum training sensitivity plus specificity logistic threshold; MTSS om = omission rate for MTSS threshold. D represents Schoener's D (Schoener, 1968) niche overlap index, calculated for each ensemble and dataset with respect to FR_RAW ensemble of models (Full Random dataset, FR)

Dataset	Models	*N*	AUC	AUC_diff_	MTSS	MTSS om	D	ΔD_geo_
QLSNP								
RD	QLSNP_RD_RAW	393	0,858	0,008	0,398	0,271	0,810	0,000
	QLSNP_RD_SR1200	219	0,837	0,009	0,415	0,272	0,841	0,166
	QLSNP_RD_SR2400	158	0,819	0,009	0,424	0,290	0,858	0,254
	QLSNP_RD_SR4800	103	0,766	0,023	0,443	0,421	0,892	0,433
	QLSNP_RD_SR9600	65	0,756	0,034	0,453	0,407	0,921	0,584
	QLSNP_RD_GK1200	393	**0,860**	0,009	0,417	**0,268**	0,822	0,066
	QLSNP_RD_GK2400	393	**0,862**	0,009	0,429	**0,262**	0,835	0,131
	***QLSNP_RD_GK4800***	393	**0,863**	0,009	0,448	**0,255**	0,860	0,267
	QLSNP_RD_GK9600	393	0,855	0,009	0,471	0,290	0,892	0,433
QMLNR								
RD	QMLNR_RD_RAW	220	0,972	0,001	0,194	0,085	0,572	0,000
	QMLNR_RD_SR1200	79	0,958	0,004	0,188	0,126	0,667	0,221
	QMLNR_RD_SR2400	49	0,943	0,007	0,212	0,115	0,780	0,486
	QMLNR_RD_SR4800	28	0,905	0,015	0,331	0,190	0,844	0,636
	QMLNR_RD_SR9600	19	0,904	0,015	0,316	0,150	0,759	0,437
	QMLNR_RD_GK1200	220	0,971	**0,001**	0,263	**0,085**	0,646	0,173
	QMLNR_RD_GK2400	220	0,971	**0,001**	0,321	**0,085**	0,699	0,297
	QMLNR_RD_GK4800	220	0,971	0,002	0,432	**0,082**	0,763	0,445
	***QMLNR_RD_GK9600***	220	0,968	0,002	0,501	**0,081**	0,768	0,457

Note

ΔD_geo_ calculated using D overlap with FR_RAW ensemble of models (ΔD_geo_=(D_corrected_‐D_biased_)/(1‐D_biased_); Fourcade et al., 2014). Values in bold represent improvement or equal performance with respect to the raw models. The model selected as the best correction (maximizing D, with positive ΔD_geo_ while reducing MTSS omission rate) is reported in bold and *italic*.

AUC values decreased consistently across all radii of rarefaction in the two study areas (Table 6). The opposite was true for GKs, reaching similar performance to raw models (QMLNR) or improving the discrimination ability (QLSNP) (Table 6). AUC_diff_ values were not maximal for the raw models (Veloz, 2009; Boria et al., 2014, Radosavljevic and Anderson, 2014; Vergara et al., 2015), as we applied a restricted background in order to increase accuracy of the prediction (Acevedo et al., 2012; Chefaoui & Lobo, 2008; Phillips et al., 2009). On average though, large radii of SR consistently led to an increase of this metric, while small SR radii and GKs caused minimal change (Table 6). MTSS omission rate was always increased by rarefaction, although SR1200 in QLSNP caused a minimal increase with respect to the average of raw models. GKs on average were more robust in reducing MTSS omission rate at all radii in the two areas, except for the largest radius in QLSNP (Table 6).

**FIGURE 4:**
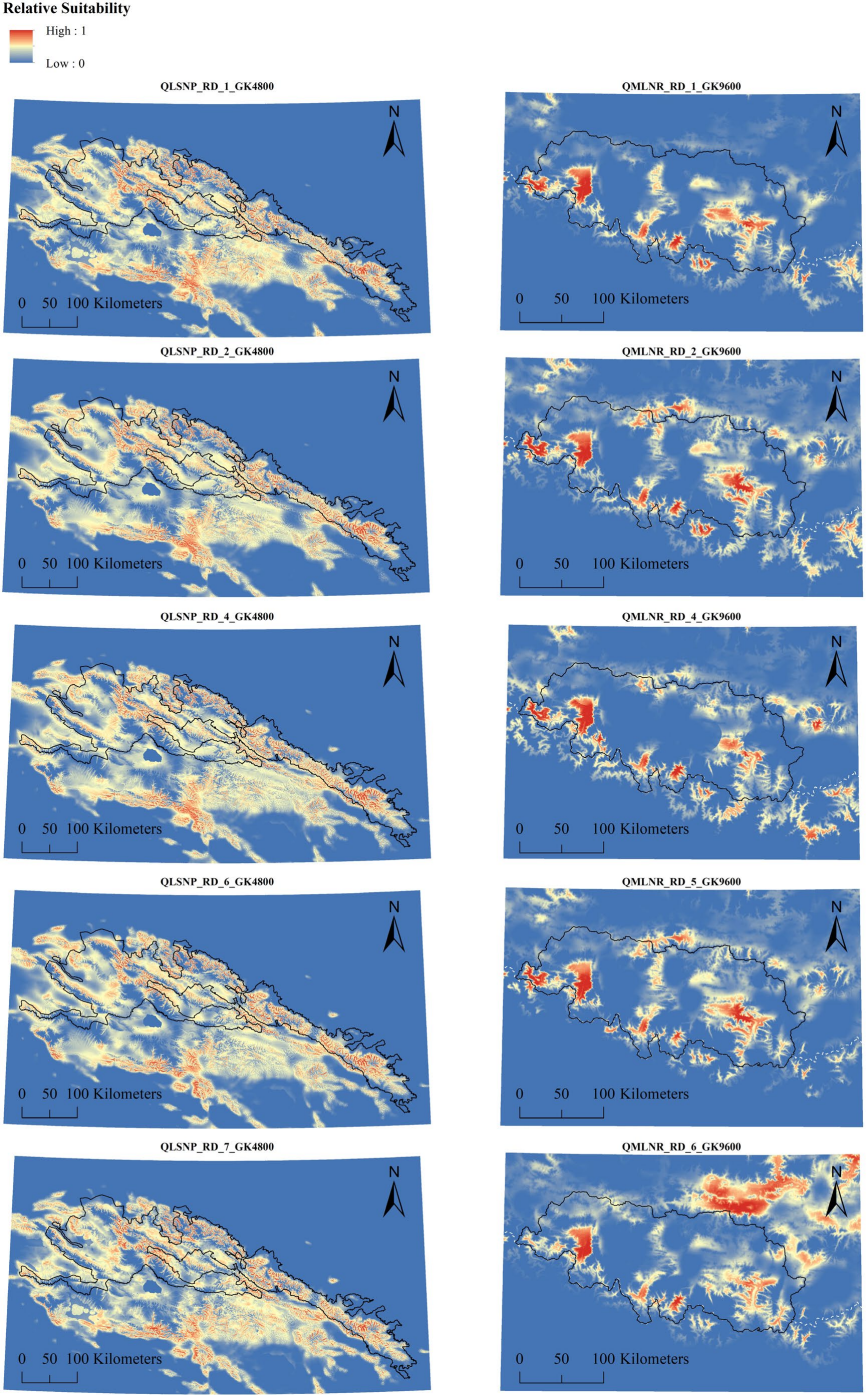
Top five models in each study area, shown at their best average correction. For details about variables used in each model, and best average correction, refer to the main text and Tables 4, 5, 6. QLSNP = Qilianshan National Park; QMLNR = Qomolangma National Nature Reserve. Dashed white lines represent National borders in QMLNR. Inland water layers have been overlaid on top, using the color scheme indicating the lowest suitability

In QLSNP_RD there was a steady increase in overlap based on D and ΔD_geo_ as the radius of SR increased, reaching the highest value at a distance of 9,600 m (QLSNP_RD_SR9600, D = 0.921, ΔD_geo_ = 0.584), but characterized by a high omission rate (0.407) (Table 6). A kernel of intermediate radius (QLSNP_RD_GK4800) achieved highest overlap and optimal improvement for the second correction type (*D* = 0.860, Δ*D*_geo_ = 0.267) and an average omission rate lower than the average of the uncorrected models (0.255) (Figures 2 and 4, Table 6). In the QMLNR_RD dataset, SR4800 (*D* = 0.844, Δ*D*_geo_ = 0.636, MTSS om = 0.190) and GK9600 (*D* = 0.768, Δ*D*_geo_ = 0.457, MTSS om = 0.081) maximized the overlap in geographic space with respect to the ensemble of QMLNR_FR_RAW models, with QMLNR_RD_GK9600 representing the best average correction (Figures 3 and 4, Table 6).

### 3.6 Environmental predictors of snow leopard occurrence

**FIGURE 5:**
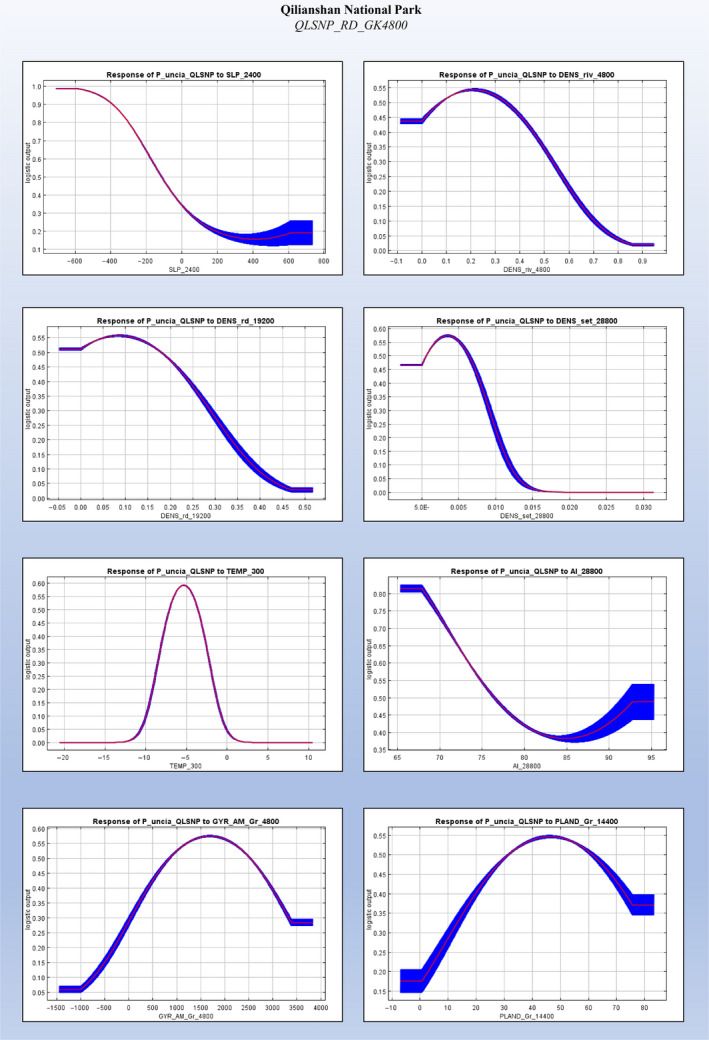
Response curves for each of the unique variables included in the top five models, in Qilianshan National Park (QLSNP), shown at their best correction. These curves represent snow leopard response when each variable is tested without interactions with other predictors

**FIGURE 6:**
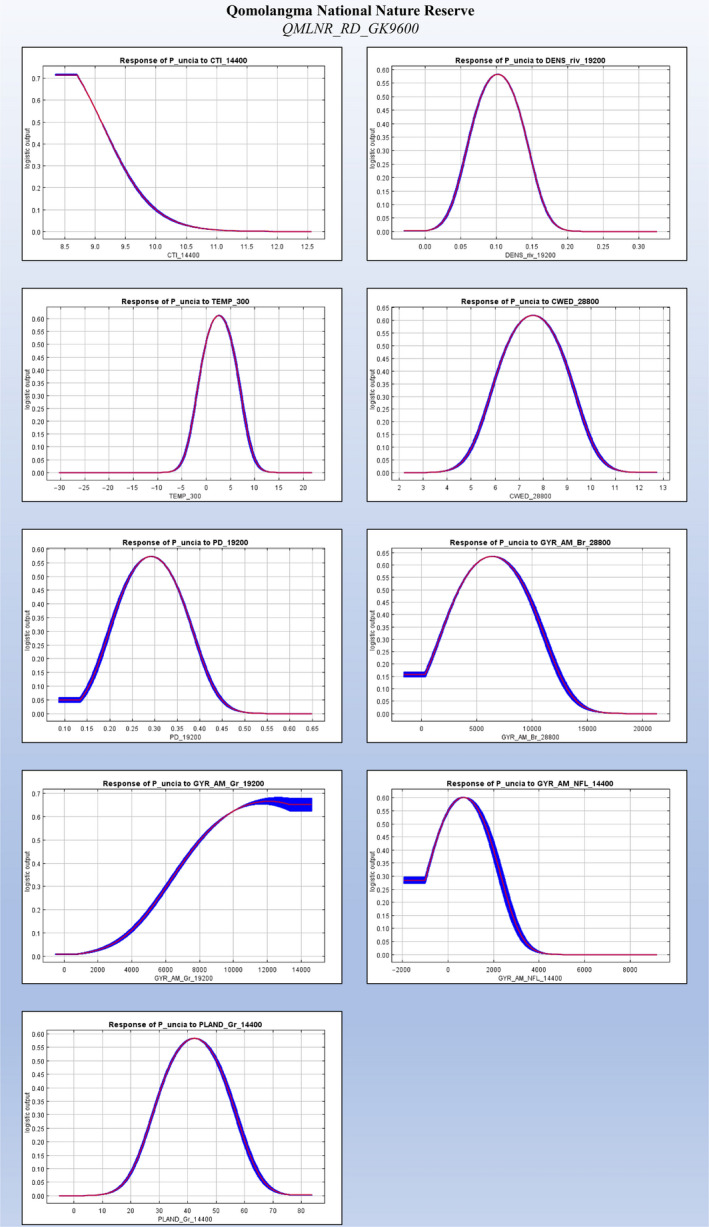
Response curves for each of the unique variables included in the top five models, in Qomolangma National Nature Reserve (QMLNR), shown at their best correction. These curves represent snow leopard response when each variable is tested without interactions with other predictors

Based on the results of the top performing corrected models (QLSNP_RD_GK4800 and QMLNR_RD_GK9600), temperature (TEMP_300) in both landscapes showed a unimodal peak of support, representing a key factor for habitat suitability (28.3%–34.8% contribution in QLSNP, 37.4%–50.8% in QMLNR), and emphasizing the influence of abiotic and climatic gradients in determining the distribution of snow leopards (Tables S5 and S6, Figures 5 and 6). Other important predictors were associated with topography. In QLNSP, snow leopard relative probability of occurrence was related to negative values of SLP_2400 (55.6%–59.1% contribution), while in QMLNR it steadily decreased at CTI_14400 values higher than 8.5 (8.6%–28.7% contribution) (Tables S5 and S6, Figures 5 and 6). In QLSNP, 65%–70% of landscape aggregation (AI_28800, 2.5%–3.6%), an extensiveness of 1500–2000 m (GYR_AM_Gr_4800, 3.4%–4.7% contribution) and 40%–50% of landscape (PLAND_Gr_14400, 1.1%–1.4% contribution) composed by herbaceous vegetation were most strongly associated with snow leopard presence (Table S5, Figure 5). In QMLNR, higher probability of occurrence was associated with 7–8 m of edge per hectare (CWED_28800, 3.2%–15.6% contribution), and approximately 0.3 patches per hectare (PD_19200, 18.3% contribution). In this area, habitat associations revealed the importance of long extents of barren (GYR_AM_Br_28800, 32.4% contribution, 5,000–7,000 m) and grassland areas (GYR_AM_Gr_19200, 37.2%, more than 10 km), which roughly represented the 40%–45% of the landscape at the best radius (PLAND_Gr_14400, 3%–12.6% contribution) (Table S6, Figure 6). Response curves in QLSNP showed higher occurrence patterns at low density of roads (DENS_rd_19200, 7.5%–7.7%) and human settlements (DENS_set_28800, 5.7%–6%). Association with rivers showed similar values across the study areas, with densities of approximately 0.2 km^2^ (DENS_riv_4800, 1.5% contribution) and 0.1 km^2^ (DENS_riv_19200, 0.9%–1.7%) in QLSNP and QMLNR, respectively (Tables S5 and S6, Figures 5 and 6). Finally, the association with small extents of GYR_AM_NLF_14400 in QMLNR (19.3% contribution) indicates an important role of a mosaic of coniferous forest patches in this area as a component of snow leopard habitat, possibly related to prey diversity or abundance.

## 4 DISCUSSION

### 4.1 Overview of main results

Our study addressed four main issues. (a) What is the scale‐dependent relationship between snow leopard occurrence and habitat variables in western China, (b) are those relationships stable and comparable between two different study areas with different limiting factors, (c) where they differ between study areas, can the differences be explained by different limiting factors, and (d) what is the performance of commonly used spatial bias correction methods in improving prediction of snow leopard habitat selection. Here we briefly describe the main implications of our results for each of these points, with elaboration of specific details in the following paragraphs. First, our models showed strong performance in predicting snow leopard presence points in both landscapes, with AUC values 0.87–0.97 across study areas, indicating very good prediction. This suggests that snow leopards in the study areas have strong discrimination in their selection of habitat, and that our modeling was successful in being able to describe that discrimination. Second, our models also show that the multi‐scale modeling optimization improved prediction in nearly all cases, and that multi‐scale optimization (sensu McGarigal et al., 2016) is important to obtain the most accurate predictions of snow leopard habitat quality, as it has been shown to be for other felids (e.g., Ashrafzadeh et al., 2020; Elliot et al., 2014; Hearn et al., 2018; Krishnamurthy et al., 2016; Macdonal et al., 2018, 2019). Third, similar to those previous multi‐scale felid habitat papers, we found that most habitat variables were selected by snow leopards at broad scales, reflecting both the high mobility and large home range size of the species, and its high sensitivity to human and other habitat perturbations at broad scales, similar to other carnivores (e.g., Mateo‐Sánchez et al., 2013; Wasserman, Cushman, Wallin, et al., 2012). Fourth, we found a relatively high degree of agreement between the two study areas in terms of variables and scales, with temperature and large extents of grass and sparsely vegetated conditions in upland and ridge topographic settings important for snow leopards in both landscapes. Fifth, the differences we did observe between study areas seemed to be related to differences in the limiting factors in those particular landscapes (e.g., Cushman et al., 2011; Shirk et al., 2014; Short bull et al., 2011). Specifically, variables tended to be selected at broader scales in the landscape that was more homogeneous, and at finer scales in the more topographically and ecologically complex landscape, suggesting that scale of variable selection is related to the scale at which each variable is heterogeneous and thus potentially limiting, as also seen by previous studies (e.g., Short Bull et al., 2011). Sixth, our results clearly show there is potentially serious impact of spatial bias in presence‐only models, and similar to past findings (e.g., Vergara et al., 2015), we found that Gaussian kernel methods of bias correction out performed other spatial filtering or rarefaction approaches in all scenarios we evaluated.

### 4.2 Multivariate model performance

Our multivariate model selection framework was successful in identifying the combinations of variables able to describe habitat for snow leopard with higher discrimination ability (Tables S3 and S4). By retaining only five predictors per model we excluded the possibility of adding spurious variables with null contribution (e.g., Mateo‐Sánchezet al., 2013; Vergara et al., 2015; Appendix S3). Moreover, by selecting only one factor per category, we determined the influence of each habitat feature when assessed together with variables of other kinds, without the risk of losing descriptive power due to the interaction of components describing the same habitat characteristics (i.e., topographic descriptors). Given the differences between top and least performing multivariate models (Tables S3 and S4), we could successfully identify the landscape‐specific most influential variables, which were more accurate in describing the ecological associations driving habitat selection for snow leopards, which was most strongly related to peculiar habitat features. This cautions against the use of a fixed suite of descriptors for snow leopard habitat. For example, past studies have emphasized the importance of topographic roughness and elevation as components of snow leopard habitat. However, in our two study areas these variables are not major limiting factors to occurrence patterns for snow leopards, but may still be critical components of habitat (e.g., Cushman et al., 2011; Cushman, Shirk, & Landguth, 2013). Failure to account for this would result in models with lower discriminatory power and possibly incorrect assessment of key habitat components determining the true occurrence patterns, as well as the misspecification of true ecological interactions (Tables S3 and S4).

### 4.3 Scales affecting snow leopards distribution

Our results show the emergence of snow leopard habitat selection at multiple scales for different variables in the same landscape, and for the same variables across different landscapes, with locally specific topographic and landcover heterogeneity factors determining those scales, and the vast majority of variables selected at medium‐coarse scales across the areas (Table 3). Large territorial carnivores are expected to select habitat features at mostly broad scales, reflecting their mobility (Elliot et al., 2014; Krishnamurthy et al., 2016) and home range requirements (Ashrafzadeh et al., 2020; Hearn et al., 2018; Khosravi et al., 2019; Macdonald et al., 2018, 2019; Mateo‐Sánchez et al., 2013). In the case of the snow leopards, the overall broad‐scale response to habitat features is further driven by the low productivity of the cold xeric environments typical of the mountainous habitats in their range, determining scales of effect greater than would be expected upon a relationship with body size alone (Fisher, Anholt, & Volpe, 2011; Tucker & Rogers, 2014).

As a recurring pattern in studies of habitat selection by large carnivores (Ashrafzadeh et al., 2020; Elliot et al., 2014; Hearn et al., 2018; Khosravi et al., 2019; Macdonald et al., 2018, 2019; Mateo‐Sánchez et al., 2013) we observed medium‐broad scales negative association with variables expressing human footprint (Table 3), either represented by density of settlements or transport infrastructures. Snow leopards select their habitats restricting their use of topographic features to ridges and dry uplands to minimize human disturbance (Figures 5 and 6). These patterns have been seen in other felids, which generally select higher elevation areas and rugged terrain as a way to minimize the risk of conflicts with humans (Hearn et al., 2018; Krishnamurthy et al., 2016; Macdonald et al., 2018, Reddy et al., 2017; Reddy, Puyravaud, Cushman, & Segu, 2019).

We found selection of riverine features at medium‐coarse scales, with finer scale of selection indicative of increased topographic complexity, likely resulting in a denser array of seasonal streams, and the coarser scale ultimately dependent on major hydrological processes operating at landscape level (Table 3). Where rivers are more abundant, snow leopards are associated with a slightly higher density of such features (Figures 5 and 6), whose riverbeds might offer access to easier dispersal routes (besides ensuring ambush opportunities) in a terrain with increased topographic ruggedness and dissection (Table 3). Where rivers are less abundant, the coarse scale association might be indicative also of a secondary relationship with human disturbance, as many settlements in QMLNR are found in the proximity of these major watercourses (Table 3).

Broad ecological associations are highlighted not only by the scale at which landscape composition metrics are selected (Table 3), but also by their magnitude (Figures 5 and 6), which confirm snow leopard preference for largely connected contiguous habitats. The steady cross‐area coarse scale response with regards to the relative abundance of habitat types (PLAND) and similarity of their scales between study areas (Table 3), suggest that snow leopards consistently select an optimal amount of such habitat features at broad scales. This possibly implies ecological domains (Levin, 1992; Wiens, 1989) and confirms also the role of the area and extensiveness of key habitat patches (Tables 3 and 4, Figures 5 and 6) in supporting populations persistence, especially important in elusive territorial carnivores (Hearn et al., 2018; Macdonald et al., 2018, 2019; Mateo‐Sánchez et al., 2013). Finally, the general coarse scale selection toward sparse habitat features such shrub and forest patches, is a function of their low overall variability and representativity, being aggregated in smaller portions on the entire extents (Table 3).

### 4.4 Performance of multi‐ and single‐scale models

This study provides further evidence that, when habitat selection for different features operates at several scales (Levin, 1992; McGarigal et al., 2016; Wiens, 1989) (Table 3), the incorporation of landscape‐specific scales provides a more accurate (and ecologically realistic) description of snow leopard habitat, compared to any approach in which the suite of predictors included in a model is held at a fixed scale (Mateo‐Sánchez et al., 2013; Shirk et al., 2012; Timm et al., 2016; Vergara et al., 2015; Wan et al., 2017; Wasserman, Cushman, Wallin, et al., 2012). The multi‐scale modeling approach was superior to the unscaled counterparts in almost all cases (Table 5), being thus able to identify the magnitude of effect of locally influential factors (Appendix S3), and their role in determining model performance (Table 5, Appendix S3).

There can be cases however in which unscaled models models might reach a performance similar to multi‐scale models (Elliot et al., 2014; Graf, Bollmann, Suter, & Bugmann, 2005; Krishnamurthy et al., 2016; Martin & Fahrig, 2012). Reasons for such situations have been extensively investigated by Martin and Fahrig (2012) and are intrinsically dependent on the effect that a given variable has on species ecology at any of its focal grains, in relation to the species’ life cycle.

Models built with fixed‐scale variables might provide a surrogate description of habitat, performing almost as well as the true multi‐scale models (Table 5, Appendix S3), when their variables lie within a narrow range of scale cross‐correlation with the best respective scale (Martin & Fahrig, 2012). We found evidence of this situation in QMLNR, where the best unscaled models are built at scales highly correlated with the best scales for all the predictors. In other circumstances, species might select a given predictor at an optimum scale, which has however strong cross‐scale correlation. As in the case of TEMP in QLSNP, whether such variables have exceedingly high effect on model performance (Appendix S3), unscaled models reach a good discrimination ability, even when other predictors selected at different scales, and having specific narrow cross‐scale correlation range (indicative of true multiple scale response (Martin & Fahrig, 2012)), do not contribute to the models or do it marginally (Appendix S3).

In unscaled models, a given variable can mask the effect of locally important predictors, when these are not included at their best scales, resulting in an incorrect specification of the factors driving the landscape‐specific distribution, and neglecting their contribution altogether (Appendix S3). Previous analyses have demonstrated how single‐scale models have tendency to overpredict relative probability of occurrence in areas of low suitability, and underpredict it in areas of high suitability (e.g., Mateo‐Sánchez et al., 2013; Shirk et al., 2012; Wasserman, Cushman, Shirk, Landguth, & Littell, 2012). Therefore, as supported by this and other studies (Ashrafzadeh et al., 2020; Hearn et al., 2018; Khosravi et al., 2019; Macdonald et al., 2018, 2019; Mateo‐Sánchez et al., 2013; Timm et al., 2016; Vergara et al., 2015; Wan et al., 2017) it is more effective to employ a multiple scale optimization as a description of species habitat, as it will identify the best multiple or single scales, as the case may be.

### 4.5 Snow leopards ecological associations across study areas

Our best multivariate models (Tables 4 and 5), at their best correction (Tables S5 and S6, Figures 2, 3, 4), showed the emergence of landscape‐specific scales and factors driving snow leopard distribution in our study areas. However, we observed important analogies related to snow leopard ecological requirements.

In both study areas, our results for CTI and SLP are consistent in predicting highest snow leopard occurrence probability in areas of the landscape on ridges and uplands away from large valley bottoms, but show landscape‐specific differences in how topography limits occurrence based on the grain and heterogeneity of the topographic structure of the landscape.

A striking similarity was observed with regards of the general composition of the landscape. Differences in the patch mosaic across areas might cause ecological associations being described by different metrics, as a consequence of the ecosystem complexity in the areas, which is more pronounced in the Himalayas due to larger altitude gradients (Bai et al., 2018). However, our results were consistent in revealing snow leopard preference for landscapes with high aggregation of a few dominant land cover class types (Figures 5 and 6), facilitating dispersal and ability to integrate large territorial home ranges (Johansson et al., 2018).

In both landscape, snow leopard habitat suitability was consistently associated with the extent of grassland patches, and the effect at which they locally influence occurrence is revealed by their different magnitudes (Figures 5 and 6). The steady selection toward optimal amounts of habitat types (Table 3) is confirmed by the identification of PLAND_Gr in both study area as a characteristic driver of snow leopard habitat suitability (Tables 4 and 5; Figures 5 and 6), which is consistent not only with respect to the identified scale of selection (Table 3) but most importantly for its magnitude (Figures 5 and 6). This suggests the key role of such landscape attribute in driving general occurrence for the species throughout its range.

The identification of PLAND_Gr and of GYR_AM_Gr as recurring landcover metrics in the top five models (Tables 4 and 5), across the two study areas, might be related to habitat choices in function of predatory behavior (Hayward, Hayward, Tambling, & Kerley, 2011; Lyngdoh et al., 2014). Grassland and sparse vegetation are important components of the landscape for wild ungulates, small mammals, and birds, as well as for livestock, on which snow leopards are known to occasionally prey (Bagchi & Mishra, 2006; Chen et al., 2016; Lyngdoh et al., 2014). Therefore, the selection of a landscape with a total optimum amount of sparse vegetation in a given radius (as highlighted by PLAND_Gr), regardless of its extensiveness or continuity (revealed by GYR_AM_Gr) might be indicative of habitat choices intended to maximize hunting opportunities by foraging in preys’ feeding grounds, and to balance trade‐offs in energy expenditures to locate and chase them (Hayward et al., 2011; Hayward, Jędrzejewski, & Jêdrzejewska, 2012; Lyngdoh et al., 2014).

The similar values of river density in the two areas might depend on general hydrological attributes characterizing mountainous environments across snow leopard range, but suggest domains of habitat characteristics (Wiens, 1989), as density is constrained within a similar range of values (Figures 5 and 6). These selection patterns might again be related to foraging behavior, as snow leopards are known to ambush their preys along ravines and river bluffs (Riordan, pers. comm.; Jackson & Ahlborn, 1984).

Annual average temperature represented the strongest determinant of habitat across the two study areas (Tables S5 and S6). However, it is not possible from this study to separate the effects of climate per se with those of the correlated topographic features. Lower values of mean annual temperature in QLSNP reflects the species’ association with higher elevation mountainous areas in the landscape, while the slightly higher values in QMLNR are indicative of the moderately rugged plateaus, relatively distant from the higher Himalayas mountains, in which the snow leopard presence points occur (Figures 5 and 6).

### 4.6 Effect of landscape‐specific limiting factors

Our study confirms observations from McGarigal and Cushman (2002) on the utility of meta‐replicated landscape‐level analyses in studies of species distribution, to uncover local habitat associations and provide generalizations based on species ecology.

Our results show a pattern of snow leopard habitat selection at a finer scale when those environmental predictors vary at fine scales across the landscape or are widely distributed through the landscape. Environmental factors that are not highly variable, or that vary at broad scales with low local variation within landscapes, tended to be selected at coarser scales. Although snow leopards have a consistent response to landscape topography and composition, the extent to which habitat components vary, in relation to local attributes, lead primarily to a differential scale of effect of such predictors, and secondly to the inclusion of different limiting factors (Cushman et al., 2013; Shirk et al., 2014; Short Bull et al., 2011) as strongest descriptors of habitat in different areas (Tables 3, 4, 5, Tables S3 and S4).

The effect of a predictor will be detected in a model only if it bears enough variability, such that it is able to differentially affect the modeled response (Reddy et al., 2019; Shirk et al., 2012, 2014; Short Bull et al., 2011; Vergara, Cushman, & Ruiz‐González, 2017). The effect of variables instead will not be identified in a model if they are too homogeneous or bear not enough variability. This does not imply they lack ecological importance, rather that they do not possess sufficient power to structure the response variable (Short Bull et al., 2011).

We observed a reversed pattern of selection for ELEV, dependent on its variability across the entire extents. Finer scales are thus associated with higher local elevation differences, especially notable considering the altitude of the high Himalayas compared to the plateaus with snow leopard occurrence, while a landscape with low local variation (as the Qilian Mountains) produced a coarse scale of response (Table 3).

In contrast to this, we highlight the role of the complexity of the mountain texture in determining scales of response for other derived metrics. The topographic homogeneity of uplands with snow leopards presence in QMLNR (although presenting landscape attributes locally favorable for their occurrence) causes less local landscape variation, and the major landscape topographic gradients are between extreme mountain peaks and plateaus, driving the selection of derived topographic descriptors at coarse scales (Table 3).

Although the best multivariate models in the two areas (Tables 4 and 5, Tables S3 and S4) agreed in the general preference for high elevation dry areas (Figures 5 and 6), it is interesting to observe how different aspects of topography emerge in different contexts as a consequence of what is locally limiting snow leopards occurrence. CTI then is the only metric in QMLNR able to frame the large hydrological gradients between upland and lowland conditions, and SLP in QLSNP is associated with a fine‐scale high degree of slope, consequence of the topographic texture of the area. These descriptors are the best factors in their respective study area possessing enough variability to achieve a higher discriminatory power.

Complexity of the patch mosaic in each area translates into different scales of selection for the most abundant landcover types. Smaller scales are indicative of a less complex landscape with wider and larger patches, driving the selection of area, extensiveness and contrast metrics at finer scales, representing thus landscape‐specific factors (Table 3). These landscape properties emerge in the multivariate context where we observe AI in QLSNP and CWED in QMLNR as best landscape‐level descriptors for the two areas (Tables 4 and 5, Tables S3 and S4).

In situations in which a landscape is composed of few main classes, with patches having low edge and large extent (as in QLNSP), occurrence patterns for snow leopard will be better described by a metric of aggregation. When the landscape mosaic is more heterogeneous, showing an alternation of patches with higher contrast (as in QMLNR), habitat suitability will be associated with metrics revealing density of, and contrast among, different land types, indicative of the presence, and possibly the avoidance, of nonoptimal and suboptimal habitats (Tables 4 and 5; Figures 5 and 6). This allows also the inclusion of more classes as best habitat discriminants (Tables 4 and 5, Table S4, Figure 6).

Optimal scales for hydrological and anthropogenic features were selected as a function of their relative abundance, with smaller scales indicative of more homogeneous patterns and fine‐scale variation (Table 3). Their performance in top multivariate models in the two study areas (Tables 4 and 5, Tables S3 and S4) again provides evidence of the importance of replication as a means to identify locally limiting factors (McGarigal & Cushman, 2002; Reddy et al., 2019; Shirk et al., 2012, 2014; Short Bull et al., 2011; Vergara et al., 2017).

Snow leopard occurrences are embedded in medium‐scale homogeneity conditions of riverine and settlements features in QLSNP and QMLNR, respectively. Therefore, as these factors are minimally variable, occurrence patterns on the whole landscapes are best explained by a negative association with human settlements and infrastructures in QLSNP (which are mostly concentrated around Qinghai Lake and on the northern foot of Qilian mountains), and by landscape‐level patterns of hydrological heterogeneity in QMLNR, which is consistent with the general topographic properties of the whole area, already described for CTI.

Summarizing, we found that natural history of the snow leopard might dictate a range of scales for several predictors. However generalizations on scales pertinent to the same species in replicated study areas should be made after careful evaluation, as each scale could be optimal within the landscape‐specific context in which it has been observed, given variation in limiting factors between different landscapes (Cushman et al., 2011; Shirk et al., 2014; Short Bull et al., 2011; Wan et al., 2017). This does not imply, however, that it is impossible to generalize habitat associations, given scales and variables related to local limiting factors in a landscape. Rather, as in the cases of Short Bull et al. (2011), Shirk et al. (2012), Vergara et al. (2017), and Reddy et al. (2019), it is possible to predict a priori which variables will be limiting occurrence in a given landscape, and also, to some extent, at which scales they will be most limiting, based on the structure and composition of the meta‐replicated landscapes (Tables 3, 4, 5, Tables S3 and S4). This is critical to understanding the habitat niche of a species and when different dimensions (variables) of that niche become limiting to its pattern of occurrence.

### 4.7 Performance of bias correction methods

Our bias correction framework confirmed the impact of sampling bias in distribution modeling (Figures 2 and 3), as the impacts of the radius of correction and correction type (Table 6, Appendix S4). Consistent with previous examples (e.g., Vergara et al., 2015), our models confirm that Gaussian kernels are usually the best method of bias correction and almost always superior to spatial rarefaction of occurrences. Our results also show that evaluating model performance based soley on the magnitude of AUC is questionable, especially when evaluating the effectiveness of correction types, as AUC values are strictly a function of how the occurrences are distributed in space and of sample size, with rarefied occurrences leading to a decrease of this metric regardless of model performance (Chapman, 2010; Fourcade et al., 2014; Hijmans, 2012; Jimenez‐Valverde, 2012; Lobo, Jiménez‐Valverde, & Real, 2008; Merckx, Steyaert, Vanreusel, Vincx, & Vanaverbeke, 2011; Phillips et al., 2006; Veloz, 2009).

Many previous studies advocated the use of omission rates to evaluate SDMs performances (Kramer‐Schadt et al., 2013; Liu et al., 2013; Shcheglovitova & Anderson, 2013; Boria et al., 2014; Radosavljevic and Anderson, 2014; Vergara et al., 2015). We note the robustness of GKs as an effective way to improve the average omission rate with respect to the average of the raw models, particularly for the RD datasets (strong bias conditions), but in general, the application of a sampling intensity mask improves model performance under all scenarios we evaluated (Appendix S4). Given these results, we recommend that studies using PO modeling assess the effect of different radii of GKs to improve model performance based upon reduction of omission rate, and increasing predictive overlap witha reference simulated scenario (Table 6, Appendix S4). SRs is only advantageous when occurrences are highly dispersed over large extents (Appendix S4).

We found our model selection framework, based on ensembling and averaging competing models, to be an effective strategy to infer relative occurrence probability, while minimizing omission of the effect of different variables belonging to different competing models (Figures 2, 3, 4; Table 6, Appendix S4). Assessing how bias correction improves overlap with a reference model is a useful strategy to account for the inherent uncertainty in species distributions, avoiding at the same time omission or commission biases (Mateo‐Sánchez et al., 2013; Shirk et al., 2014; Wasserman, Cushman, Shirk, et al., 2012) (Figures 2 and 3, Table 6). This is achievable by balancing simultaneously correction radii, improvement in overlap, and omission rate (Figures 2 and 3, Table 6, Appendix S4).

There are caveats in the use of Schoener's *D* (Schoener, 1968) and Δ*D*_geo_ (Fourcade et al., 2014) alone as a proxy for successful correction. We also warrant against the use of MTSS omission rate alone, the reasons for which are expounded on in Appendix S4, together with additional considerations on our modeling approach.

## 5 CONCLUSIONS

This study emphasized the key influences of scale dependency on the identification of optimal relationships between the occurrence of a focal species and environmental gradients, offering further evidence for the need to integrate scale selection into species distribution and habitat suitability modeling (McGarigal et al., 2016). If scales of effect are not optimized, this may lead to misinterpretation of ecological determinants, resulting in suboptimal management strategies based upon ecologically unrealistic distribution models (Bellamy et al., 2013; Mateo‐Sánchez et al., 2013; Shirk et al., 2014; Timm et al., 2016; Vergara et al., 2015; Wan et al., 2017; Wasserman, Cushman, Wallin, et al., 2012) (Appendix S3).

We predicted the suitable habitat for snow leopard in two landscapes of western China, which differ in human footprint, hydrological complexity, topographic features, landcover attributes and climatic conditions We tested the scale‐dependent response to environmental predictors, revealing its nonstationarity across many metrics. Such scale responses are landscape‐specific, mostly related to topographic complexity, the configuration of the patch mosaic, anthropic presence, and hydrological network. Snow leopards respond to landscapes at mostly broad scales, in line with their home range requirements and the low productivity of cold and xeric environments. Many such scales suggest domains of response (sensu Wiens, 1989), especially with regard of metrics describing landscape composition and relative proportion of classes.

Multivariate models revealed the way in which different limiting factors emerge in different contexts, driven by local variability of environmental conditions (Cushman et al., 2011; Levin, 1992; Mayor, Schneider, Schaefer, & Mahoney, 2009; Shirk et al., 2012). We observed area‐specific differences in the relative importance of human footprint and hydrological network, different limiting landscape‐level attributes and a differential influence of topographic metrics in describing snow leopard habitat in the two areas.

Accounting for these differences, we have been able to generalize ecological associations based on the interpretation of response curves from the top models. Our results identified consistent fine‐scale association with temperature, medium/broad‐scale associationwith sparse vegetation on ridges and uplands, and broad‐scale association with aggregated and low‐contrast landscapes. The consistent values of relative abundance of sparse vegetation and thresholds of watercourse density, possibly related to predatory behavior, were the strongest limiting factors related to snow leopard ecology, implying ecological domains related to the magnitude of those attributes. The consistently strong relationship to temperature highlights the climatic sensitivity to snow leopard and strongly suggests additional work evaluating the effects of climate change on its habitat suitability and population connectivity (e.g., Wasserman, Cushman, Littell, Shirk, & Landguth, 2013; Wasserman, Cushman, Shirk, et al., 2012).

In our simulations, multi‐scale models outperformed single‐scale counterparts in most cases. Even when single‐scale models perform equivalently, identifying the best single‐scale requires scale optimization (McGarigal et al., 2016). Therefore, we suggest as a general approach employing a multi‐scale modeling strategy where each covariate is allowed to vary independently in an analysis that optimizes their individual scales.

This study provides guidelines for selection of model optimization strategies across a range of different occurrence configurations reflecting bias intensities (Appendix S4). We presented a framework in which we coupled a restricted background with spatial filtering or Gaussian density kernels and created reference models directly related to an ensemble of the best raw suitability surfaces. We ensembled competing models in their raw and corrected versions to capture a whole range of potential suitability scenarios in a single output and evaluated their performances based on average metrics. This approach yielded a description of habitat which framed the effect of several different predictors, assessed without interactions with variables describing the same underlying landscape characteristic (i.e., topography). This ensembling strategy, when coupled with maximization of overlap with respect to a hypothetical reference model (Fourcade et al., 2014; Veloz, 2009), allowed us to select the best average correction that further minimized the MTSS omission rate, which is desirable for model accuracy (Liu et al., 2013; Boria et al., 2014, Radosavljevic & Anderson, 2014; Vergara et al., 2015).

We found that Gaussian density kernels are the best optimization for heavily clustered occurrences, but perform well under each bias circumstance. Spatial rarefaction on average is effective when the presence points are more uniformly distributed in space (Appendix S4). While accounting for overfitting, we deem desirable selecting a threshold to minimize omission errors, and to create a reference model which is probabilistically related (but not as biased) to the real data, upon which a metric of niche overlap (and its improvement after correction) must be selected.

We invite researchers and practitioners to carefully consider model‐building strategies, optimizing scales of effect, accounting for sample bias in PO datasets and employing simulation approaches to evaluate modeling methods and bias correction. Both the choice of an arbitrary scale, the inclusion of arbitrarily chosen predictors (among a wider range of possible explanatory variables), and the choice of an arbitrary type and radius of correction, might result in incorrect conclusions, if different scenarios are not tested.

## CONFLICT OF INTEREST

The Authors declare no competing interests.

## AUTHOR CONTRIBUTIONS

**Luciano Atzeni:** Conceptualization (equal); Data curation (lead); Formal analysis (lead); Investigation (lead); Methodology (equal); Project administration (supporting); Supervision (equal); Visualization (lead); Writing‐original draft (lead); Writing‐review & editing (lead). **Samuel A. Cushman:** Conceptualization (lead); Formal analysis (equal); Investigation (lead); Methodology (lead); Supervision (lead); Writing‐original draft (equal); Writing‐review & editing (equal). **Defeng Bai:** Data curation (equal); Formal analysis (supporting); Investigation (supporting); Methodology (supporting); Writing‐original draft (equal); Writing‐review & editing (equal). **Jun Wang:** Data curation (equal); Formal analysis (supporting); Investigation (supporting); Methodology (supporting); Writing‐original draft (supporting); Writing‐review & editing (supporting). **Pengju Chen:** Data curation (equal); Writing‐original draft (supporting); Writing‐review & editing (supporting). **Kun Shi:** Conceptualization (equal); Data curation (supporting); Formal analysis (supporting); Funding acquisition (lead); Investigation (supporting); Methodology (supporting); Project administration (lead); Supervision (lead); Writing‐original draft (equal); Writing‐review & editing (equal). **Philip Riordan:** Conceptualization (equal); Formal analysis (equal); Investigation (equal); Methodology (supporting); Supervision (equal); Writing‐original draft (equal); Writing‐review & editing (equal).

## Supporting information

Appendix S1

Appendix S2

Appendix S3

Appendix S4

Tables S1–S6

Supplementary Materials

Supplementary Materials

## Data Availability

Datasets of occurrences in both study areas cannot be made available online before completion of National Forestry and Grassland Administration of China (NFGA) project. Specific requirements can be addressed directly to the corresponding author by interested third parties.
