# Supplementary material for: Meta‐replication, sampling bias, and multi‐scale model selection: A case study on snow leopard (Panthera uncia) in western China
Source: Ecol Evol. 2020 Jul 6;10(14):7686–712. doi: 10.1002/ece3.6492 (PMC7391562; doi:10.1002/ece3.6492)
Supplement: Supplementary file 1 — Appendix S1 [file ECE3-10-7686-s001.docx]

**Appendix 1**

**List of topographical variables adopted in this study**

**Dissection:** Describes dissection in an elevation surface. It represents a modified version of de Martonne’s parameter of terrain massiveness – coefficient of dissection (Pike and Wilson, 1971; Evans, 1972):

; where Z stands for elevation

**Slope Position:** Also known as Topographic Position Index (TPI) (Weiss, 2001; De Reu et al., 2013), it measures the difference in elevation between the central cell of a neighborhood of radius *R* and the mean of elevation in the same neighborhood.

; where Z stands for elevation

Quoted from De Reu et al., (2013), p. 42

“*Positive TPI values indicate that the central point is located higher than its average surroundings, while negative values indicate a position lower than the average. The range of TPI depends not only on elevation differences but also on R (e.g. Grohmann and Riccomini, 2009). Large R-values mainly reveal major landscape units, while smaller values highlight smaller features, such as minor valleys and ridge*s”.

**Compound Topographic Index:** It is referred to as a steady state wetness index to quantify catenary landscape position.

**;

where *A_s_* expresses the catchment area (in m^2^) per unit width orthogonal to the flow direction, and *β* is the slope in radians (Gessler et al., 1995). CTI measures the degree to which surface flow across the landscape is funnelled through each pixel (Gessler et al., 1995; Mukherjee et al., 2012). It indicates the accumulated water flow at any point in a catchment, with higher values reached in valley bottoms, where soil has more potential to be saturated (Mukherjee et al., 2012).

**Roughness:** The Topographic Ruggedness Index (TRI), developed by Riley et al. (1999), expresses the amount of difference in elevation between adjacent cells in a given neighborhood. After calculating all the differences in a neighborhood, these are squared to make them positive, and averaged. A square root is extracted from this value, expressing the average elevation change between any point in that neighborhood and the surroundings cells within the same neighborhood (Evans et al., 2014).

**References**

De Reu, J., J. Bourgeois, M. Bats, A. Zwertvaegher, et al. (2013) Application of the topographic position index to heterogeneous landscapes. Geomorphology 186, 39-49.

Evans J.S., Oakleaf J., Cushman S.A., Theobald D. (2014) An ArcGIS Toolbox for Surface Gradient and Geomorphometric Modeling, version 2.0-0.

Evans, I. S., 1972. General geomorphometry, derivatives of altitude, and descriptive statistics. In Chorley, R. J., Spatial Analysis in Geomorphology New York: Harper & Row pp.17 – 90.

Gessler, P.E., I.D. Moore, N.J. McKenzie, and P.J. Ryan. (1995). Soil-landscape modeling and spatial prediction of soil attributes. *International Journal of GIS*. 9(4), 421-432.

Grohmann, C.H., Riccomini, C., 2009. Comparison of roving-window and search-window techniques for characterising landscape morphometry. Computers & Geosciences 35, 2164–2169.

Mukherjee S., Mukherjee S, Garg R., et al. (2013) Evaluation of topographic index in relation to terrain roughness and DEM grid spacing. J Earth Syst Sci 122:869–886. doi: 10.1007/s12040-013-0292-0

Pike, R.J., Wilson, S.E., 1971. Elevation relief ratio, hypsometric integral, and geomorphic area altitude analysis. Bull. Geol. Soc. Am. 82, 1079-1084.

Riley, S. J., S. D. DeGloria and R. Elliot (1999). A terrain ruggedness index that quantifies topographic heterogeneity. Intermountain Journal of Sciences. 5, 1-4

Weiss, A.D., 2001. Topographic position and landforms analysis. Poster Presentation, ESRI Users Conference, San Diego, CA.
