# Supplementary material for: Meta‐replication, sampling bias, and multi‐scale model selection: A case study on snow leopard (Panthera uncia) in western China
Source: Ecol Evol. 2020 Jul 6;10(14):7686–712. doi: 10.1002/ece3.6492 (PMC7391562; doi:10.1002/ece3.6492)
Supplement: Supplementary file 2 — Appendix S2 [file ECE3-10-7686-s002.docx]

**Appendix 2**

**List of land-cover derived metrics**

**Class-level metrics**

**Percentage of Landscape (PLAND):** A measure of relative landscape composition, useful to compare landscapes of different size. PLAND equals the percentage of the landscape comprised of the corresponding patch type, and ranges from 0 to 100. PLAND equals 100 when the landscape is composed only of a single patch; conversely, it approaches 0 as a patch type becomes increasingly rarer.

**Area average weighted mean (AREA_AM):** A landscape-centric configuration metric. It expresses the average condition that the focal species would experience if dropped at random on the landscape. It describes the area-weighted mean patch size of patches of the corresponding class, where the proportional area of each patch is based on the total class area. At the class level, it equals the sum of all proportional areas of the class. It is expressed in hectares, without limits.

**Radius of Gyration (Area weighted mean) (GYR_AM):** A configuration metrics expressed in meters without limits. Also known as Correlation Length, it describes a measure of landscape continuity. In other words, it represents the average distance a focal species can move in any direction from a random starting point without leaving the initial patch. It increases as the shape of the patch becomes more elongated. At the class level, this metric is averaged across all patches of the corresponding type.

**Landscape-level metrics**

**Patch Density (PD):** It expresses the number of patches present in the landscape per 100 hectares. It is calculated dividing the number of patches by the total area of the landscape in m^2^, multiplied by 10000•100 to convert to 100 hectares. It facilitates the comparison among landscapes of different size.

**Aggregation Index (AI):** A landscape composition metric expressing degree of aggregation of patches across the landscape, based on the number of like adjacencies of the corresponding class and proportional to the proportion of the landscape comprised of that class. AI equals 0 when all patches are disaggregated, and equals 100 when the landscape is composed by a single patch. At landscape level, each class is weighted by its proportional area and scaled to account for all the possible like adjacencies. Refer to Fragstats Manual for more details.

**Constrast-weighted edge index (CWED):** This metric, expressed in meters per hectare, standardizes edge to a per-unit area basis, allowing the metric to be used to compare different landscape. It is calculated by summing all the meters of edge multiplied by their contrast weights, further multiplied by 10000 to convert to hectares. CWED is 0 when the landscape consists of a single patch type (no edge present).

For more details on calculation of all metrics, refer to:

McGarigal, K., S.A. Cushman, and E. Ene. (2012). FRAGSTATS v4: Spatial Pattern Analysis Program for Categorical and Continuous Maps. Computer software program produced by the authors at the University of Massachusetts, Amherst. Available at the following web site: http://www.umass.edu/landeco/research/fragstats/fragstats.html
