# Supplementary material for: Meta‐replication, sampling bias, and multi‐scale model selection: A case study on snow leopard (Panthera uncia) in western China
Source: Ecol Evol. 2020 Jul 6;10(14):7686–712. doi: 10.1002/ece3.6492 (PMC7391562; doi:10.1002/ece3.6492)
Supplement: Supplementary file 3 — Appendix S3 [file ECE3-10-7686-s003.docx]

**Appendix 3**

**Variable percent contribution across scales**

Variables contribution in multi-scale scenarios varied across models and study areas. For brevity we discuss only percent contribution. In QLSNP, SLP (range: 49.6-55.2) and TEMP (28.1-34.4) yielded the highest contribution, followed by landscape level (5.1-6.6) and class level (2.2-7) metrics, with density of features ranking last (1.5-10.1) (Appendix 3, Table 1). In QMLNR, variables in multi-scale models showed on average a more balanced contribution, with TEMP always contributing substantially (31-40.7), followed by landscape class-level metrics (8.3-37.2), CTI (10.3-26.9), landscape-level variables (8.1-21.8), and DENS_riv (2.4-4.4) (Appendix 3, Table 2).

All predictors exhibited different trends in the two study areas across scales. In QLSNP, SLP increases its contribution until 4800-9600, and steadily decreases at larger scales. TEMP contribution generally decreases up to 9600, then increases slightly at the two following scales. Its contribution for the largest scale is model-dependent, either following an increasing or decreasing trend. AI contribution steadily increases as the scales become larger. . DENS_rd and DENS_set start to acquire influence on the models from 4800 meters onwards, and their contribution is negligible at smaller scales. On the opposite, DENS_riv gives more contribution at the three smallest scales and from 9600 and beyond. Class-level metrics appear to be dependent on associated descriptors and a stable trend cannot be inferred (Appendix 3, Table 1).

In QMLNR, CTI contribution increases with increasing radius, reaching the highest values from 9600 to 19200 meters. TEMP steadily loses importance as the scale augments. The contribution of DENS_riv increases up to 9600 meters, then decreases at 14400 meters, and increases again at the largest radius or the two largest radii. CWED and PD achieve the highest contribution at the three largest scales. Class-level metrics show non-uniform trend as they performances are dependent on associated predictors, but overall, they tend to exhibit the highest importance at large scales from 9600 meters and beyond (Appendix 3, Table 2).

The differences in variables contribution across scales, highlight the need for a multiple scales optimization, that should represent the principal focus of any modelling approach (McGarigal et al., 2016). Our observations, in line with previous examples (Wasserman et al., 2012; Mateo-Sánchez et al., 2013; Timm et al., 2016; Vergara et al. 2015; Wan et al., 2017), suggest that non-optimizing the scale of predictors produces incorrect species-environment relationships, which may thus lead to an incorrect inference of the species’ ecological and habitat requirements (Mayor et al. 2009; McGarigal et al., 2016).

**References**

Mateo-Sánchez M, Cushman S, Saura S (2013) Scale dependence in habitat selection: the case of the endangered brown bear (Ursus arctos) in the Cantabrian Range (NW Spain). Int J Geogr Inf Sci 28:1531–1546. doi: 10.1080/13658816.2013.776684

Mayor S, Schneider D, Schaefer J, Mahoney S (2009) Habitat Selection at Multiple Scales. Ecoscience 20:238–247. doi: 10.2980/16-2-3238

McGarigal K, Wan H, Zeller KA, et al (2016) Multi-scale habitat selection modeling: a review and outlook. Landscape Ecology 31:1161–1175. doi: 10.1007/s10980-016-0374-x

Timm B, McGarigal K, Cushman S, Ecology GJ (2016) Multi-scale Mexican spotted owl (Strix occidentalis lucida) nest/roost habitat selection in Arizona and a comparison with single-scale modeling results.

Vergara M, Cushman S, Urra F, Ruiz-González A (2015) Shaken but not stirred: multiscale habitat suitability modeling of sympatric marten species (Martes martes and Martes foina) in the northern Iberian Peninsula. Landscape Ecol 31:1241–1260. doi: 10.1007/s10980-015-0307-0

Wan H, McGarigal K, Ganey JL, et al (2017) Meta-replication reveals nonstationarity in multi-scale habitat selection of Mexican Spotted Owl. The Condor 119:641–658. doi: 10.1650/CONDOR-17-32.1

Wasserman TN, Cushman SA, Wallin DO, Hayden J (2012) Multi scale habitat relationships of Martes americana in northern Idaho, U.S.A. USDA Forest Service RMRS Research Paper RMRS-RP-94.

|  |  | Multiscale | | 300 | | 600 | | 1200 | | 2400 | | 4800 | | 9600 | | 14400 | | 19200 | | 28800 | |
| --- | --- | --- | --- | --- | --- | --- | --- | --- | --- | --- | --- | --- | --- | --- | --- | --- | --- | --- | --- | --- | --- |
| *Model* | *Variables* | *Percent* | *Permutation* | *Percent* | *Permutation* | *Percent* | *Permutation* | *Percent* | *Permutation* | *Percent* | *Permutation* | *Percent* | *Permutation* | *Percent* | *Permutation* | *Percent* | *Permutation* | *Percent* | *Permutation* | *Percent* | *Permutation* |
| QLSNP_1 | DENS_set | **8** | **5,3** | 0 | 0 | 0 | 0 | 0 | 0 | 0,1 | 0 | 1,4 | 0,8 | 2,9 | 0,9 | 4,2 | 2,5 | 6,3 | 6,2 | 13,5 | 13,4 |
|  | SLP | **51,1** | **33,7** | 50,6 | 32 | 54,6 | 35,3 | 52,1 | 35,3 | 53,5 | 35,2 | 53,9 | 40,7 | 53,1 | 41,6 | 43,9 | 32,9 | 32,2 | 23,7 | 30,1 | 22,3 |
|  | TEMP | **28,1** | **46,4** | 45,1 | 64 | 39,2 | 60,7 | 35,1 | 54,4 | 32,8 | 51,4 | 31,2 | 46,7 | 28,3 | 47,2 | 32,1 | 43,5 | 33,6 | 38,9 | 25 | 29,4 |
|  | AI | **5,8** | **6,7** | 3,8 | 3,7 | 1,9 | 1,5 | 5,1 | 4,1 | 8,3 | 5,6 | 8,6 | 5,8 | 10,8 | 6,4 | 13,6 | 12 | 19,9 | 17,6 | 21,3 | 23 |
|  | GYR_AM_Gr | **7** | **7,9** | 0,4 | 0,3 | 4,3 | 2,5 | 7,7 | 6,2 | 5,2 | 7,9 | 4,9 | 6 | 4,9 | 3,9 | 6,2 | 9 | 6,3 | 6,2 | 10,1 | 11,9 |
| QLSNP_2 | DENS_set | **7,1** | **5,3** | 0 | 0 | 0 | 0 | 0 | 0 | 0,2 | 0 | 1,4 | 1 | 3,1 | 1,4 | 4,4 | 2,8 | 6,1 | 5,9 | 15 | 9,8 |
|  | SLP | **53,7** | **31,7** | 50,4 | 31,9 | 53,5 | 34,7 | 52,9 | 34,7 | 53,6 | 35,7 | 55,8 | 40,8 | 56,9 | 46,2 | 48 | 44,6 | 38,6 | 31,9 | 34,2 | 27,6 |
|  | TEMP | **31,6** | **53,6** | 45,5 | 64,2 | 41,4 | 62 | 38,4 | 58,7 | 37,3 | 55,7 | 34,6 | 51,5 | 29,1 | 48,1 | 31,7 | 45,9 | 36,6 | 46,4 | 27,8 | 34,4 |
|  | AI | **5,4** | **8,8** | 3,9 | 3,8 | 2,1 | 1,3 | 4,7 | 3,3 | 5,6 | 4,7 | 7,5 | 6,2 | 10,9 | 4,3 | 13,3 | 3,8 | 16,8 | 13,4 | 18,9 | 22,3 |
|  | PLAND_Gr | **2,2** | **0,6** | 0,2 | 0,1 | 3 | 1,9 | 4 | 3,3 | 3,3 | 3,9 | 0,7 | 1 | 0 | 0 | 2,5 | 2,9 | 2 | 2,5 | 4 | 6 |
| QLSNP_4 | DENS_riv | **1,5** | **1** | 8,5 | 4,4 | 10 | 4,6 | 6,2 | 1,2 | 0,3 | 0 | 1,4 | 3,2 | 11,3 | 12,7 | 21,3 | 16,8 | 20 | 17,1 | 6,1 | 5,9 |
|  | SLP | **55,2** | **32,5** | 50,3 | 33,9 | 52,9 | 36,8 | 51,3 | 35,5 | 58 | 37,8 | 62,7 | 43,2 | 56,9 | 47,5 | 43,7 | 42,5 | 36,9 | 35,9 | 36,8 | 31,9 |
|  | TEMP | **34,4** | **54,6** | 38 | 58,8 | 31,7 | 55,9 | 33,6 | 56,2 | 32 | 50,4 | 28,4 | 45,9 | 19,8 | 35,5 | 22,4 | 35,5 | 27,2 | 37,9 | 33,8 | 38,7 |
|  | AI | **6,6** | **10,6** | 1,9 | 2,4 | 0,8 | 1,1 | 4,3 | 4,7 | 6,3 | 8 | 7,2 | 6,5 | 9 | 1,9 | 11,6 | 5,1 | 15,6 | 9,1 | 21,6 | 23,1 |
|  | PLAND_Gr | **2,2** | **1,3** | 1,3 | 0,5 | 4,7 | 1,5 | 4,5 | 2,4 | 3,3 | 3,8 | 0,3 | 1,2 | 3 | 2,4 | 0,9 | 0,1 | 0,4 | 0 | 1,7 | 0,5 |
| QLSNP_6 | DENS_rd | **9,6** | **8,8** | 0 | 0 | 0 | 0 | 0 | 0 | 0,2 | 0,3 | 1,9 | 2,3 | 8,7 | 6,5 | 12,2 | 11,4 | 18 | 19 | 13,4 | 10,6 |
|  | SLP | **50,2** | **27,7** | 50,4 | 31,9 | 53,5 | 34,7 | 52,9 | 34,7 | 53,5 | 35,6 | 55,1 | 39,3 | 53,8 | 42,8 | 46,4 | 41,8 | 36,4 | 30,3 | 32,8 | 26 |
|  | TEMP | **31,9** | **51,5** | 45,5 | 64,2 | 41,4 | 62 | 38,4 | 58,7 | 37,5 | 55,6 | 34,9 | 51,8 | 29,3 | 47,3 | 30,4 | 43,7 | 32,7 | 43,4 | 31,4 | 37,9 |
|  | AI | **5,9** | **8,8** | 3,9 | 3,8 | 2,1 | 1,3 | 4,7 | 3,3 | 5,6 | 4,6 | 7,3 | 5,7 | 8,2 | 3,3 | 9 | 1,5 | 11,9 | 6,5 | 19,4 | 23,2 |
|  | PLAND_Gr | **2,4** | **3,1** | 0,2 | 0,1 | 3 | 1,9 | 4 | 3,3 | 3,3 | 3,9 | 0,7 | 0,9 | 0 | 0 | 2 | 1,6 | 1 | 0,8 | 2,9 | 2,3 |
| QLSNP_7 | DENS_rd | **10,1** | **8** | 0 | 0 | 0 | 0 | 0 | 0 | 0,2 | 0,7 | 1,9 | 3,1 | 8,1 | 6,5 | 11,5 | 9,5 | 14,6 | 11,4 | 11 | 10,5 |
|  | SLP | **49,6** | **31,4** | 50,6 | 32 | 54,7 | 35,3 | 52,1 | 35,3 | 53,4 | 35 | 53,1 | 39,6 | 50,1 | 38,7 | 41,9 | 31,8 | 30,4 | 25,2 | 29,8 | 21 |
|  | TEMP | **29,2** | **46,9** | 45,1 | 64 | 39,1 | 60,7 | 35,1 | 54,4 | 33 | 50,9 | 31,6 | 46,7 | 28,5 | 46 | 31,1 | 43 | 32,7 | 41,8 | 26,9 | 33,8 |
|  | AI | **5,1** | **7** | 3,8 | 3,7 | 2 | 1,4 | 5,1 | 4,1 | 8,1 | 5,5 | 8 | 5 | 8,2 | 5,1 | 9,5 | 7,7 | 14,6 | 11,5 | 23,6 | 26,1 |
|  | GYR_AM_Gr | **5,9** | **6,6** | 0,4 | 0,3 | 4,1 | 2,5 | 7,7 | 6,2 | 5,3 | 7,9 | 5,4 | 5,7 | 5,1 | 3,8 | 6 | 8 | 7,7 | 10,2 | 8,8 | 8,6 |

**Appendix 3, Table 1. Variables percent contribution and permutation importance across the top 5 performing multi-scale and unscaled models in Qilianshan National Park. In bold, variables contribution of the models selected after the scale comparison step.**

|  |  | Multiscale | | 300 | | 600 | | 1200 | | 2400 | | 4800 | | 9600 | | 14400 | | 19200 | | 28800 | |
| --- | --- | --- | --- | --- | --- | --- | --- | --- | --- | --- | --- | --- | --- | --- | --- | --- | --- | --- | --- | --- | --- |
| *Model* | *Variables* | *Percent* | *Permutation* | *Percent* | *Permutation* | *Percent* | *Permutation* | *Percent* | *Permutation* | *Percent* | *Permutation* | *Percent* | *Permutation* | *Percent* | *Permutation* | *Percent* | *Permutation* | *Percent* | *Permutation* | *Percent* | *Permutation* |
| QMLNR_1 | Dens_riv | **2,8** | **1,8** | 1,6 | 0,1 | 2,7 | 0,2 | 4,2 | 0,6 | 5,9 | 0,2 | 12,6 | 2,2 | 15,9 | 3,1 | 7,9 | 7,2 | 6 | 7,8 | 18,2 | 17,3 |
|  | CTI | **25,3** | **28,1** | 8,7 | 7,9 | 14,2 | 13,2 | 18,6 | 19,1 | 26,3 | 22,8 | 31,4 | 32,4 | 27,5 | 42,5 | 27,5 | 40,7 | 27,9 | 32,5 | 11,3 | 21,4 |
|  | TEMP | **35,4** | **37,5** | 72,9 | 79,3 | 66,5 | 74,8 | 57,2 | 67,5 | 57,2 | 64,2 | 49,7 | 60,7 | 22,6 | 16,8 | 16,9 | 12,3 | 13,1 | 17 | 23,4 | 22,2 |
|  | CWED | **12,8** | **19,5** | 8,7 | 7,9 | 5 | 5,3 | 5,5 | 3,5 | 5,6 | 4,8 | 6,1 | 3,7 | 8,6 | 9,3 | 14,1 | 20,5 | 32,2 | 33,4 | 19,9 | 31,7 |
|  | PLAND_Gr | **23,7** | **13,1** | 11,7 | 8,1 | 11,5 | 6,5 | 14,6 | 9,4 | 4,9 | 7,9 | 0,2 | 1,1 | 25,4 | 28,2 | 33,6 | 19,4 | 20,8 | 9,3 | 27,3 | 7,3 |
| QMLNR_2 | Dens_riv | **2,4** | **1,1** | 1 | 0,3 | 2,7 | 0,2 | 3,7 | 0,6 | 5,9 | 0,5 | 12,1 | 1 | 12,5 | 7,8 | 6,8 | 11,2 | 9,6 | 25,6 | 24,8 | 23,2 |
|  | CTI | **18** | **18,6** | 7,7 | 9,7 | 13 | 11,1 | 19,4 | 21,6 | 26,2 | 22,4 | 28 | 28,1 | 25 | 32,9 | 22,4 | 36,3 | 25,5 | 32 | 12,4 | 9,6 |
|  | TEMP | **34,7** | **46,8** | 73,8 | 75,3 | 66,9 | 76,8 | 59,6 | 66 | 59,1 | 65,2 | 49,9 | 61,5 | 27,2 | 33 | 18,5 | 9,6 | 8,3 | 11,1 | 12,4 | 15,1 |
|  | CWED | **10,5** | **23,4** | 5,3 | 7,2 | 5,2 | 4 | 4,7 | 3,3 | 4,9 | 4,9 | 4,1 | 4,4 | 13,7 | 8,3 | 24,8 | 31,7 | 20,9 | 24,3 | 15,1 | 38,8 |
|  | GYR_AM_ Gr | **34,4** | **10,1** | 12,3 | 7,5 | 12,2 | 7,9 | 12,7 | 8,5 | 3,9 | 7 | 6 | 5 | 21,5 | 18 | 27,5 | 11,3 | 35,7 | 7 | 35,3 | 13,2 |
| QMLNR_4 | Dens_riv | **4,4** | **0,5** | 1,3 | 0,2 | 2,6 | 0,4 | 3,9 | 0,5 | 4,2 | 0,5 | 6,4 | 0 | 9,6 | 4,1 | 5,7 | 11,1 | 2,6 | 8,9 | 2,9 | 4,7 |
|  | CTI | **10,3** | **22,7** | 11,9 | 14,5 | 20,7 | 21,8 | 28,8 | 22,1 | 36,4 | 29,8 | 40 | 37,1 | 39,3 | 44,5 | 30,4 | 35,4 | 19,3 | 18,6 | 7,5 | 11,9 |
|  | TEMP | **31** | **37,3** | 67,8 | 78,9 | 59,5 | 68,8 | 49,8 | 66,3 | 39,5 | 52,2 | 30,3 | 129,4 | 21 | 21,4 | 18,5 | 10,1 | 15,1 | 15,4 | 11 | 17,5 |
|  | CWED | **17,1** | **16,8** | 4,1 | 3,6 | 2,5 | 3,5 | 1,9 | 2 | 1,1 | 0,7 | 0,8 | 0,6 | 5,3 | 4,1 | 22,2 | 26,2 | 31,8 | 35,6 | 28,8 | 32,7 |
|  | GYR_AM_Br | **37,2** | **22,7** | 14,8 | 2,9 | 14,7 | 5,6 | 15,6 | 9 | 18,8 | 16,7 | 22,5 | 32,9 | 24,8 | 27,2 | 23,2 | 17,2 | 31,2 | 21,5 | 49,8 | 33,2 |
| QMLNR_5 | Dens_riv | **2,9** | **3,7** | 1,5 | 0,1 | 2,5 | 0,3 | 3,3 | 0,3 | 4 | 0,1 | 9,4 | 0 | 15,7 | 5,2 | 8,6 | 12,3 | 12,3 | 25,7 | 23,4 | 23 |
|  | CTI | **26,9** | **22** | 11,2 | 10,7 | 17,6 | 17,9 | 24,6 | 20,9 | 30,4 | 25,8 | 31,4 | 31,3 | 32,9 | 43 | 34,2 | 43 | 39 | 29,4 | 18,5 | 12,4 |
|  | TEMP | **40,4** | **40,1** | 77,9 | 79,6 | 71,4 | 73,5 | 63,9 | 67,9 | 54,8 | 58,2 | 45,5 | 48,9 | 26,9 | 23,7 | 19 | 7,8 | 18,1 | 12,1 | 17,4 | 20,4 |
|  | CWED | **8,1** | **20** | 3,5 | 2,9 | 2,2 | 0,6 | 0,9 | 0,4 | 0,8 | 0,6 | 0,7 | 0,6 | 6,3 | 4,7 | 12,5 | 13,5 | 13 | 26,9 | 8,9 | 30,7 |
|  | GYR_AM_NLF | **21,8** | **14,2** | 6 | 6,7 | 6,2 | 7,7 | 7,3 | 10,6 | 10 | 15,2 | 12,9 | 19,1 | 18,3 | 23,3 | 25,8 | 23,3 | 17,6 | 5,9 | 31,8 | 13,4 |
| QMLNR_6 | Dens_riv | **3,5** | **4,1** | 1,2 | 0,2 | 2,7 | 0,4 | 4,4 | 0,8 | 5,6 | 0,2 | 12,5 | 0,7 | 14,9 | 4,7 | 8,6 | 6,9 | 5,9 | 12,1 | 17,9 | 12,1 |
|  | CTI | **25,6** | **24,7** | 7,5 | 7,5 | 13,2 | 11,9 | 17,6 | 20,2 | 25 | 20,2 | 29,7 | 29,6 | 29 | 32,4 | 31,1 | 32,9 | 43,5 | 27 | 17,4 | 18,8 |
|  | TEMP | **40,7** | **43,9** | 75,9 | 76,1 | 69,8 | 80 | 59,3 | 66,9 | 56,1 | 63 | 48,9 | 60,4 | 25,2 | 28,7 | 21,9 | 19,1 | 20,9 | 21,9 | 18 | 26,9 |
|  | PD_19200 | **21,8** | **15,6** | 3,7 | 3,5 | 2,8 | 1,7 | 3,3 | 2,5 | 8,1 | 7 | 8,8 | 7,4 | 14,1 | 16,3 | 24,6 | 26,6 | 20,9 | 30,8 | 32,2 | 37 |
|  | PLAND_Gr | **8,3** | **11,8** | 11,7 | 12,7 | 11,5 | 5,9 | 15,4 | 9,5 | 5,3 | 9,6 | 0,1 | 1,9 | 16,8 | 17,8 | 13,9 | 14,5 | 8,8 | 8,1 | 14,4 | 5,3 |

**Appendix 3, Table 2. Variables percent contribution and permutation importance across the top 5 performing multi-scale and unscaled models in Qomolangma National Nature Reserve. In bold, variables contribution of the models selected after the scale comparison step.**
