# Supplementary material for: Meta‐replication, sampling bias, and multi‐scale model selection: A case study on snow leopard (Panthera uncia) in western China
Source: Ecol Evol. 2020 Jul 6;10(14):7686–712. doi: 10.1002/ece3.6492 (PMC7391562; doi:10.1002/ece3.6492)
Supplement: Supplementary file 4 — Appendix S4 [file ECE3-10-7686-s004.docx]

**Appendix 4**

1. **Bias corrections of real and simulated data**

In the main text we explained how we assessed corrections on the real datasets (RD) testing 40 models per study area. These resulted from the interaction of eight correction methods (Spatial rarefaction, SR and Gaussian Density Kernels, GK, at four radii each) with the top five models. In addition, we ran 90 models per study area, testing the simulated datasets (spatially biased, SB, and full random, FR) in their raw and corrected versions, using the variables from the top five models. We evaluated corrections and models performances relying on the averages of evaluation metrics across the five models, for each bias situation. Results are summarized in Table 6, main text, and in Tables 1-3, this Appendix. Considering the average of the models for each combination of dataset *x* correction type, we observed in both study areas a decrease in AUC values as the spatial bias of occurrences was reduced (Table 6, main text; Table 1, Appendix 4). Increased radii of rarefaction always resulted in progressively lower AUCs, regardless of the bias condition (Table 6, main text; Table 1, Appendix 4). AUC_diff_ was always augmented by SR, at different intensities across the datasets. The degree of increase is less pronounced as the bias of occurrences is progressively reduced, and it is usually higher with increasing radius of rarefaction. SR corrections in all datasets tended to increase the average omission rates in both study areas (Table 6, main text; Table 1, Appendix 4). When the occurrences tended to be uniformly distributed in space, this correction, at a small radius, reduced the omission rate (QMLNR_FR_SR1200) or achieved equal performance with respect to a raw model (QLSNP_FR_SR1200) (Table 1, Appendix 4). Performance of GKs appeared to be more robust across different spatial configurations of occurrences. In QLSNP_RD, GKs progressively improved AUC with increasing radius up to 4800 meters, whereas no improvement was observed in QLSNP_SB and QLSNP_FR datasets (Table 6, main text; Table 1, Appendix 4).

In QMLNR, across all datasets, GKs produced average AUC values equal to, or slightly lower than, the average of the corresponding raw models, with very little variation across kernel radii (Table 6, main text; Table 1, Appendix 4).

In both study areas, and across all datasets, GKs on average resulted in equal performance or slightly higher AUC_diff_ values than the corresponding average of the raw models (Table 6, main text; Table 1, Appendix 4).

MTSS omission rate was always decreased by GKs in QLSNP for RD up to 4800 meters, In QLSNP_SB, GKs at 2400 and 9600 meters reduced the average omission rate. In the FR scenario, GKs at 2400 and 4800 achieved a slight improvement with respect of the FR_RAW average (Table 6, main text; Table 1, Appendix 4). In QMLNR, GK corrections decreased omission rate in the QMLNR_RD and FR datasets at all radii, and at 1200 and 2400 meters in QMLNR_SB dataset (Table 6, main text; Table 1, Appendix 4).

| **QLSNP** | **Models** | **N** | **AUC** | **AUC_diff_** | **MTSS** | **MTSS om** | **QMLNR** | **Models** | **N** | **AUC** | **AUC_diff_** | **MTSS** | **MTSS om** |
| --- | --- | --- | --- | --- | --- | --- | --- | --- | --- | --- | --- | --- | --- |
| **SB** | QLSNP_SB_RAW | 393 | 0,756 | 0,004 | 0,422 | 0,236 | **SB** | QMLNR_SB_RAW | 220 | 0,933 | 0,003 | 0,215 | 0,096 |
|  | QLSNP_SB_SR1200 | 385 | 0,754 | 0,006 | 0,427 | 0,257 |  | QMLNR_SB_SR1200 | 182 | 0,929 | **0,003** | 0,237 | 0,112 |
|  | QLSNP_SB_SR2400 | 370 | 0,751 | 0,006 | 0,426 | 0,250 |  | QMLNR_SB_SR2400 | 134 | 0,914 | 0,005 | 0,264 | 0,121 |
|  | QLSNP_SB_SR4800 | 309 | 0,744 | 0,008 | 0,429 | 0,264 |  | QMLNR_SB_SR4800 | 80 | 0,882 | 0,013 | 0,304 | 0,160 |
|  | QLSNP_SB_SR9600 | 208 | 0,722 | 0,014 | 0,422 | 0,268 |  | QMLNR_SB_SR9600 | 41 | 0,863 | 0,021 | 0,373 | 0,199 |
|  | QLSNP_SB_GK1200 | 393 | **0,756** | **0,004** | 0,428 | 0,237 |  | QMLNR_SB_GK1200 | 220 | **0,933** | **0,003** | 0,239 | **0,095** |
|  | QLSNP_SB_GK2400 | 393 | 0,755 | **0,004** | 0,425 | **0,229** |  | QMLNR_SB_GK2400 | 220 | **0,933** | **0,003** | 0,276 | **0,096** |
|  | QLSNP_SB_GK4800 | 393 | 0,755 | **0,004** | 0,431 | 0,237 |  | QMLNR_SB_GK4800 | 220 | **0,933** | **0,003** | 0,355 | 0,099 |
|  | QLSNP_SB_GK9600 | 393 | 0,754 | **0,004** | 0,441 | **0,227** |  | QMLNR_SB_GK9600 | 220 | 0,929 | 0,004 | 0,439 | 0,104 |
| **FR** | QLSNP_FR_RAW | 393 | 0,735 | 0,004 | 0,411 | 0,152 | **FR** | QMLNR_FR_RAW | 220 | 0,910 | 0,003 | 0,227 | 0,142 |
|  | QLSNP_FR_SR1200 | 390 | **0,735** | **0,004** | 0,412 | **0,152** |  | QMLNR_FR_SR1200 | 207 | 0,905 | 0,004 | 0,234 | **0,141** |
|  | QLSNP_FR_SR2400 | 385 | 0,734 | 0,006 | 0,415 | 0,158 |  | QMLNR_FR_SR2400 | 173 | 0,896 | 0,005 | 0,259 | 0,150 |
|  | QLSNP_FR_SR4800 | 353 | 0,731 | 0,006 | 0,407 | 0,158 |  | QMLNR_FR_SR4800 | 131 | 0,871 | 0,009 | 0,310 | 0,173 |
|  | QLSNP_FR_SR9600 | 276 | 0,716 | 0,012 | 0,406 | 0,160 |  | QMLNR_FR_SR9600 | 82 | 0,856 | 0,008 | 0,344 | 0,184 |
|  | QLSNP_FR_GK1200 | 393 | **0,735** | **0,004** | 0,413 | 0,153 |  | QMLNR_FR_GK1200 | 220 | **0,910** | **0,003** | 0,247 | **0,141** |
|  | QLSNP_FR_GK2400 | 393 | **0,735** | **0,004** | 0,413 | **0,151** |  | QMLNR_FR_GK2400 | 220 | **0,910** | **0,003** | 0,264 | **0,141** |
|  | QLSNP_FR_GK4800 | 393 | **0,735** | **0,004** | 0,417 | **0,150** |  | QMLNR_FR_GK4800 | 220 | **0,911** | **0,003** | 0,308 | **0,136** |
|  | QLSNP_FR_GK9600 | 393 | 0,734 | 0,005 | 0,426 | 0,157 |  | QMLNR_FR_GK9600 | 220 | **0,910** | **0,003** | 0,375 | **0,125** |

**Appendix 4, Table 1. Performances of correction methods, reported as average of the five top models, for the SB (simulated biased) and FR (full random) datasets, in Qilianshan National Park (QLSNP) and Qomolangma National Nature Reserve (QMLNR). MTSS=Maximum training sensitivity plus specificity logistic threshold; MTSS om= omission rate for MTSS threshold. Values in bold represent improvement or equal performance with respect to the average of the raw models.**

1. **Perfomance of bias correction methods**

We used simulation modeling to evaluate two sampling bias corrections methods, represented by spatial rarefaction (SR) and Gaussian density kernel (GK), across three datasets showing different bias intensity, coupling these optimizations with a reduced background based on ecological observations. We found that, regardless of the bias condition, GKs performed better than SRs, which is consistent with past research (Vergara et al., 2015) (Table 6, main text; Table 1, Appendix 4).

Our results also confirm that the evaluation metric AUC, which quantifies the ability of the algorithm to correctly distinguish presences from background points, does not always represent a reliable indicator of model performance (Lobo et al., 2008; Veloz, 2009; Hjimans, 2012; Jiménez-Valverde, 2012; Fourcade et al., 2014). Our results showed that AUC values were always higher for the biased RD datasets (Table 6), as a consequence of autocorrelation of training and test sets (Veloz, 2009; Chapman et al., 2010; Merckx et al., 2011; Hijmans, 2012) (Table 6, main text; Table 1, Appendix 4). Previous studies have highlighted how the performance of AUC is highly correlated with the specialist or generalist nature of the observed species (reflected by the spatial configuration of records), with lower values for this metric associated with increased area of distribution or lower habitat specialization (Phillips et al., 2006; Lobo et al., 2008; Merckx et al., 2011; Jiménez-Valverde, 2012). Therefore, a more reliable method to assess model performance is based on estimates of omission errors (Kramer-Scahdt et al., 2013; Liu et al., 2013; Shchlegovitova and Anderson, 2013; Boria et al., 2014; Radosavljevic and Anderson, 2014; Vergara et al., 2015).

Previous SDMs found that rarefying occurrences could improve model performance (Kramer-Schadt et al., 2013; Shchlegovitova and Anderson, 2013; Boria et al., 2014, Fourcade et al. 2014). In a biased context (RD scenarios), this correction on average caused a reduction of discrimination ability (Veloz, 2009; Merckx et al., 2011; Kramer-Schadt et al., 2013; Radosavljevic and Andreson, 2014; Vergara et al., 2015) and an increase in the omission rate (Vergara et al., 2015) (Table 6, main text; Table 1, Appendix 4). The same correction appeared to be ineffective even when samples became more representative of the potential environments for the species, but were still clustered geographically in one or two main areas (SB scenarios). In contrast, as bias was progressively reduced (FR scenarios), SR at radii of 1200 meters on average slightly improved (QMLNR_FR) or reached equal performance with the average of raw models (QLSNP_FR) (Table 1, Appendix 4).

Across all datasets, AUC_diff_ increased substantially only at the largest radii of SR (Vergara et al. 2015), with biased raw models performing similarly to the best GK corrections (Table 6, main text; Table 1, Appendix 4). This is inconsistent with theoretical expectations (Veloz et al., 2009; Boria et al., 2014, Radosavljevic and Anderson, 2014; Vergara et al. 2015), which would suggest that raw biased models would show strong overfitting signals. This is due to the fact that we corrected a priori for sampling bias by applying a reduced background mask, which itself can reduce AUC_diff_ considerably (Vergara et al., 2015).

In our situations, models were most improved on average by applying GKs surfaces (Kramer-Schadt et al., 2013; Fourcade et al., 2014; Vergara et al., 2015), which were more robust than spatial rarefaction, producing average AUCs higher or with similar performance to the average of the biased raw models (Kramer-Schadt et al., 2013; Vergara et al., 2015) and more effective at reducing the average omission rate (Vergara et al. 2015) (Table 6, main text; Table 1, Appendix 4). Overall, our study suggests that SRs can be efficient when the occurrences are widespread over the extent (Kramer-Scadht et al., 2013; Shchlegovitova and Anderson, 2013; Boria et al., 2014; Fourcade et al. 2014) (Table 1, Appendix 4). GKs are preferable when records are heavily clustered in space, but perform well under any occurrence configuration, representing thus one of the most reliable correction types (Vergara et al. 2015, this study).

We note that we assessed these corrections based the average performance of competing models, a strategy useful for any situation in which the objective of a study is to depict species distribution probabilistically by ensembling a range of alternative surfaces, and finding a correction method common to all of them. We found variation among models, which were dependent on their variables and the sample size after rarefaction, with SR performing better than GK in few single models across all datasets (Tables 2 and 3, Appendix 4).

Each modeling situation should be carefully analyzed within the context imposed by extent and occurrence records (Veloz, 2009; Kramer-Schadt et al., 2013; Syfert et al., 2013; Boria et al., 2014; Varela et al., 2014, Fourcade et al., 2014; Vergara et al., 2015), and corrections (or their combinations) should be assessed at several radii. However, our results suggest high robustness of GK methods overall, and SRs when observation density is relatively low over large extents. We cannot exclude a slightly different performance if we varied background radii or with removal of the background constraint. However, given the spatial configuration of samples in the three datasets, we believe that the patterns of optimization applied in this study would produce similar trends as those observed (Veloz, 2009; Kramer-Schadt et al., 2013; Fourcade et al., 2014), although removal of background constraints will certainly increase AUC (Chefaoui and Lobo, 2008; Acevedo et al., 2012), and AUC_diff_ (Veloz et al., 2009; Boria et al., 2014, Radosavljevic and Anderson, 2014; Vergara et al. 2015).

1. **Influence of simulations on correction choice**

We evaluated the perfomance of the two corrections for RD datasets on the basis of the niche similarity index D (Schoener, 1968) and the metric ΔD_geo_ (Fourcade et al., 2014) with respect to a simulated truth, created from occurrences probabilistically related to the raw distributions of the best five models in each study area. In addition, we based our selection on the MTSS omission rate (Liu et al., 2013; Vergara et al., 2015). The use of simulations adopting reference (Veloz, 2009; Warren and Seifert, 2011; Hijmans, 2012; Fourcade et al., 2014) or null (Raes and ter Steege, 2007; Beale et al., 2008; Chapman , 2010; Merckx et al., 2011) models is widespread in the SDM literature. Usually null models rely on randomizing presence points on environmental space (Raes and ter Steege, 2007, Beale et al. 2008; Merckx et al., 2011), randomizing environmental covariates to prevent their autocorrelation at sampling localities (Chapman, 2010), or simulating occurrences based on simple rule-based expectations (Cushman et al. 2017).

These strategies themselves are theoretically appealing, but suffer from limitations in the context of model selection. For example, they are only able to identify the relative performance of real datasets versus their randomized versions, identifying if records or associated variables are more clustered than random, suggesting that inflated measures of AUC are related both to spatial (Raes and ter Steege, 2007, Beale et al. 2008; Merckx et al., 2011) or environmental (Chapman, 2010) autocorrelation. This provides little insight on the models’ accuracy (and thus the validity of prediction), since the behaviour of real and simulated AUCs is to be expected given spatial autocorrelation of occurrences or predictors (Phillips et al., 2006; Lobo et al., 2008; Veloz, 2009; Chapman, 2010; Merckx et al., 2011; Hijmans, 2012; Jiménez-Valverde, 2012). Furthermore, random records might or might not be ecologically related to the original probability distribution inferred through real data, which causes the simulated distribution to indeed represent a null situation, but with little informative power for bias correction applied to a conservation context.

Our framework has several particular strengths as a means to evaluate model performance and bias correction. First, by using a “known” habitat relationship for the analysis we were able to assess the true performance of models, in terms of variables included, scales of those relationships, and pattern of prediction relative to the actual underlying relationship. This is critical and can only be supplied by simulation (e.g., Cushman 2014). Empirical modeling is always an inductive process of inferring a relationship from patterns in the data. Simulation, critically, provides a solid basis for assessing model performance in reference to a known underlying relationship. Second, our approach allowed us to assess the effects of different degrees and patterns of spatial bias by selecting simulated occurrence points (all driven by the known underlying relationship) in different spatial patterns. This enabled us to control the spatial bias as well as the underlying habitat relationship, which in combination gives us full ability to reliably assess both model performance and bias correction methods.

Evaluating the degree of overlap with a reference “truth” enables the assessment of the performance of correction types to verify how much the bias caused by the spatial configuration of records is reduced after applying a given optimization, by selecting the correction maximizing this overlap (Veloz, 2009; Fourcade et al., 2014). However, we emphasize that this reference model must be probabilistically related to the real data, i.e. should depict the distribution as if the samples were indeed collected from each point in the environmental space in which the prediction, based on the raw data, yielded positive relative suitability values. We also emphasize that maximising D or ΔD_geo_ alone might be insufficient, as they express only the relative performance that an optimized model might have with respect to a simulated truth (Veloz, 2009), and may lead to overpredictions and/or reduced accuracy when considered alone (as shown by the highest overlap reached in RD scenarios by several SR radii across the two areas, Table 6 in the main text). Many previous examples suggested minimization of omission rates at a given threshold for model selection (Kramer-Schadt et al., 2013, Radosavljevic and Anderson, 2014, Schlegovitova and Anderson, 2013; Boria et al., 2014; Vergara et al. 2015), evaluating how these metrics improve across corrections or regularization tuning.

This might suggest that one should select the correction yielding in absolute terms the lowest omission rate. However this would be incorrect without providing a reference context with respect to a null model, created upon the original distribution. An example of this is given by the performance of MTSS omission rate in SB and FR scenarios (Appendix 4, Table 1), in which many several large radii of GKs improve such metric in absolute terms. In a situation in which occurrences are spread-out in space, a corrected model might overlap with a reference model less than the raw model (as a correction would exceedingly overpredict suitable area), causing the ΔD_geo_ to be negative (Fourcade et al., 2014). This would decrease the performance with respect to the raw model (causing no improvement at all), even if the absolute magnitude of the omission rate for that given correction would allow its selection.

In order to assess the performance of a correction method in conservation contexts, it is useful to evaluate how well the correction minimizes the difference of predicted habitat across geographical space with a reference model (probabilistically related to the real data), and how much the omission rate of the optimized models is reduced respect to the uncorrected biased model, which is a proxy for model accuracy (Liu et al., 2013; Boria et al. 2014, Radosavljevic and Anderson, 2014; Vergara et al., 2015). Balancing these two metrics thus ensure a trade-off between overprediction and accuracy, providing a conservative estimate of suitable area.

| **Real dataset (RD)** | | |  |  |  |  | **Simulated biased (SB)** |  |  |  |  |  |  | **Full Random (FR)** |  |  |  |  |  |  |
| --- | --- | --- | --- | --- | --- | --- | --- | --- | --- | --- | --- | --- | --- | --- | --- | --- | --- | --- | --- | --- |
| **Model** | **N** | **AUC** | **AUC_diff_** | **MTSS** | **Area** | **MTSS _om_** | **Model** | **N** | **AUC** | **AUC_diff_** | **MTSS** | **Area** | **MTSS _om_** | **Model** | **N** | **AUC** | **AUC_diff_** | **MTSS** | **Area** | **MTSS _om_** |
| **QLSNP_1_RD_RAW** | 393 | 0,853 | 0,008 | 0,370 | 0,200 | 0,253 | **QLSNP_1_SB_RAW** | 393 | 0,757 | 0,003 | 0,407 | 0,438 | 0,176 | **QLSNP_1_FR_RAW** | 393 | 0,735 | 0,005 | 0,417 | 0,477 | 0,171 |
| *QLSNP_1_RD_SR_1200* | 219 | 0,836 | 0,010 | 0,409 | 0,211 | 0,247 | *QLSNP_1_SB_SR_1200* | 385 | 0,755 | 0,006 | 0,425 | 0,399 | 0,226 | *QLSNP_1_FR_SR_1200* | 390 | 0,734 | 0,005 | 0,418 | 0,478 | 0,164 |
| *QLSNP_1_RD_SR_2400* | 158 | 0,820 | 0,009 | 0,434 | 0,194 | 0,305 | *QLSNP_1_SB_SR_2400* | 370 | 0,753 | 0,006 | 0,414 | 0,423 | 0,219 | *QLSNP_1_FR_SR_2400* | 385 | 0,732 | 0,007 | 0,426 | 0,465 | 0,176 |
| *QLSNP_1_RD_SR_4800* | 103 | 0,772 | 0,017 | 0,448 | 0,180 | 0,435 | *QLSNP_1_SB_SR_4800* | 309 | 0,746 | 0,007 | 0,419 | 0,417 | 0,233 | *QLSNP_1_FR_SR_4800* | 353 | 0,728 | 0,007 | 0,403 | 0,504 | 0,156 |
| *QLSNP_1_RD_SR_9600* | 65 | 0,757 | 0,032 | 0,442 | 0,207 | 0,388 | *QLSNP_1_SB_SR_9600* | 208 | 0,722 | 0,016 | 0,410 | 0,456 | 0,260 | *QLSNP_1_FR_SR_9600* | 276 | 0,716 | 0,014 | 0,404 | 0,511 | 0,173 |
| *QLSNP_1_RD_GK_1200* | 393 | 0,857 | 0,008 | 0,398 | 0,194 | 0,253 | *QLSNP_1_SB_GK_1200* | 393 | 0,757 | 0,003 | 0,418 | 0,425 | 0,183 | *QLSNP_1_FR_GK_1200* | 393 | 0,735 | 0,004 | 0,421 | 0,474 | 0,176 |
| *QLSNP_1_RD_GK_2400* | 393 | 0,859 | 0,009 | 0,424 | 0,177 | 0,248 | *QLSNP_1_SB_GK_2400* | 393 | 0,757 | 0,003 | 0,413 | 0,437 | 0,178 | *QLSNP_1_FR_GK_2400* | 393 | 0,735 | 0,004 | 0,418 | 0,479 | 0,171 |
| *QLSNP_1_RD_GK_4800* | 393 | 0,861 | 0,009 | 0,450 | 0,163 | 0,266 | *QLSNP_1_SB_GK_4800* | 393 | 0,757 | 0,003 | 0,420 | 0,431 | 0,201 | *QLSNP_1_FR_GK_4800* | 393 | 0,735 | 0,005 | 0,422 | 0,478 | 0,160 |
| *QLSNP_1_RD_GK_9600* | 393 | 0,855 | 0,009 | 0,477 | 0,138 | 0,302 | *QLSNP_1_SB_GK_9600* | 393 | 0,756 | 0,003 | 0,434 | 0,425 | 0,206 | *QLSNP_1_FR_GK_9600* | 393 | 0,734 | 0,005 | 0,428 | 0,480 | 0,168 |
|  |  |  |  |  |  |  |  |  |  |  |  |  |  |  |  |  |  |  |  |  |
| **QLSNP_2_RD_RAW** | 393 | 0,856 | 0,009 | 0,424 | 0,143 | 0,296 | **QLSNP_2_SB_RAW** | 393 | 0,757 | 0,004 | 0,446 | 0,355 | 0,270 | **QLSNP_2_FR_RAW** | 393 | 0,733 | 0,002 | 0,402 | 0,512 | 0,135 |
| *QLSNP_2_RD_SR_1200* | 219 | 0,835 | 0,010 | 0,456 | 0,138 | 0,343 | *QLSNP_2_SB_SR_1200* | 385 | 0,755 | 0,006 | 0,451 | 0,345 | 0,283 | *QLSNP_2_FR_SR_1200* | 390 | 0,733 | 0,002 | 0,403 | 0,513 | 0,128 |
| *QLSNP_2_RD_SR_2400* | 158 | 0,816 | 0,009 | 0,435 | 0,183 | 0,305 | *QLSNP_2_SB_SR_2400* | 370 | 0,753 | 0,006 | 0,462 | 0,324 | 0,300 | *QLSNP_2_FR_SR_2400* | 385 | 0,733 | 0,003 | 0,404 | 0,511 | 0,127 |
| *QLSNP_2_RD_SR_4800* | 103 | 0,766 | 0,018 | 0,469 | 0,126 | 0,433 | *QLSNP_2_SB_SR_4800* | 309 | 0,746 | 0,007 | 0,461 | 0,334 | 0,310 | *QLSNP_2_FR_SR_4800* | 353 | 0,729 | 0,003 | 0,406 | 0,510 | 0,139 |
| *QLSNP_2_RD_SR_9600* | 65 | 0,753 | 0,031 | 0,466 | 0,145 | 0,431 | *QLSNP_2_SB_SR_9600* | 208 | 0,724 | 0,013 | 0,457 | 0,357 | 0,322 | *QLSNP_2_FR_SR_9600* | 276 | 0,714 | 0,011 | 0,399 | 0,534 | 0,122 |
| *QLSNP_2_RD_GK_1200* | 393 | 0,860 | 0,009 | 0,440 | 0,145 | 0,290 | *QLSNP_2_SB_GK_1200* | 393 | 0,757 | 0,004 | 0,450 | 0,354 | 0,270 | *QLSNP_2_FR_GK_1200* | 393 | 0,733 | 0,002 | 0,405 | 0,512 | 0,135 |
| *QLSNP_2_RD_GK_2400* | 393 | 0,860 | 0,009 | 0,447 | 0,145 | 0,287 | *QLSNP_2_SB_GK_2400* | 393 | 0,757 | 0,004 | 0,451 | 0,355 | 0,267 | *QLSNP_2_FR_GK_2400* | 393 | 0,733 | 0,002 | 0,404 | 0,515 | 0,132 |
| *QLSNP_2_RD_GK_4800* | 393 | 0,861 | 0,009 | 0,468 | 0,129 | 0,281 | *QLSNP_2_SB_GK_4800* | 393 | 0,757 | 0,004 | 0,452 | 0,359 | 0,272 | *QLSNP_2_FR_GK_4800* | 393 | 0,733 | 0,003 | 0,408 | 0,511 | 0,130 |
| *QLSNP_2_RD_GK_9600* | 393 | 0,852 | 0,009 | 0,481 | 0,127 | 0,305 | *QLSNP_2_SB_GK_9600* | 393 | 0,756 | 0,004 | 0,455 | 0,377 | 0,255 | *QLSNP_2_FR_GK_9600* | 393 | 0,732 | 0,003 | 0,416 | 0,511 | 0,132 |
|  |  |  |  |  |  |  |  |  |  |  |  |  |  |  |  |  |  |  |  |  |
| **QLSNP_4_RD_RAW** | 393 | 0,856 | 0,009 | 0,427 | 0,141 | 0,293 | **QLSNP_4_SB_RAW** | 393 | 0,749 | 0,005 | 0,438 | 0,374 | 0,270 | **QLSNP_4_FR_RAW** | 393 | 0,732 | 0,003 | 0,415 | 0,491 | 0,171 |
| *QLSNP_4_RD_SR_1200* | 219 | 0,837 | 0,008 | 0,395 | 0,230 | 0,228 | *QLSNP_4_SB_SR_1200* | 385 | 0,749 | 0,006 | 0,441 | 0,369 | 0,283 | *QLSNP_4_FR_SR_1200* | 390 | 0,732 | 0,002 | 0,425 | 0,477 | 0,185 |
| *QLSNP_4_RD_SR_2400* | 158 | 0,816 | 0,009 | 0,437 | 0,180 | 0,330 | *QLSNP_4_SB_SR_2400* | 370 | 0,745 | 0,007 | 0,450 | 0,352 | 0,295 | *QLSNP_4_FR_SR_2400* | 385 | 0,730 | 0,004 | 0,422 | 0,479 | 0,174 |
| *QLSNP_4_RD_SR_4800* | 103 | 0,762 | 0,022 | 0,455 | 0,146 | 0,423 | *QLSNP_4_SB_SR_4800* | 309 | 0,739 | 0,008 | 0,449 | 0,363 | 0,320 | *QLSNP_4_FR_SR_4800* | 353 | 0,729 | 0,003 | 0,408 | 0,506 | 0,165 |
| *QLSNP_4_RD_SR_9600* | 65 | 0,752 | 0,034 | 0,463 | 0,148 | 0,431 | *QLSNP_4_SB_SR_9600* | 208 | 0,719 | 0,012 | 0,433 | 0,410 | 0,298 | *QLSNP_4_FR_SR_9600* | 276 | 0,713 | 0,011 | 0,403 | 0,526 | 0,148 |
| *QLSNP_4_RD_GK_1200* | 393 | 0,858 | 0,009 | 0,436 | 0,151 | 0,287 | *QLSNP_4_SB_GK_1200* | 393 | 0,749 | 0,005 | 0,440 | 0,376 | 0,270 | *QLSNP_4_FR_GK_1200* | 393 | 0,732 | 0,003 | 0,417 | 0,492 | 0,168 |
| *QLSNP_4_RD_GK_2400* | 393 | 0,859 | 0,009 | 0,447 | 0,148 | 0,281 | *QLSNP_4_SB_GK_2400* | 393 | 0,749 | 0,005 | 0,444 | 0,372 | 0,273 | *QLSNP_4_FR_GK_2400* | 393 | 0,732 | 0,003 | 0,418 | 0,491 | 0,168 |
| *QLSNP_4_RD_GK_4800* | 393 | 0,861 | 0,008 | 0,464 | 0,135 | 0,278 | *QLSNP_4_SB_GK_4800* | 393 | 0,749 | 0,005 | 0,447 | 0,374 | 0,273 | *QLSNP_4_FR_GK_4800* | 393 | 0,732 | 0,003 | 0,422 | 0,490 | 0,168 |
| *QLSNP_4_RD_GK_9600* | 393 | 0,850 | 0,010 | 0,470 | 0,144 | 0,281 | *QLSNP_4_SB_GK_9600* | 393 | 0,748 | 0,005 | 0,443 | 0,413 | 0,216 | *QLSNP_4_FR_GK_9600* | 393 | 0,731 | 0,003 | 0,431 | 0,483 | 0,168 |
|  |  |  |  |  |  |  |  |  |  |  |  |  |  |  |  |  |  |  |  |  |
| **QLSNP_6_RD_RAW** | 393 | 0,864 | 0,008 | 0,377 | 0,193 | 0,230 | **QLSNP_6_SB_RAW** | 393 | 0,757 | 0,005 | 0,401 | 0,421 | 0,227 | **QLSNP_6_FR_RAW** | 393 | 0,737 | 0,005 | 0,404 | 0,495 | 0,127 |
| *QLSNP_6_RD_SR_1200* | 219 | 0,840 | 0,008 | 0,411 | 0,199 | 0,278 | *QLSNP_6_SB_SR_1200* | 385 | 0,757 | 0,006 | 0,404 | 0,409 | 0,244 | *QLSNP_6_FR_SR_1200* | 390 | 0,739 | 0,003 | 0,404 | 0,495 | 0,123 |
| *QLSNP_6_RD_SR_2400* | 158 | 0,820 | 0,009 | 0,422 | 0,203 | 0,285 | *QLSNP_6_SB_SR_2400* | 370 | 0,754 | 0,006 | 0,407 | 0,407 | 0,246 | *QLSNP_6_FR_SR_2400* | 385 | 0,737 | 0,005 | 0,408 | 0,488 | 0,146 |
| *QLSNP_6_RD_SR_4800* | 103 | 0,764 | 0,029 | 0,430 | 0,197 | 0,423 | *QLSNP_6_SB_SR_4800* | 309 | 0,745 | 0,009 | 0,415 | 0,406 | 0,252 | *QLSNP_6_FR_SR_4800* | 353 | 0,734 | 0,006 | 0,406 | 0,495 | 0,165 |
| *QLSNP_6_RD_SR_9600* | 65 | 0,756 | 0,038 | 0,454 | 0,193 | 0,400 | *QLSNP_6_SB_SR_9600* | 208 | 0,724 | 0,013 | 0,412 | 0,438 | 0,241 | *QLSNP_6_FR_SR_9600* | 276 | 0,718 | 0,011 | 0,420 | 0,491 | 0,170 |
| *QLSNP_6_RD_GK_1200* | 393 | 0,866 | 0,008 | 0,404 | 0,187 | 0,239 | *QLSNP_6_SB_GK_1200* | 393 | 0,757 | 0,005 | 0,412 | 0,407 | 0,234 | *QLSNP_6_FR_GK_1200* | 393 | 0,737 | 0,005 | 0,407 | 0,494 | 0,127 |
| *QLSNP_6_RD_GK_2400* | 393 | 0,866 | 0,009 | 0,416 | 0,185 | 0,236 | *QLSNP_6_SB_GK_2400* | 393 | 0,757 | 0,005 | 0,403 | 0,429 | 0,211 | *QLSNP_6_FR_GK_2400* | 393 | 0,737 | 0,005 | 0,408 | 0,493 | 0,125 |
| *QLSNP_6_RD_GK_4800* | 393 | 0,868 | 0,009 | 0,438 | 0,174 | 0,236 | *QLSNP_6_SB_GK_4800* | 393 | 0,757 | 0,005 | 0,408 | 0,431 | 0,214 | *QLSNP_6_FR_GK_4800* | 393 | 0,737 | 0,005 | 0,409 | 0,496 | 0,127 |
| *QLSNP_6_RD_GK_9600* | 393 | 0,860 | 0,010 | 0,467 | 0,151 | 0,281 | *QLSNP_6_SB_GK_9600* | 393 | 0,756 | 0,005 | 0,432 | 0,404 | 0,232 | *QLSNP_6_FR_GK_9600* | 393 | 0,736 | 0,005 | 0,421 | 0,489 | 0,145 |
|  |  |  |  |  |  |  |  |  |  |  |  |  |  |  |  |  |  |  |  |  |
| **QLSNP_7_RD_RAW** | 393 | 0,859 | 0,008 | 0,390 | 0,170 | 0,284 | **QLSNP_7_SB_RAW** | 393 | 0,757 | 0,004 | 0,418 | 0,396 | 0,237 | **QLSNP_7_FR_RAW** | 393 | 0,739 | 0,007 | 0,415 | 0,464 | 0,158 |
| *QLSNP_7_RD_SR_1200* | 219 | 0,838 | 0,009 | 0,406 | 0,210 | 0,265 | *QLSNP_7_SB_SR_1200* | 385 | 0,757 | 0,006 | 0,416 | 0,393 | 0,250 | *QLSNP_7_FR_SR_1200* | 390 | 0,740 | 0,006 | 0,413 | 0,467 | 0,162 |
| *QLSNP_7_RD_SR_2400* | 158 | 0,823 | 0,008 | 0,394 | 0,253 | 0,228 | *QLSNP_7_SB_SR_2400* | 370 | 0,753 | 0,007 | 0,397 | 0,436 | 0,189 | *QLSNP_7_FR_SR_2400* | 385 | 0,736 | 0,010 | 0,415 | 0,465 | 0,166 |
| *QLSNP_7_RD_SR_4800* | 103 | 0,768 | 0,029 | 0,415 | 0,235 | 0,395 | *QLSNP_7_SB_SR_4800* | 309 | 0,745 | 0,009 | 0,401 | 0,439 | 0,207 | *QLSNP_7_FR_SR_4800* | 353 | 0,733 | 0,010 | 0,412 | 0,472 | 0,167 |
| *QLSNP_7_RD_SR_9600* | 65 | 0,763 | 0,036 | 0,440 | 0,216 | 0,386 | *QLSNP_7_SB_SR_9600* | 208 | 0,721 | 0,017 | 0,398 | 0,478 | 0,217 | *QLSNP_7_FR_SR_9600* | 276 | 0,722 | 0,013 | 0,402 | 0,504 | 0,188 |
| *QLSNP_7_RD_GK_1200* | 393 | 0,862 | 0,008 | 0,409 | 0,176 | 0,269 | *QLSNP_7_SB_GK_1200* | 393 | 0,757 | 0,004 | 0,418 | 0,402 | 0,229 | *QLSNP_7_FR_GK_1200* | 393 | 0,739 | 0,007 | 0,418 | 0,463 | 0,158 |
| *QLSNP_7_RD_GK_2400* | 393 | 0,864 | 0,009 | 0,412 | 0,187 | 0,260 | *QLSNP_7_SB_GK_2400* | 393 | 0,757 | 0,004 | 0,415 | 0,414 | 0,214 | *QLSNP_7_FR_GK_2400* | 393 | 0,739 | 0,007 | 0,417 | 0,465 | 0,158 |
| *QLSNP_7_RD_GK_4800* | 393 | 0,867 | 0,009 | 0,419 | 0,206 | 0,217 | *QLSNP_7_SB_GK_4800* | 393 | 0,757 | 0,004 | 0,427 | 0,400 | 0,227 | *QLSNP_7_FR_GK_4800* | 393 | 0,739 | 0,007 | 0,422 | 0,461 | 0,163 |
| *QLSNP_7_RD_GK_9600* | 393 | 0,860 | 0,009 | 0,462 | 0,164 | 0,281 | *QLSNP_7_SB_GK_9600* | 393 | 0,757 | 0,004 | 0,442 | 0,390 | 0,224 | *QLSNP_7_FR_GK_9600* | 393 | 0,738 | 0,008 | 0,435 | 0,453 | 0,173 |
|  |  |  |  |  |  |  |  |  |  |  |  |  |  |  |  |  |  |  |  |  |
| **Average** | **N** | **AUC** | **AUC_diff_** | **MTSS** | **Area** | **MTSS _om_** | **Average** | **N** | **AUC** | **AUC_diff_** | **MTSS** | **Area** | **MTSS _om_** | **Average** | **N** | **AUC** | **AUC_diff_** | **MTSS** | **Area** | **MTSS _om_** |
| **QLSNP_RD_RAW** | 393 | 0,858 | 0,008 | 0,398 | 0,169 | 0,271 | **QLSNP_SB_RAW** | 393 | 0,756 | 0,004 | 0,422 | 0,397 | 0,236 | **QLSNP_FR_RAW** | 393 | 0,735 | 0,004 | 0,411 | 0,488 | 0,152 |
| *QLSNP_RD_SR_1200* | 219 | 0,837 | 0,009 | 0,415 | 0,198 | 0,272 | *QLSNP_SB_SR_1200* | 385 | 0,754 | 0,006 | 0,427 | 0,383 | 0,257 | *QLSNP_FR_SR_1200* | 390 | 0,735 | 0,004 | 0,412 | 0,486 | 0,152 |
| *QLSNP_RD_SR_2400* | 158 | 0,819 | 0,009 | 0,424 | 0,203 | 0,290 | *QLSNP_SB_SR_2400* | 370 | 0,751 | 0,006 | 0,426 | 0,388 | 0,250 | *QLSNP_FR_SR_2400* | 385 | 0,734 | 0,006 | 0,415 | 0,482 | 0,158 |
| *QLSNP_RD_SR_4800* | 103 | 0,766 | 0,023 | 0,443 | 0,177 | 0,421 | *QLSNP_SB_SR_4800* | 309 | 0,744 | 0,008 | 0,429 | 0,391 | 0,264 | *QLSNP_FR_SR_4800* | 353 | 0,731 | 0,006 | 0,407 | 0,497 | 0,158 |
| *QLSNP_RD_SR_9600* | 65 | 0,756 | 0,034 | 0,453 | 0,182 | 0,407 | *QLSNP_SB_SR_9600* | 208 | 0,722 | 0,014 | 0,422 | 0,428 | 0,268 | *QLSNP_FR_SR_9600* | 276 | 0,716 | 0,012 | 0,406 | 0,513 | 0,160 |
| *QLSNP_RD_GK_1200* | 393 | 0,860 | 0,009 | 0,417 | 0,171 | 0,268 | *QLSNP_SB_GK_1200* | 393 | 0,756 | 0,004 | 0,428 | 0,393 | 0,237 | *QLSNP_FR_GK_1200* | 393 | 0,735 | 0,004 | 0,413 | 0,487 | 0,153 |
| *QLSNP_RD_GK_2400* | 393 | 0,862 | 0,009 | 0,429 | 0,168 | 0,262 | *QLSNP_SB_GK_2400* | 393 | 0,755 | 0,004 | 0,425 | 0,401 | 0,229 | *QLSNP_FR_GK_2400* | 393 | 0,735 | 0,004 | 0,413 | 0,489 | 0,151 |
| *QLSNP_RD_GK_4800* | 393 | 0,863 | 0,009 | 0,448 | 0,162 | 0,255 | *QLSNP_SB_GK_4800* | 393 | 0,755 | 0,004 | 0,431 | 0,399 | 0,237 | *QLSNP_FR_GK_4800* | 393 | 0,735 | 0,004 | 0,417 | 0,487 | 0,150 |
| *QLSNP_RD_GK_9600* | 393 | 0,855 | 0,009 | 0,471 | 0,145 | 0,290 | *QLSNP_SB_GK_9600* | 393 | 0,754 | 0,004 | 0,441 | 0,402 | 0,227 | *QLSNP_FR_GK_9600* | 393 | 0,734 | 0,005 | 0,426 | 0,483 | 0,157 |

**Appendix 4, Table 2. Performance of spatial rarefaction (SR) and Gaussian density kernel (GK) at four different radii, reported for each of the five top models and as average values, across three datasets representing different bias intensities (RD=real dataset; SB= simulated biased; FR= Full random), in Qilianshan National Park. MTSS=Maximum training sensitivity plus specificity logistic threshold; MTSS om= omission rate for MTSS threshold; Area= MTSS Area.**

| **Real dataset (RD)** | | |  |  |  |  | **Simulated biased (SB)** | | | |  |  |  | **Full Random (FR)** | | |  |  |  |  |
| --- | --- | --- | --- | --- | --- | --- | --- | --- | --- | --- | --- | --- | --- | --- | --- | --- | --- | --- | --- | --- |
| **Model** | **N** | **AUC** | **AUC_diff_** | **MTSS** | **Area** | **MTSS _om_** | **Model** | **N** | **AUC** | **AUC_diff_** | **MTSS** | **Area** | **MTSS _om_** | **Model** | **N** | **AUC** | **AUC_diff_** | **MTSS** | **Area** | **MTSS _om_** |
| **QMLNR_1_RD_RAW** | 220 | 0,975 | 0,001 | 0,136 | 0,074 | 0,071 | **QMLNR_1_SB_RAW** | 220 | 0,936 | 0,002 | 0,226 | 0,147 | 0,091 | **QMLNR_1_FR_RAW** | 220 | 0,907 | 0,002 | 0,217 | 0,199 | 0,136 |
| *QMLNR_1_RD_SR_1200* | 79 | 0,964 | 0,005 | 0,168 | 0,096 | 0,113 | *QMLNR_1_SB_SR_1200* | 182 | 0,932 | 0,003 | 0,234 | 0,155 | 0,116 | *QMLNR_1_FR_SR_1200* | 207 | 0,900 | 0,005 | 0,240 | 0,197 | 0,145 |
| *QMLNR_1_RD_SR_2400* | 49 | 0,948 | 0,008 | 0,257 | 0,115 | 0,145 | *QMLNR_1_SB_SR_2400* | 134 | 0,917 | 0,005 | 0,261 | 0,176 | 0,121 | *QMLNR_1_FR_SR_2400* | 173 | 0,892 | 0,005 | 0,282 | 0,194 | 0,151 |
| *QMLNR_1_RD_SR_4800* | 28 | 0,911 | 0,015 | 0,399 | 0,140 | 0,200 | *QMLNR_1_SB_SR_4800* | 80 | 0,884 | 0,013 | 0,293 | 0,228 | 0,100 | *QMLNR_1_FR_SR_4800* | 131 | 0,867 | 0,009 | 0,303 | 0,246 | 0,166 |
| *QMLNR_1_RD_SR_9600* | 19 | 0,903 | 0,015 | 0,350 | 0,196 | 0,100 | *QMLNR_1_SB_SR_9600* | 41 | 0,861 | 0,021 | 0,349 | 0,243 | 0,150 | *QMLNR_1_FR_SR_9600* | 82 | 0,854 | 0,006 | 0,373 | 0,231 | 0,185 |
| *QMLNR_1_RD_GK_1200* | 220 | 0,975 | 0,001 | 0,204 | 0,073 | 0,077 | *QMLNR_1_SB_GK_1200* | 220 | 0,936 | 0,003 | 0,250 | 0,148 | 0,086 | *QMLNR_1_FR_GK_1200* | 220 | 0,908 | 0,002 | 0,235 | 0,198 | 0,127 |
| *QMLNR_1_RD_GK_2400* | 220 | 0,974 | 0,001 | 0,276 | 0,076 | 0,076 | *QMLNR_1_SB_GK_2400* | 220 | 0,936 | 0,003 | 0,291 | 0,146 | 0,096 | *QMLNR_1_FR_GK_2400* | 220 | 0,908 | 0,002 | 0,248 | 0,200 | 0,127 |
| *QMLNR_1_RD_GK_4800* | 220 | 0,974 | 0,001 | 0,416 | 0,073 | 0,077 | *QMLNR_1_SB_GK_4800* | 220 | 0,935 | 0,003 | 0,361 | 0,155 | 0,096 | *QMLNR_1_FR_GK_4800* | 220 | 0,908 | 0,003 | 0,300 | 0,190 | 0,118 |
| *QMLNR_1_RD_GK_9600* | 220 | 0,971 | 0,002 | 0,529 | 0,081 | 0,071 | *QMLNR_1_SB_GK_9600* | 220 | 0,931 | 0,004 | 0,430 | 0,179 | 0,096 | *QMLNR_1_FR_GK_9600* | 220 | 0,908 | 0,003 | 0,387 | 0,186 | 0,132 |
|  |  |  |  |  |  |  |  |  |  |  |  |  |  |  |  |  |  |  |  |  |
| **QMLNR_2_RD_RAW** | 220 | 0,971 | 0,001 | 0,195 | 0,067 | 0,088 | **QMLNR_2_SB_RAW** | 220 | 0,932 | 0,003 | 0,212 | 0,164 | 0,096 | **QMLNR_2_FR_RAW** | 220 | 0,915 | 0,003 | 0,209 | 0,175 | 0,123 |
| *QMLNR_2_RD_SR_1200* | 79 | 0,954 | 0,005 | 0,295 | 0,068 | 0,166 | *QMLNR_2_SB_SR_1200* | 182 | 0,929 | 0,003 | 0,241 | 0,155 | 0,110 | *QMLNR_2_FR_SR_1200* | 207 | 0,911 | 0,004 | 0,210 | 0,184 | 0,125 |
| *QMLNR_2_RD_SR_2400* | 49 | 0,942 | 0,009 | 0,185 | 0,160 | 0,085 | *QMLNR_2_SB_SR_2400* | 134 | 0,915 | 0,006 | 0,258 | 0,183 | 0,112 | *QMLNR_2_FR_SR_2400* | 173 | 0,901 | 0,004 | 0,261 | 0,179 | 0,167 |
| *QMLNR_2_RD_SR_4800* | 28 | 0,902 | 0,016 | 0,297 | 0,187 | 0,200 | *QMLNR_2_SB_SR_4800* | 80 | 0,876 | 0,017 | 0,311 | 0,226 | 0,150 | *QMLNR_2_FR_SR_4800* | 131 | 0,873 | 0,011 | 0,300 | 0,219 | 0,159 |
| *QMLNR_2_RD_SR_9600* | 19 | 0,912 | 0,010 | 0,308 | 0,198 | 0,200 | *QMLNR_2_SB_SR_9600* | 41 | 0,863 | 0,020 | 0,389 | 0,215 | 0,225 | *QMLNR_2_FR_SR_9600* | 82 | 0,857 | 0,009 | 0,350 | 0,241 | 0,197 |
| *QMLNR_2_RD_GK_1200* | 220 | 0,971 | 0,002 | 0,284 | 0,063 | 0,106 | *QMLNR_2_SB_GK_1200* | 220 | 0,932 | 0,003 | 0,231 | 0,167 | 0,091 | *QMLNR_2_FR_GK_1200* | 220 | 0,915 | 0,003 | 0,229 | 0,177 | 0,123 |
| *QMLNR_2_RD_GK_2400* | 220 | 0,970 | 0,001 | 0,372 | 0,062 | 0,112 | *QMLNR_2_SB_GK_2400* | 220 | 0,932 | 0,003 | 0,261 | 0,170 | 0,091 | *QMLNR_2_FR_GK_2200* | 220 | 0,915 | 0,003 | 0,242 | 0,183 | 0,114 |
| *QMLNR_2_RD_GK_4800* | 220 | 0,970 | 0,001 | 0,438 | 0,077 | 0,094 | *QMLNR_2_SB_GK_2800* | 220 | 0,931 | 0,004 | 0,324 | 0,181 | 0,082 | *QMLNR_2_FR_GK_2800* | 220 | 0,915 | 0,004 | 0,301 | 0,178 | 0,132 |
| *QMLNR_2_RD_GK_9600* | 220 | 0,966 | 0,002 | 0,440 | 0,121 | 0,077 | *QMLNR_2_SB_GK_9600* | 220 | 0,927 | 0,005 | 0,436 | 0,172 | 0,105 | *QMLNR_2_FR_GK_9600* | 220 | 0,915 | 0,004 | 0,384 | 0,187 | 0,127 |
|  |  |  |  |  |  |  |  |  |  |  |  |  |  |  |  |  |  |  |  |  |
| **QMLNR_4_RD_RAW** | 220 | 0,970 | 0,001 | 0,215 | 0,059 | 0,077 | **QMLNR_4_SB_RAW** | 220 | 0,929 | 0,003 | 0,190 | 0,158 | 0,105 | **QMLNR_4_FR_RAW** | 220 | 0,911 | 0,003 | 0,221 | 0,160 | 0,168 |
| *QMLNR_4_RD_SR_1200* | 79 | 0,958 | 0,004 | 0,168 | 0,114 | 0,138 | *QMLNR_4_SB_SR_1200* | 182 | 0,924 | 0,005 | 0,223 | 0,147 | 0,111 | *QMLNR_4_FR_SR_1200* | 207 | 0,907 | 0,003 | 0,223 | 0,172 | 0,170 |
| *QMLNR_4_RD_SR_2400* | 49 | 0,940 | 0,009 | 0,226 | 0,126 | 0,125 | *QMLNR_4_SB_SR_2400* | 134 | 0,910 | 0,005 | 0,265 | 0,161 | 0,127 | *QMLNR_4_FR_SR_2400* | 173 | 0,895 | 0,005 | 0,213 | 0,234 | 0,140 |
| *QMLNR_4_RD_SR_4800* | 28 | 0,901 | 0,018 | 0,267 | 0,213 | 0,167 | *QMLNR_4_SB_SR_4800* | 80 | 0,878 | 0,014 | 0,329 | 0,192 | 0,225 | *QMLNR_4_FR_SR_4800* | 131 | 0,873 | 0,009 | 0,299 | 0,225 | 0,160 |
| *QMLNR_4_RD_SR_9600* | 19 | 0,898 | 0,016 | 0,340 | 0,200 | 0,150 | *QMLNR_4_SB_SR_9600* | 41 | 0,864 | 0,022 | 0,391 | 0,199 | 0,220 | *QMLNR_4_FR_SR_9600* | 82 | 0,857 | 0,010 | 0,350 | 0,238 | 0,144 |
| *QMLNR_4_RD_GK_1200* | 220 | 0,968 | 0,001 | 0,275 | 0,062 | 0,071 | *QMLNR_4_SB_GK_1200* | 220 | 0,929 | 0,003 | 0,216 | 0,154 | 0,105 | *QMLNR_4_FR_GK_1200* | 220 | 0,912 | 0,003 | 0,249 | 0,156 | 0,173 |
| *QMLNR_4_RD_GK_2400* | 220 | 0,970 | 0,002 | 0,319 | 0,060 | 0,076 | *QMLNR_4_SB_GK_2400* | 220 | 0,929 | 0,003 | 0,255 | 0,151 | 0,091 | *QMLNR_4_FR_GK_2400* | 220 | 0,912 | 0,003 | 0,270 | 0,155 | 0,173 |
| *QMLNR_4_RD_GK_4800* | 220 | 0,969 | 0,002 | 0,419 | 0,068 | 0,076 | *QMLNR_4_SB_GK_4800* | 220 | 0,929 | 0,004 | 0,340 | 0,149 | 0,100 | *QMLNR_4_FR_GK_4800* | 220 | 0,912 | 0,003 | 0,315 | 0,165 | 0,168 |
| *QMLNR_4_RD_GK_9600* | 220 | 0,968 | 0,002 | 0,489 | 0,090 | 0,082 | *QMLNR_4_SB_GK_9600* | 220 | 0,927 | 0,004 | 0,430 | 0,154 | 0,105 | *QMLNR_4_FR_GK_9600* | 220 | 0,913 | 0,004 | 0,359 | 0,208 | 0,118 |
|  |  |  |  |  |  |  |  |  |  |  |  |  |  |  |  |  |  |  |  |  |
| **QMLNR_5_RD_RAW** | 220 | 0,971 | 0,002 | 0,260 | 0,051 | 0,118 | **QMLNR_5_SB_RAW** | 220 | 0,933 | 0,002 | 0,214 | 0,157 | 0,082 | **QMLNR_5_FR_RAW** | 220 | 0,918 | 0,003 | 0,196 | 0,172 | 0,118 |
| *QMLNR_5_RD_SR_1200* | 79 | 0,958 | 0,005 | 0,113 | 0,149 | 0,102 | *QMLNR_5_SB_SR_1200* | 182 | 0,927 | 0,004 | 0,239 | 0,159 | 0,105 | *QMLNR_5_FR_SR_1200* | 207 | 0,914 | 0,003 | 0,209 | 0,175 | 0,117 |
| *QMLNR_5_RD_SR_2400* | 49 | 0,937 | 0,013 | 0,148 | 0,173 | 0,105 | *QMLNR_5_SB_SR_2400* | 134 | 0,909 | 0,007 | 0,278 | 0,175 | 0,136 | *QMLNR_5_FR_SR_2400* | 173 | 0,905 | 0,005 | 0,235 | 0,180 | 0,145 |
| *QMLNR_5_RD_SR_4800* | 28 | 0,898 | 0,021 | 0,282 | 0,190 | 0,183 | *QMLNR_5_SB_SR_4800* | 80 | 0,880 | 0,012 | 0,291 | 0,227 | 0,163 | *QMLNR_5_FR_SR_4800* | 131 | 0,878 | 0,010 | 0,288 | 0,205 | 0,181 |
| *QMLNR_5_RD_SR_9600* | 19 | 0,895 | 0,026 | 0,322 | 0,183 | 0,200 | *QMLNR_5_SB_SR_9600* | 41 | 0,857 | 0,024 | 0,382 | 0,200 | 0,225 | *QMLNR_5_FR_SR_9600* | 82 | 0,864 | 0,007 | 0,316 | 0,230 | 0,185 |
| *QMLNR_5_RD_GK_1200* | 220 | 0,971 | 0,001 | 0,315 | 0,054 | 0,094 | *QMLNR_5_SB_GK_1200* | 220 | 0,933 | 0,003 | 0,234 | 0,159 | 0,086 | *QMLNR_5_FR_GK_1200* | 220 | 0,918 | 0,003 | 0,218 | 0,173 | 0,118 |
| *QMLNR_5_RD_GK_2400* | 220 | 0,969 | 0,002 | 0,381 | 0,060 | 0,094 | *QMLNR_5_SB_GK_2400* | 220 | 0,933 | 0,003 | 0,265 | 0,163 | 0,091 | *QMLNR_5_FR_GK_2400* | 220 | 0,918 | 0,003 | 0,243 | 0,170 | 0,127 |
| *QMLNR_5_RD_GK_4800* | 220 | 0,969 | 0,002 | 0,476 | 0,063 | 0,106 | *QMLNR_5_SB_GK_4800* | 220 | 0,932 | 0,003 | 0,355 | 0,158 | 0,109 | *QMLNR_5_FR_GK_4800* | 220 | 0,918 | 0,003 | 0,299 | 0,167 | 0,132 |
| *QMLNR_5_RD_GK_9600* | 220 | 0,965 | 0,003 | 0,513 | 0,091 | 0,100 | *QMLNR_5_SB_GK_9600* | 220 | 0,927 | 0,004 | 0,435 | 0,173 | 0,114 | *QMLNR_5_FR_GK_9600* | 220 | 0,917 | 0,004 | 0,365 | 0,188 | 0,118 |
|  |  |  |  |  |  |  |  |  |  |  |  |  |  |  |  |  |  |  |  |  |
| **QMLNR_6_RD_RAW** | 220 | 0,972 | 0,001 | 0,164 | 0,069 | 0,071 | **QMLNR_6_SB_RAW** | 220 | 0,938 | 0,002 | 0,233 | 0,123 | 0,109 | **QMLNR_6_FR_RAW** | 220 | 0,900 | 0,002 | 0,289 | 0,180 | 0,164 |
| *QMLNR_6_RD_SR_1200* | 79 | 0,958 | 0,003 | 0,185 | 0,100 | 0,127 | *QMLNR_6_SB_SR_1200* | 182 | 0,933 | 0,002 | 0,249 | 0,124 | 0,121 | *QMLNR_6_FR_SR_1200* | 207 | 0,894 | 0,003 | 0,291 | 0,191 | 0,149 |
| *QMLNR_6_RD_SR_2400* | 49 | 0,943 | 0,006 | 0,222 | 0,118 | 0,165 | *QMLNR_6_SB_SR_2400* | 134 | 0,920 | 0,004 | 0,259 | 0,156 | 0,110 | *QMLNR_6_FR_SR_2400* | 173 | 0,886 | 0,003 | 0,307 | 0,198 | 0,150 |
| *QMLNR_6_RD_SR_4800* | 28 | 0,913 | 0,005 | 0,410 | 0,119 | 0,200 | *QMLNR_6_SB_SR_4800* | 80 | 0,891 | 0,012 | 0,294 | 0,198 | 0,163 | *QMLNR_6_FR_SR_4800* | 131 | 0,862 | 0,006 | 0,362 | 0,205 | 0,198 |
| *QMLNR_6_RD_SR_9600* | 19 | 0,910 | 0,007 | 0,260 | 0,233 | 0,100 | *QMLNR_6_SB_SR_9600* | 41 | 0,873 | 0,017 | 0,355 | 0,202 | 0,175 | *QMLNR_6_FR_SR_9600* | 82 | 0,847 | 0,007 | 0,329 | 0,271 | 0,210 |
| *QMLNR_6_RD_GK_1200* | 220 | 0,970 | 0,001 | 0,237 | 0,073 | 0,076 | *QMLNR_6_SB_GK_1200* | 220 | 0,937 | 0,002 | 0,262 | 0,124 | 0,105 | *QMLNR_6_FR_GK_1200* | 220 | 0,900 | 0,002 | 0,303 | 0,178 | 0,164 |
| *QMLNR_6_RD_GK_2400* | 220 | 0,970 | 0,001 | 0,258 | 0,086 | 0,065 | *QMLNR_6_SB_GK_2400* | 220 | 0,937 | 0,003 | 0,310 | 0,124 | 0,114 | *QMLNR_6_FR_GK_2400* | 220 | 0,900 | 0,002 | 0,314 | 0,178 | 0,164 |
| *QMLNR_6_RD_GK_4800* | 220 | 0,972 | 0,001 | 0,411 | 0,077 | 0,059 | *QMLNR_6_SB_GK_4800* | 220 | 0,937 | 0,003 | 0,398 | 0,130 | 0,109 | *QMLNR_6_FR_GK_4800* | 220 | 0,900 | 0,002 | 0,323 | 0,191 | 0,132 |
| *QMLNR_6_RD_GK_9600* | 220 | 0,971 | 0,001 | 0,531 | 0,084 | 0,077 | *QMLNR_6_SB_GK_9600* | 220 | 0,934 | 0,004 | 0,465 | 0,153 | 0,100 | *QMLNR_6_FR_GK_9600* | 220 | 0,900 | 0,002 | 0,383 | 0,193 | 0,127 |
|  |  |  |  |  |  |  |  |  |  |  |  |  |  |  |  |  |  |  |  |  |
| **Average** | **N** | **AUC** | **AUC_diff_** | **MTSS** | **Area** | **MTSS _om_** | **Average** | **N** | **AUC** | **AUC_diff_** | **MTSS** | **Area** | **MTSS _om_** | **Average** | **N** | **AUC** | **AUC_diff_** | **MTSS** | **Area** | **MTSS _om_** |
| **QMLNR_RD_RAW** | 220 | 0,972 | 0,001 | 0,194 | 0,064 | 0,085 | **QMLNR_SB_RAW** | 220 | 0,933 | 0,003 | 0,215 | 0,150 | 0,096 | **QMLNR_FR_RAW** | 220 | 0,910 | 0,003 | 0,227 | 0,177 | 0,142 |
| *QMLNR_RD_SR_1200* | 79 | 0,958 | 0,005 | 0,186 | 0,106 | 0,129 | *QMLNR_SB_SR_1200* | 182 | 0,929 | 0,003 | 0,237 | 0,148 | 0,112 | *QMLNR_FR_SR_1200* | 207 | 0,905 | 0,004 | 0,234 | 0,184 | 0,141 |
| *QMLNR_RD_SR_2400* | 49 | 0,942 | 0,009 | 0,207 | 0,138 | 0,125 | *QMLNR_SB_SR_2400* | 134 | 0,914 | 0,005 | 0,264 | 0,170 | 0,121 | *QMLNR_FR_SR_2400* | 173 | 0,896 | 0,005 | 0,259 | 0,197 | 0,150 |
| *QMLNR_RD_SR_4800* | 28 | 0,905 | 0,015 | 0,331 | 0,170 | 0,190 | *QMLNR_SB_SR_4800* | 80 | 0,882 | 0,013 | 0,304 | 0,214 | 0,160 | *QMLNR_FR_SR_4800* | 131 | 0,871 | 0,009 | 0,310 | 0,220 | 0,173 |
| *QMLNR_RD_SR_9600* | 19 | 0,904 | 0,015 | 0,316 | 0,202 | 0,150 | *QMLNR_SB_SR_9600* | 41 | 0,863 | 0,021 | 0,373 | 0,212 | 0,199 | *QMLNR_FR_SR_9600* | 82 | 0,856 | 0,008 | 0,344 | 0,242 | 0,184 |
| *QMLNR_RD_GK_1200* | 220 | 0,971 | 0,001 | 0,263 | 0,065 | 0,085 | *QMLNR_SB_GK_1200* | 220 | 0,933 | 0,003 | 0,239 | 0,151 | 0,095 | *QMLNR_FR_GK_1200* | 220 | 0,910 | 0,003 | 0,247 | 0,176 | 0,141 |
| *QMLNR_RD_GK_2400* | 220 | 0,971 | 0,001 | 0,321 | 0,069 | 0,085 | *QMLNR_SB_GK_2400* | 220 | 0,933 | 0,003 | 0,276 | 0,151 | 0,096 | *QMLNR_FR_GK_2400* | 220 | 0,910 | 0,003 | 0,264 | 0,177 | 0,141 |
| *QMLNR_RD_GK_4800* | 220 | 0,971 | 0,002 | 0,432 | 0,072 | 0,082 | *QMLNR_SB_GK_4800* | 220 | 0,933 | 0,003 | 0,355 | 0,155 | 0,099 | *QMLNR_FR_GK_4800* | 220 | 0,911 | 0,003 | 0,308 | 0,178 | 0,136 |
| *QMLNR_RD_GK_9600* | 220 | 0,968 | 0,002 | 0,501 | 0,093 | 0,081 | *QMLNR_SB_GK_9600* | 220 | 0,929 | 0,004 | 0,439 | 0,166 | 0,104 | *QMLNR_FR_GK_9600* | 220 | 0,910 | 0,003 | 0,375 | 0,192 | 0,125 |

**Appendix 4, Table 3. Performance of spatial rarefaction (SR) and Gaussian density kernel (GK) at four different radii, reported for each of the five top models and as average values, across three datasets representing different bias intensities (RD=real dataset; SB= simulated biased; FR= Full random), in Qomolangma National Nature Reserve. MTSS=Maximum training sensitivity plus specificity logistic threshold; MTSS om= omission rate for MTSS threshold; Area= MTSS Area.**

**References**

Acevedo, P., Jiménez-Valverde, A., Lobo, J. M., & Real, R. (2012). Delimiting the geographical background in species distribution modeling. Journal of Biogeography, 39(8), 1383–1390. doi:10.1111/j.1365-2699.2012.02713.x

Beale, C. M., J. J. Lennon, and A. Gimona. 2008. Opening the climate envelope reveals no macroscale associations with climate in European birds. Proceedings of the National Academy of Sciences USA 105, 14908–14912.

Boria R, Olson L, Goodman S, Anderson R (2014) Spatial filtering to reduce sampling bias can improve the performance of ecological niche models. Ecol Model 275:73–77. doi: 10.1016/j.ecolmodel.2013.12.012

Chapman, D. S. 2010. Weak climatic associations among British plant distributions. Global Ecology and Biogeography 19, 831–841.

Chefaoui R, Lobo J (2008) Assessing the effects of pseudo-absences on predictive distribution model performance. Ecol Model 210:478–486. doi: 10.1016/j.ecolmodel.2007.08.010

Cushman, S. A., Macdonald, E. A., Landguth, E. L., Malhi, Y., & Macdonald, D. W. (2017). Multiple-scale prediction of forest loss risk across Borneo. *Landscape Ecology*, *32*(8), 1581–1598. https://doi.org/10.1007/s10980-017-0520-0

Cushman, Samuel A. 2014. Grand challenges in evolutionary and population genetics: The importance of integrating epigenetics, genomics, modeling, and experimentation. Frontiers in Genetics. 5: Article 197.

Fourcade Y, Engler JO, Rödder D, Secondi J (2014) Mapping Species Distributions with MAXENT Using a Geographically Biased Sample of Presence Data: A Performance Assessment of Methods for Correcting Sampling Bias. PLoS ONE 9:e97122. doi: 10.1371/journal.pone.0097122

Hijmans R (2012) Cross‐validation of species distribution models: removing spatial sorting bias and calibration with a null model. Ecology 93:679–688. doi: 10.1890/11-0826.1

Jimenez-Valverde A (2012). Insights into the area under the receiver operating characteristic curve (AUC) as a discrimination measure in species distribution modelling. Global Ecology and Biogeogrpahy, 21, 498–507.

Kramer‐Schadt S, Niedballa J, Pilgrim JD, et al (2013) The importance of correcting for sampling bias in MaxEnt species distribution models. Diversity and Distributions 19:1366–1379. doi: 10.1111/ddi.12096

Liu C, White M, Newell G (2013) Selecting thresholds for the prediction of species occurrence with presence‐only data. J Biogeogr 40:778–789. doi: 10.1111/jbi.12058

Lobo JM, Jimenez-Valverde A, Real R (2008) AUC: a misleading measure of the performance of predictive distribution models. Glob Ecol Biogeogr 17: 145–151.

Merckx, B., M. Steyaert, A. Vanreusel, M. Vincx, and J. Vanaverbeke. 2011. Null models reveal preferential sampling, spatial autocorrelation and overfitting in habitat suitability modelling. Ecological Modelling 222, 588–597.

Phillips SJ, Anderson RP, Schapire RE (2006) Maximum entropy modeling of species geographic distributions. Ecological modeling 190:231–259

Radosavljevic, A., & Anderson, R. P. (2014). Making better Maxent models of species distributions: complexity, overfitting and evaluation. *Journal of Biogeography*, *41*(4), 629–643. https://doi.org/10.1111/jbi.12227

Raes, N. & ter Steege, H. (2007) A null-model for significance testing of presence-only species distribution models. Ecography, 30, 727–736.

Schoener, T. W. 1968. Anolis lizards of Bimini: resource partitioning in a complex fauna. Ecology 49:704–726.

Shcheglovitova M, Anderson R (2013) Estimating optimal complexity for ecological niche models: A jackknife approach for species with small sample sizes. Ecol Model 269:9–17. doi: 10.1016/j.ecolmodel.2013.08.011

Syfert MM, Smith MJ, Coomes DA (2013) The effects of sampling bias and model complexity on the predictive performance of MaxEnt species distribution models. PLoS ONE 8:e55158. doi: 10.1371/journal.pone.0055158

Varela S, Anderson RP, García‐Valdés R, Fernández‐González F (2014) Environmental filters reduce the effects of sampling bias and improve predictions of ecological niche models. Ecography 37:1084–1091. doi: 10.1111/j.1600-0587.2013.00441.x

Veloz SD (2009) Spatially autocorrelated sampling falsely inflates measures of accuracy for presence‐only niche models. Journal of Biogeography 36:2290–2299. doi: 10.1111/j.1365-2699.2009.02174.x

Vergara M, Cushman S, Urra F, Ruiz-González A (2015) Shaken but not stirred: multiscale habitat suitability modeling of sympatric marten species (Martes martes and Martes foina) in the northern Iberian Peninsula. Landscape Ecol 31:1241–1260. doi: 10.1007/s10980-015-0307-0

Warren DL, Seifert SN (2011). Ecological niche modeling in Maxent: the importance of model complexity and the performance of model selection criteria. Ecol Appl 21:335–342
