# Supplementary material for: Meta‐replication, sampling bias, and multi‐scale model selection: A case study on snow leopard (Panthera uncia) in western China
Source: Ecol Evol. 2020 Jul 6;10(14):7686–712. doi: 10.1002/ece3.6492 (PMC7391562; doi:10.1002/ece3.6492)
Supplement: Supplementary file 5 — Tables S1–S6 [file ECE3-10-7686-s005.docx]

|  | **QMLNR** | **QLSNP** |
| --- | --- | --- |
| **Scats** | 464 | 475 |
| **Cytb+** | 221 | 259 |
| **ATP6+** | 96 | 109 |
| **ID+** | 317 | 368 |
| **Failed** | 147 | 107 |
| **SL** | 134 | 230 |
| **W** | 87 | 36 |
| **L** | 56 | 48 |
| **RF** | 34 | 38 |
| **OC** | 6 | 16 |
| **NC** | 10 | 8 |

Supplementary Table S1. Results of species identification from faecal samples in Qomolangma National Nature Reserve (QMLNR) and Qilianshan National Park (QLSNP). Cytb+=successful identifications using cythocrome-b gene portion; ATP6+=successful identifications using ATP6 gene portion; ID+=total number of successful identifications; SL= Snow leopard (Panthera uncia); W= Wolf (Canis lupus); L= Lynx (Lynx lynx); RF=Red fox (Vulpes vulpes); OC=other carnivores; NC=non-carnivore species identifications. See Bai et al. (2018) for details on laboratory methods.

| **QLSNP** |  |  |  |  |  |  |  |  |  |  |  |  |  |  |  |  |  |  |  |  |  |
| --- | --- | --- | --- | --- | --- | --- | --- | --- | --- | --- | --- | --- | --- | --- | --- | --- | --- | --- | --- | --- | --- |
|  |  |  | **Scales** | | | | | | | | | | | | | | | | | | |
| **Category** | **Variables** | **Class** | **300** | | **600** | | **1200** | | **2400** | | **4800** | | **9600** | | **14400** | | **19200** | | **28800** | | |
|  |  |  | test AUC | train AUC | test AUC | train AUC | test AUC | train AUC | test AUC | train AUC | test AUC | train AUC | test AUC | train AUC | test AUC | train AUC | test AUC | train AUC | test AUC | train AUC |  |
| **Linear and Point features** | Dens_riv |  | 0,592 | 0,561 | 0,612 | 0,559 | 0,626 | 0,561 | 0,623 | 0,563 | **0,627** | **0,564** | 0,512 | 0,539 | 0,555 | 0,582 | 0,566 | 0,574 | 0,581 | 0,569 |  |
|  | Dens_rd |  | 0,499 | 0,503 | 0,478 | 0,505 | 0,491 | 0,515 | 0,505 | 0,536 | 0,541 | 572 | 0,562 | 0,586 | 0,556 | 0,571 | **0,613** | **0,642** | 0,582 | 0,593 |  |
|  | Dens_set |  | 0,5 | 0,5 | 0,5 | 0,5 | 0,5 | 0,5 | 0,505 | 0,505 | 0,511 | 0,515 | 0,503 | 0,513 | 0,5 | 0,499 | 0,566 | 0,593 | **0,71** | **0,667** |  |
| **Topography** | CTI |  | 0,691 | 0,653 | 0,72 | 0,655 | 0,632 | 0,642 | 0,678 | 0,671 | 0,719 | 0,702 | **0,741** | **0,724** | 0,729 | 0,706 | 0,701 | 0,674 | 0,65 | 0,621 |  |
|  | SLP |  | 0,722 | 0,716 | 0,745 | 0,739 | 0,759 | 0,751 | **0,801** | **0,759** | 0,794 | 0,742 | 0,777 | 0,715 | 0,746 | 0,676 | 0,71 | 0,641 | 0,685 | 0,625 |  |
|  | ROUGH |  | 0,678 | 0,686 | **0,717** | **0,678** | 0,685 | 0,676 | 0,663 | 0,668 | 0,669 | 0,684 | 0,617 | 0,633 | 0,601 | 0,635 | 0,595 | 0,633 | 0,548 | 0,594 |  |
|  | DISS |  | 0,745 | 0,742 | **0,756** | **0,753** | 0,755 | 0,744 | 0,737 | 0,694 | 0,652 | 0,587 | 0,64 | 0,623 | 0,658 | 0,67 | 0,67 | 0,683 | 0,676 | 0,663 |  |
|  | ELEV |  | 0,67 | 0,666 | 0,673 | 0,676 | 0,681 | 0,689 | 0,689 | 0,692 | 0,69 | 0,696 | 0,666 | 0,68 | 0,686 | 0,691 | **0,722** | **0,73** | 0,697 | 0,702 |  |
| **Climatic** | TEMP |  | **0,732** | **0,74** | 0,73 | 0,742 | 0,73 | 0,739 | 0,729 | 0,733 | 0,717 | 0,721 | 0,666 | 0,693 | 0,679 | 0,698 | 0,73 | 0,725 | 0,693 | 0,695 |  |
| **Landscape level** | CWED |  | 0,559 | 0,578 | 0,587 | 0,597 | 0,615 | 0,633 | 0,636 | 0,624 | **0,647** | **0,637** | 0,601 | 0,58 | 0,518 | 0,505 | 0,461 | 0,487 | 0,41 | 0,421 |  |
|  | AI |  | 0,548 | 0,581 | 0,575 | 0,585 | 0,619 | 0,609 | 0,648 | 0,61 | 0,64 | 0,627 | 0,656 | 0,64 | 0,644 | 0,63 | 0,654 | 0,651 | **0,675** | **0,707** |  |
|  | PD |  | 0,545 | 0,582 | 0,61 | 0,61 | 0,605 | 0,612 | 0,59 | 0,593 | 0,644 | 0,596 | 0,642 | 0,63 | 0,606 | 0,612 | 0,623 | 0,636 | **0,673** | **0,633** |  |
| **Class level** | PLAND | Br | 0,585 | 0,655 | 0,58 | 0,635 | 0,596 | 0,625 | 0,596 | 0,635 | 0,592 | 0,651 | 0,598 | 0,651 | **0,631** | **0,677** | 0,598 | 0,641 | 0,552 | 0,59 |  |
|  | AREA_AM |  | 0,583 | 0,62 | 0,581 | 0,633 | 0,59 | 0,622 | 0,619 | 0,653 | 0,657 | 0,695 | **0,669** | **0,706** | 0,634 | 0,67 | 0,597 | 0,639 | 0,534 | 0,587 |  |
|  | GYR_AM |  | 0,594 | 0,624 | 0,587 | 0,639 | 0,617 | 0,646 | 0,604 | 0,643 | 0,608 | 0,658 | **0,639** | **0,685** | 0,62 | 0,662 | 0,596 | 0,651 | 0,561 | 0,634 |  |
|  | PLAND | Sn | 0,549 | 0,553 | 0,566 | 0,566 | 0,6 | 0,59 | 0,62 | 0,612 | 0,612 | 0,609 | **0,626** | **0,659** | 0,553 | 0,587 | 0,494 | 0,505 | 0,562 | 0,56 |  |
|  | AREA_AM |  | 0,549 | 0,553 | 0,566 | 0,566 | 0,6 | 0,59 | 0,62 | 0,611 | 0,604 | 0,608 | **0,627** | **0,624** | 0,585 | 0,606 | 0,485 | 0,519 | 0,467 | 0,456 |  |
|  | GYR_AM |  | 0,549 | 0,553 | 0,566 | 0,567 | 0,6 | 0,59 | 0,609 | 0,606 | 0,621 | 0,602 | **0,629** | **0,674** | 0,56 | 0,592 | 0,517 | 0,557 | 0,588 | 0,624 |  |
|  | PLAND | Gr | 0,532 | 0,574 | 0,529 | 0,603 | 0,602 | 0,575 | 0,557 | 0,528 | 0,526 | 0,522 | 0,57 | 0,559 | **0,686** | **0,669** | 0,604 | 0,595 | 0,577 | 0,557 |  |
|  | AREA_AM |  | 0,536 | 0,572 | 0,533 | 0,605 | 0,617 | 0,582 | **0,628** | **0,614** | 0,622 | 0,595 | 0,614 | 0,612 | 0,58 | 0,59 | 0,563 | 0,587 | 0,576 | 0,529 |  |
|  | GYR_AM |  | 0,539 | 0,576 | 0,538 | 0,605 | 0,615 | 0,58 | 0,608 | 0,612 | **0,638** | **0,588** | 0,63 | 0,611 | 0,611 | 0,6 | 0,6 | 0,583 | 0,55 | 0,518 |  |
|  | PLAND | Shr | 0,502 | 0,5 | 0,5 | 0,506 | 0,517 | 0,521 | 0,544 | 0,537 | 0,55 | 0,577 | 0,562 | 0,591 | 0,585 | 0,623 | **0,595** | **0,604** | 0,589 | 0,614 |  |
|  | AREA_AM |  | 0,502 | 0,5 | 0,5 | 0,506 | 0,516 | 0,521 | 0,543 | 0,538 | 0,578 | 0,55 | 0,572 | 0,608 | 0,594 | 0,626 | **0,613** | **0,619** | 0,587 | 0,63 |  |
|  | GYR_AM |  | 0,502 | 0,5 | 0,499 | 0,506 | 0,517 | 0,521 | 0,541 | 0,537 | 0,551 | 0,578 | 0,574 | 0,609 | 0,592 | 0,625 | **0,608** | **0,618** | 0,591 | 0,628 |  |
|  | PLAND | NLF | 0,536 | 0,504 | 0,541 | 0,505 | 0,545 | 0,513 | 0,49 | 0,507 | 0,483 | 0,519 | 0,525 | 0,571 | 0,565 | 0,613 | **0,568** | **0,625** | 0,549 | 0,627 |  |
|  | AREA_AM |  | 0,526 | 0,505 | 0,541 | 0,505 | 0,546 | 0,514 | 0,49 | 0,506 | 0,496 | 0,522 | 0,535 | 0,565 | 0,563 | 0,602 | 0,571 | 0,617 | **0,573** | **0,633** |  |
|  | GYR_AM |  | 0,536 | 0,504 | 0,541 | 0,505 | 0,516 | 0,513 | 0,509 | 0,514 | 0,495 | 0,521 | 0,533 | 0,564 | 0,562 | 0,602 | 0,57 | 0,618 | **0,574** | **0,633** |  |
| **QMLNR** | |  |  |  |  |  |  |  |  |  |  |  |  |  |  |  |  |  |  |  |  |
|  |  |  | **Scales** | | | | | | | | | | | | | | | |  |  | |
| **Category** | **Variables** | **Class** | **300** | | **600** | | **1200** | | **2400** | | **4800** | | **9600** | | **14400** | | **19200** | | **28800** | | |
|  |  |  | test AUC | train AUC | test AUC | train AUC | test AUC | train AUC | test AUC | train AUC | test AUC | train AUC | test AUC | train AUC | test AUC | train AUC | test AUC | train AUC | test AUC | train AUC |  |
| **Linear and Point features** | Dens_riv |  | 0,531 | 0,548 | 0,554 | 0,565 | 0,575 | 0,61 | 0,606 | 0,652 | 0,659 | 0,718 | 0,738 | 0,732 | 0,772 | 0,752 | **0,794** | 0,795 | 0,772 | 0,789 |  |
|  | Dens_rd |  | 0,493 | 0,505 | 0,496 | 0,508 | 0,502 | 0,514 | 0,515 | 0,528 | 0,531 | 0,551 | 0,492 | 0,54 | 0,58 | 0,623 | 0,695 | 0,719 | **0,748** | 0,771 |  |
|  | Dens_settl | | 0,5 | 0,5 | 0,506 | 0,506 | 0,508 | 0,504 | 0,617 | 0,582 | 0,723 | 0,712 | **0,769** | 0,76 | 0,739 | 0,724 | 0,683 | 0,662 | 0,738 | 0,737 |  |
| **Topography** | CTI |  | 0,546 | 0,557 | 0,583 | 0,597 | 0,595 | 0,652 | 0,721 | 0,752 | 0,68 | 0,715 | 0,714 | 0,74 | **0,726** | 0,733 | 0,695 | 0,693 | 0,556 | 0,545 |  |
|  | SLP |  | 0,514 | 0,586 | 0,573 | 0,603 | 0,586 | 0,638 | 0,626 | 0,671 | 0,646 | 0,702 | 0,754 | 0,778 | 0,805 | 0,818 | 0,83 | 0,836 | **0,839** | 0,842 |  |
|  | ROUGH |  | 0,652 | 0,656 | 0,613 | 0,664 | 0,602 | 0,701 | 0,587 | 0,656 | 0,593 | 0,626 | 0,663 | 0,68 | 0,728 | 0,753 | **0,78** | 0,789 | 0,705 | 0,712 |  |
|  | DISS |  | 0,627 | 0,655 | 0,606 | 0,592 | 0,514 | 0,584 | 0,595 | 0,629 | 0,678 | 0,701 | 0,774 | 0,811 | **0,826** | 0,837 | 0,813 | 0,829 | 0,796 | 0,813 |  |
|  | ELEV |  | 0,815 | 0,823 | 0,816 | 0,822 | **0,819** | 0,813 | 0,81 | 0,802 | 0,804 | 0,788 | 0,749 | 0,746 | 0,726 | 0,727 | 0,759 | 0,773 | 0,726 | 0,762 |  |
| **Climatic** | TEMP |  | **0,843** | **0,852** | 0,842 | 0,853 | 0,837 | 0,848 | 0,832 | 0,837 | 0,832 | 0,829 | 0,753 | 0,755 | 0,705 | 0,729 | 0,794 | 0,82 | 0,843 | 0,861 |  |
| **Landscape level** | CWED |  | 0,604 | 0,6 | 0,58 | 0,587 | 0,569 | 0,576 | 0,589 | 0,554 | 0,587 | 0,548 | 0,625 | 0,64 | 0,79 | 0,779 | 0,844 | 0,823 | **0,856** | 0,846 |  |
|  | AI |  | 0,583 | 0,542 | 0,559 | 0,576 | 0,588 | 0,606 | 0,625 | 0,635 | 0,586 | 0,578 | 0,614 | 0,596 | 0,774 | 0,741 | **0,776** | 0,756 | 0,754 | 0,77 |  |
|  | PD |  | 0,614 | 0,57 | 0,564 | 0,587 | 0,605 | 0,618 | 0,631 | 0,635 | 0,578 | 0,587 | 0,74 | 0,727 | 0,808 | 0,789 | **0,819** | 0,805 | 0,772 | 0,785 |  |
| **Class level** | PLAND | Br | 0,645 | 0,637 | 0,658 | 0,65 | 0,654 | 0,649 | 0,662 | 0,668 | 0,663 | 0,667 | 0,634 | 0,63 | 0,773 | 0,783 | 0,83 | 0,834 | **0,873** | 0,868 |  |
|  | AREA |  | 0,642 | 0,637 | 0,659 | 0,649 | 0,652 | 0,649 | 0,666 | 0,729 | 0,672 | 0,723 | 0,686 | 0,693 | 0,748 | 0,749 | 0,838 | 0,831 | **0,878** | 0,854 |  |
|  | GYR |  | 0,623 | 0,642 | 0,658 | 0,639 | 0,662 | 0,649 | 0,671 | 0,707 | 0,684 | 0,756 | 0,758 | 0,77 | 0,803 | 0,805 | 0,871 | 0,863 | **0,908** | 0,887 |  |
|  | PLAND | Sn | 0,631 | 0,616 | 0,636 | 0,622 | 0,633 | 0,637 | 0,661 | 0,629 | 0,635 | 0,618 | **0,715** | 0,721 | 0,623 | 0,608 | 0,541 | 0,542 | 0,604 | 0,605 |  |
|  | AREA |  | 0,631 | 0,608 | 0,636 | 0,622 | 0,628 | 0,639 | 0,669 | 0,629 | 0,653 | 0,66 | 0,69 | 0,689 | 0,702 | 0,714 | 0,748 | 0,764 | **0,796** | 0,812 |  |
|  | GYR |  | 0,631 | 0,616 | 0,635 | 0,622 | 0,629 | 0,634 | 0,634 | 0,617 | 0,619 | 0,637 | 0,697 | 0,694 | 0,683 | 0,695 | 0,779 | 0,786 | **0,788** | 0,803 |  |
|  | PLAND | Gr | 0,618 | 0,651 | 0,6 | 0,652 | 0,529 | 0,61 | 0,532 | 0,614 | 0,582 | 0,635 | 0,769 | 0,767 | **0,819** | 0,827 | 0,787 | 0,792 | 0,66 | 0,673 |  |
|  | AREA |  | 0,616 | 0,651 | 0,609 | 0,656 | 0,639 | 0,639 | 0,579 | 0,656 | 0,65 | 0,712 | 0,746 | 0,769 | 0,779 | 0,777 | **0,807** | 0,817 | 0,757 | 0,775 |  |
|  | GYR |  | 0,614 | 0,658 | 0,62 | 0,658 | 0,578 | 0,631 | 0,536 | 0,657 | 0,589 | 0,644 | 0,692 | 0,72 | 0,775 | 0,779 | **0,823** | 0,817 | 0,791 | 0,806 |  |
|  | PLAND | Shr | 0,53 | 0,532 | 0,548 | 0,544 | 0,579 | 0,575 | 0,609 | 0,607 | 0,64 | 0,618 | 0,635 | 0,633 | **0,759** | 0,748 | 0,731 | 0,724 | 0,664 | 0,667 |  |
|  | AREA |  | 0,531 | 0,531 | 0,548 | 0,544 | 0,579 | 0,575 | 0,607 | 0,607 | 0,635 | 0,617 | 0,628 | 0,621 | 0,642 | 0,626 | 0,636 | 0,632 | **0,827** | 0,778 |  |
|  | GYR |  | 0,53 | 0,531 | 0,548 | 0,545 | 0,579 | 0,574 | 0,607 | 0,608 | 0,636 | 0,619 | 0,642 | 0,628 | 0,685 | 0,666 | 0,67 | 0,669 | **0,826** | 0,793 |  |
|  | PLAND | NLF | 0,528 | 0,534 | 0,547 | 0,534 | 0,52 | 0,544 | 0,515 | 0,531 | 0,506 | 0,541 | 0,654 | 0,675 | **0,754** | 0,743 | 0,754 | 0,751 | 0,633 | 0,64 |  |
|  | AREA |  | 0,534 | 0,528 | 0,547 | 0,534 | 0,52 | 0,544 | 0,523 | 0,539 | 0,559 | 0,517 | 0,685 | 0,696 | 0,734 | 0,718 | **0,767** | 0,758 | 0,528 | 0,534 |  |
|  | GYR |  | 0,531 | 0,528 | 0,548 | 0,535 | 0,52 | 0,544 | 0,567 | 0,531 | 0,607 | 0,549 | 0,691 | 0,718 | **0,808** | 0,802 | 0,629 | 0,619 | 0,76 | 0,781 |  |

Supplementary Table S2. AUC values for each of the 27 variables included in this study at the nine scales considered (in meters). Values in bold represent the best scale for a given predictor. QLSNP=Qilian Shan National Park; QMLNR= Qomolangma National Nature Reserve.

| **QLSNP** |  |  |  |  |  |  |  |  |
| --- | --- | --- | --- | --- | --- | --- | --- | --- |
| **Model number** | **Linear/point features** | **Topographic** | **Climatic** | **Landcover Landscape-level** | **Landcover Class-level** | **AUC train** | **AUC test** | **AUC_diff_** |
| **QLSNP_1** | **DENS_set_28800** | **SLP_2400** | **TEMP_300** | **AI_28800** | **GYR_AM_Gr_4800** | **0,865** | **0,892** | **-0,027** |
| **QLSNP_2** | **DENS_set_28800** | **SLP_2400** | **TEMP_300** | **AI_28800** | **PLAND_Gr_14400** | **0,872** | **0,89** | **-0,018** |
| **QLSNP_3** | **DENS_riv_4800** | **SLP_2400** | **TEMP_300** | **AI_28800** | **GYR_AM_Gr_4800** | **0,865** | **0,887** | **-0,022** |
| **QLSNP_4** | **DENS_riv_4800** | **SLP_2400** | **TEMP_300** | **AI_28800** | **PLAND_Gr_14400** | **0,875** | **0,886** | **-0,011** |
| **QLSNP_5** | **DENS_set_28800** | **SLP_2400** | **TEMP_300** | **PD_28800** | **GYR_AM_Gr_4800** | **0,848** | **0,881** | **-0,033** |
| **QLSNP_6** | **DENS_rd_19200** | **SLP_2400** | **TEMP_300** | **AI_28800** | **PLAND_Gr_14400** | **0,88** | **0,879** | **0,001** |
| **QLSNP_7** | **DENS_rd_19200** | **SLP_2400** | **TEMP_300** | **AI_28800** | **GYR_AM_Gr_4800** | **0,873** | **0,878** | **-0,005** |
| **QLSNP_8** | **DENS_set_28800** | **DISS_600** | **TEMP_300** | **AI_28800** | **GYR_AM_Gr_4800** | **0,857** | **0,877** | **-0,020** |
| **QLSNP_9** | **DENS_set_28800** | **DISS_600** | **TEMP_300** | **AI_28800** | **PLAND_Gr_14400** | **0,859** | **0,875** | **-0,016** |
| **QLSNP_10** | **DENS_set_28800** | **SLP_2400** | **TEMP_300** | **AI_28800** | **AREA_AM_Shr_19200** | **0,86** | **0,875** | **-0,015** |
| QLSNP_11 | DENS_set_28800 | ROUGH_600 | TEMP_300 | AI_28800 | GYR_AM_Gr_4800 | 0,853 | 0,874 | -0,021 |
| QLSNP_12 | DENS_set_28800 | SLP_2400 | TEMP_300 | AI_28800 | GYR_AM_Sn_9600 | 0,877 | 0,873 | 0,004 |
| QLSNP_13 | DENS_riv_4800 | SLP_2400 | TEMP_300 | PD_28800 | GYR_AM_Gr_4800 | 0,844 | 0,871 | -0,027 |
| QLSNP_14 | DENS_set_28800 | CTI_9600 | TEMP_300 | PD_28800 | GYR_AM_Gr_4800 | 0,864 | 0,871 | -0,007 |
| QLSNP_15 | DENS_riv_4800 | DISS_600 | TEMP_300 | AI_28800 | GYR_AM_Gr_4800 | 0,856 | 0,87 | -0,014 |
| QLSNP_16 | DENS_set_28800 | CTI_9600 | TEMP_300 | AI_28800 | GYR_AM_Gr_4800 | 0,862 | 0,87 | -0,008 |
| QLSNP_17 | DENS_riv_4800 | SLP_2400 | TEMP_300 | AI_28800 | AREA_AM_Shr_19200 | 0,863 | 0,87 | -0,007 |
| QLSNP_18 | DENS_rd_19200 | DISS_600 | TEMP_300 | AI_28800 | GYR_AM_Gr_4800 | 0,867 | 0,87 | -0,003 |
| QLSNP_19 | DENS_set_28800 | SLP_2400 | TEMP_300 | PD_28800 | PLAND_Gr_14400 | 0,844 | 0,869 | -0,025 |
| QLSNP_20 | DENS_set_28800 | ROUGH_600 | TEMP_300 | AI_28800 | PLAND_Gr_14400 | 0,848 | 0,869 | -0,021 |
| QLSNP_21 | DENS_set_28800 | CTI_9600 | TEMP_300 | PD_28800 | AREA_AM_Shr_19200 | 0,863 | 0,869 | -0,006 |
| QLSNP_22 | DENS_riv_4800 | DISS_600 | TEMP_300 | AI_28800 | PLAND_Gr_14400 | 0,858 | 0,868 | -0,010 |
| QLSNP_23 | DENS_set_28800 | CTI_9600 | TEMP_300 | AI_28800 | AREA_AM_Shr_19200 | 0,864 | 0,868 | -0,004 |
| QLSNP_24 | DENS_rd_19200 | DISS_600 | TEMP_300 | AI_28800 | PLAND_Gr_14400 | 0,869 | 0,868 | 0,001 |
| QLSNP_25 | DENS_set_28800 | DISS_600 | TEMP_300 | PD_28800 | GYR_AM_Gr_4800 | 0,839 | 0,867 | -0,028 |
| QLSNP_26 | DENS_set_28800 | SLP_2400 | TEMP_300 | PD_28800 | AREA_AM_Shr_19200 | 0,849 | 0,867 | -0,018 |
| QLSNP_27 | DENS_riv_4800 | SLP_2400 | TEMP_300 | AI_28800 | GYR_AM_Sn_9600 | 0,877 | 0,867 | 0,010 |
| QLSNP_28 | DENS_set_28800 | ROUGH_600 | TEMP_300 | PD_28800 | GYR_AM_Gr_4800 | 0,837 | 0,866 | -0,029 |
| QLSNP_29 | DENS_riv_4800 | CTI_9600 | TEMP_300 | PD_28800 | AREA_AM_Br_9600 | 0,872 | 0,866 | 0,006 |
| QLSNP_30 | DENS_rd_19200 | CTI_9600 | TEMP_300 | PD_28800 | AREA_AM_Br_9600 | 0,879 | 0,866 | 0,013 |
| QLSNP_31 | DENS_set_28800 | CTI_9600 | TEMP_300 | PD_28800 | PLAND_Gr_14400 | 0,863 | 0,865 | -0,002 |
| QLSNP_32 | DENS_set_28800 | CTI_9600 | TEMP_300 | PD_28800 | AREA_AM_Br_9600 | 0,872 | 0,865 | 0,007 |
| QLSNP_33 | DENS_riv_4800 | CTI_9600 | TEMP_300 | AI_28800 | AREA_AM_Br_9600 | 0,872 | 0,865 | 0,007 |
| QLSNP_34 | DENS_set_28800 | DISS_600 | TEMP_300 | AI_28800 | AREA_AM_Shr_19200 | 0,848 | 0,864 | -0,016 |
| QLSNP_35 | DENS_set_28800 | CTI_9600 | TEMP_300 | AI_28800 | AREA_AM_Br_9600 | 0,871 | 0,864 | 0,007 |
| QLSNP_36 | DENS_rd_19200 | CTI_9600 | TEMP_300 | AI_28800 | AREA_AM_Br_9600 | 0,878 | 0,864 | 0,014 |
| QLSNP_37 | DENS_set_28800 | ROUGH_600 | TEMP_300 | PD_28800 | AREA_AM_Shr_19200 | 0,836 | 0,863 | -0,027 |
| QLSNP_38 | DENS_riv_4800 | ROUGH_600 | TEMP_300 | AI_28800 | GYR_AM_Gr_4800 | 0,844 | 0,863 | -0,019 |
| QLSNP_39 | DENS_rd_19200 | SLP_2400 | TEMP_300 | PD_28800 | GYR_AM_Gr_4800 | 0,858 | 0,863 | -0,005 |
| QLSNP_40 | DENS_set_28800 | DISS_600 | TEMP_300 | PD_28800 | PLAND_Gr_14400 | 0,834 | 0,862 | -0,028 |
| QLSNP_41 | DENS_set_28800 | ROUGH_600 | TEMP_300 | AI_28800 | AREA_AM_Shr_19200 | 0,841 | 0,862 | -0,021 |
| QLSNP_42 | DENS_riv_4800 | SLP_2400 | TEMP_300 | PD_28800 | PLAND_Gr_14400 | 0,841 | 0,862 | -0,021 |
| QLSNP_43 | DENS_riv_4800 | ROUGH_600 | TEMP_300 | AI_28800 | PLAND_Gr_14400 | 0,846 | 0,862 | -0,016 |
| QLSNP_44 | DENS_riv_4800 | CTI_9600 | TEMP_300 | AI_28800 | GYR_AM_Gr_4800 | 0,858 | 0,862 | -0,004 |
| QLSNP_45 | DENS_rd_19200 | CTI_9600 | TEMP_300 | PD_28800 | GYR_AM_Gr_4800 | 0,869 | 0,862 | 0,007 |
| QLSNP_46 | DENS_rd_19200 | SLP_2400 | TEMP_300 | AI_28800 | GYR_AM_Sn_9600 | 0,881 | 0,862 | 0,019 |
| QLSNP_47 | DENS_riv_4800 | CTI_9600 | TEMP_300 | PD_28800 | AREA_AM_Shr_19200 | 0,859 | 0,861 | -0,002 |
| QLSNP_48 | DENS_set_28800 | DISS_600 | TEMP_300 | AI_28800 | GYR_AM_Sn_9600 | 0,865 | 0,861 | 0,004 |
| QLSNP_49 | DENS_rd_19200 | SLP_2400 | TEMP_300 | AI_28800 | AREA_AM_Shr_19200 | 0,871 | 0,861 | 0,010 |
| QLSNP_50 | DENS_set_28800 | ROUGH_600 | TEMP_300 | PD_28800 | PLAND_Gr_14400 | 0,832 | 0,86 | -0,028 |
| QLSNP_51 | DENS_riv_4800 | SLP_2400 | TEMP_300 | PD_28800 | AREA_AM_Shr_19200 | 0,849 | 0,86 | -0,011 |
| QLSNP_52 | DENS_riv_4800 | CTI_9600 | TEMP_300 | AI_28800 | AREA_AM_Shr_19200 | 0,861 | 0,86 | 0,001 |
| QLSNP_53 | DENS_rd_19200 | CTI_9600 | TEMP_300 | PD_28800 | AREA_AM_Shr_19200 | 0,87 | 0,86 | 0,010 |
| QLSNP_54 | DENS_set_28800 | CTI_9600 | TEMP_300 | AI_28800 | GYR_AM_NLF_28800 | 0,877 | 0,86 | 0,017 |
| QLSNP_55 | DENS_rd_19200 | SLP_2400 | TEMP_300 | PD_28800 | PLAND_Gr_14400 | 0,857 | 0,859 | -0,002 |
| QLSNP_56 | DENS_set_28800 | CTI_9600 | TEMP_300 | AI_28800 | PLAND_Gr_14400 | 0,857 | 0,859 | -0,002 |
| QLSNP_57 | DENS_set_28800 | SLP_2400 | TEMP_300 | PD_28800 | GYR_AM_Sn_9600 | 0,862 | 0,859 | 0,003 |
| QLSNP_58 | DENS_riv_4800 | CTI_9600 | TEMP_300 | PD_28800 | GYR_AM_Gr_4800 | 0,862 | 0,859 | 0,003 |
| QLSNP_59 | DENS_riv_4800 | CTI_9600 | TEMP_300 | PD_28800 | PLAND_Gr_14400 | 0,858 | 0,858 | 0,000 |
| QLSNP_60 | DENS_set_28800 | CTI_9600 | TEMP_300 | PD_28800 | GYR_AM_Sn_9600 | 0,864 | 0,858 | 0,006 |
| QLSNP_61 | DENS_riv_4800 | CTI_9600 | TEMP_300 | AI_28800 | GYR_AM_NLF_28800 | 0,878 | 0,858 | 0,020 |
| QLSNP_62 | DENS_set_28800 | DISS_600 | TEMP_300 | PD_28800 | AREA_AM_Shr_19200 | 0,838 | 0,857 | -0,019 |
| QLSNP_63 | DENS_riv_4800 | DISS_600 | TEMP_300 | AI_28800 | AREA_AM_Shr_19200 | 0,849 | 0,857 | -0,008 |
| QLSNP_64 | DENS_rd_19200 | DISS_600 | TEMP_300 | AI_28800 | AREA_AM_Shr_19200 | 0,861 | 0,857 | 0,004 |
| QLSNP_65 | DENS_rd_19200 | CTI_9600 | TEMP_300 | AI_28800 | GYR_AM_Gr_4800 | 0,864 | 0,857 | 0,007 |
| QLSNP_66 | DENS_rd_19200 | CTI_9600 | TEMP_300 | PD_28800 | PLAND_Gr_14400 | 0,87 | 0,857 | 0,013 |
| QLSNP_67 | DENS_riv_4800 | DISS_600 | TEMP_300 | PD_28800 | GYR_AM_Gr_4800 | 0,838 | 0,856 | -0,018 |
| QLSNP_68 | DENS_rd_19200 | DISS_600 | TEMP_300 | PD_28800 | GYR_AM_Gr_4800 | 0,852 | 0,856 | -0,004 |
| QLSNP_69 | DENS_rd_19200 | CTI_9600 | TEMP_300 | AI_28800 | AREA_AM_Shr_19200 | 0,868 | 0,856 | 0,012 |
| QLSNP_70 | DENS_rd_19200 | CTI_9600 | TEMP_300 | PD_28800 | PLAND_Br_14400 | 0,877 | 0,856 | 0,021 |
| QLSNP_71 | DENS_rd_19200 | CTI_9600 | TEMP_300 | AI_28800 | GYR_AM_NLF_28800 | 0,885 | 0,856 | 0,029 |
| QLSNP_72 | DENS_set_28800 | CTI_9600 | TEMP_300 | PD_28800 | GYR_AM_NLF_28800 | 0,872 | 0,855 | 0,017 |
| QLSNP_73 | DENS_rd_19200 | CTI_9600 | TEMP_300 | PD_28800 | GYR_AM_Sn_9600 | 0,873 | 0,855 | 0,018 |
| QLSNP_74 | DENS_riv_4800 | DISS_600 | TEMP_300 | PD_28800 | PLAND_Gr_14400 | 0,835 | 0,854 | -0,019 |
| QLSNP_75 | DENS_rd_19200 | SLP_2400 | TEMP_300 | PD_28800 | AREA_AM_Br_9600 | 0,86 | 0,854 | 0,006 |
| QLSNP_76 | DENS_riv_4800 | DISS_600 | TEMP_300 | AI_28800 | GYR_AM_Sn_9600 | 0,865 | 0,854 | 0,011 |
| QLSNP_77 | DENS_rd_19200 | DISS_600 | TEMP_300 | PD_28800 | PLAND_Gr_14400 | 0,851 | 0,853 | -0,002 |
| QLSNP_78 | DENS_set_28800 | ROUGH_600 | TEMP_300 | AI_28800 | GYR_AM_Sn_9600 | 0,855 | 0,853 | 0,002 |
| QLSNP_79 | DENS_riv_4800 | CTI_9600 | TEMP_300 | PD_28800 | PLAND_Br_14400 | 0,87 | 0,853 | 0,017 |
| QLSNP_80 | DENS_riv_4800 | CTI_9600 | TEMP_300 | PD_28800 | GYR_AM_NLF_28800 | 0,872 | 0,853 | 0,019 |
| QLSNP_81 | DENS_rd_19200 | CTI_9600 | TEMP_300 | PD_28800 | GYR_AM_NLF_28800 | 0,881 | 0,853 | 0,028 |
| QLSNP_82 | DENS_riv_4800 | SLP_2400 | TEMP_300 | PD_28800 | AREA_AM_Br_9600 | 0,847 | 0,852 | -0,005 |
| QLSNP_83 | DENS_rd_19200 | DISS_600 | TEMP_300 | PD_28800 | AREA_AM_Br_9600 | 0,851 | 0,852 | -0,001 |
| QLSNP_84 | DENS_riv_4800 | CTI_9600 | TEMP_300 | AI_28800 | PLAND_Gr_14400 | 0,852 | 0,852 | 0,000 |
| QLSNP_85 | DENS_riv_4800 | CTI_9600 | TEMP_300 | PD_28800 | GYR_AM_Sn_9600 | 0,86 | 0,852 | 0,008 |
| QLSNP_86 | DENS_rd_19200 | SLP_2400 | TEMP_300 | PD_28800 | AREA_AM_Shr_19200 | 0,863 | 0,852 | 0,011 |
| QLSNP_87 | DENS_rd_19200 | SLP_2400 | TEMP_300 | AI_28800 | AREA_AM_Br_9600 | 0,866 | 0,852 | 0,014 |
| QLSNP_88 | DENS_set_28800 | CTI_9600 | TEMP_300 | PD_28800 | PLAND_Br_14400 | 0,868 | 0,852 | 0,016 |
| QLSNP_89 | DENS_riv_4800 | ROUGH_600 | TEMP_300 | PD_28800 | GYR_AM_Gr_4800 | 0,825 | 0,851 | -0,026 |
| QLSNP_90 | DENS_riv_4800 | ROUGH_600 | TEMP_300 | AI_28800 | AREA_AM_Shr_19200 | 0,835 | 0,851 | -0,016 |
| QLSNP_91 | DENS_set_28800 | SLP_2400 | TEMP_300 | PD_28800 | AREA_AM_Br_9600 | 0,845 | 0,851 | -0,006 |
| QLSNP_92 | DENS_rd_19200 | ROUGH_600 | TEMP_300 | AI_28800 | GYR_AM_Gr_4800 | 0,853 | 0,851 | 0,002 |
| QLSNP_93 | DENS_set_28800 | CTI_9600 | TEMP_300 | AI_28800 | GYR_AM_Sn_9600 | 0,857 | 0,851 | 0,006 |
| QLSNP_94 | DENS_rd_19200 | DISS_600 | TEMP_300 | AI_28800 | AREA_AM_Br_9600 | 0,859 | 0,851 | 0,008 |
| QLSNP_95 | DENS_rd_19200 | DISS_600 | TEMP_300 | AI_28800 | GYR_AM_Sn_9600 | 0,873 | 0,851 | 0,022 |
| QLSNP_96 | DENS_rd_19200 | CTI_9600 | TEMP_300 | AI_28800 | PLAND_Br_14400 | 0,875 | 0,851 | 0,024 |
| QLSNP_97 | DENS_rd_19200 | ROUGH_600 | TEMP_300 | PD_28800 | GYR_AM_NLF_28800 | 0,853 | 0,85 | 0,003 |
| QLSNP_98 | DENS_rd_19200 | ROUGH_600 | TEMP_300 | AI_28800 | PLAND_Gr_14400 | 0,855 | 0,85 | 0,005 |
| QLSNP_99 | DENS_rd_19200 | CTI_9600 | TEMP_300 | AI_28800 | PLAND_Gr_14400 | 0,861 | 0,85 | 0,011 |
| QLSNP_100 | DENS_set_28800 | CTI_9600 | TEMP_300 | AI_28800 | PLAND_Br_14400 | 0,868 | 0,85 | 0,018 |
| QLSNP_101 | DENS_riv_4800 | CTI_9600 | TEMP_300 | AI_28800 | PLAND_Br_14400 | 0,869 | 0,85 | 0,019 |
| QLSNP_102 | DENS_riv_4800 | ROUGH_600 | TEMP_300 | PD_28800 | AREA_AM_Shr_19200 | 0,827 | 0,849 | -0,022 |
| QLSNP_103 | DENS_riv_4800 | SLP_2400 | TEMP_300 | AI_28800 | AREA_AM_Br_9600 | 0,857 | 0,849 | 0,008 |
| QLSNP_104 | DENS_riv_4800 | SLP_2400 | TEMP_300 | PD_28800 | GYR_AM_Sn_9600 | 0,859 | 0,849 | 0,010 |
| QLSNP_105 | DENS_set_28800 | DISS_600 | TEMP_300 | PD_28800 | AREA_AM_Br_9600 | 0,833 | 0,848 | -0,015 |
| QLSNP_106 | DENS_riv_4800 | DISS_600 | TEMP_300 | PD_28800 | AREA_AM_Br_9600 | 0,836 | 0,848 | -0,012 |
| QLSNP_107 | DENS_set_28800 | DISS_600 | TEMP_300 | PD_28800 | GYR_AM_Sn_9600 | 0,853 | 0,848 | 0,005 |
| QLSNP_108 | DENS_set_28800 | SLP_2400 | TEMP_300 | AI_28800 | AREA_AM_Br_9600 | 0,855 | 0,848 | 0,007 |
| QLSNP_109 | DENS_riv_4800 | ROUGH_600 | TEMP_300 | PD_28800 | PLAND_Gr_14400 | 0,824 | 0,847 | -0,023 |
| QLSNP_110 | DENS_rd_19200 | DISS_600 | TEMP_300 | PD_28800 | AREA_AM_Shr_19200 | 0,851 | 0,847 | 0,004 |
| QLSNP_111 | DENS_rd_19200 | DISS_600 | TEMP_300 | PD_28800 | PLAND_Br_14400 | 0,857 | 0,847 | 0,010 |
| QLSNP_112 | DENS_set_28800 | DISS_600 | TEMP_300 | AI_28800 | AREA_AM_Br_9600 | 0,842 | 0,846 | -0,004 |
| QLSNP_113 | DENS_riv_4800 | DISS_600 | TEMP_300 | AI_28800 | AREA_AM_Br_9600 | 0,846 | 0,846 | 0,000 |
| QLSNP_114 | DENS_riv_4800 | CTI_9600 | TEMP_300 | AI_28800 | GYR_AM_Sn_9600 | 0,853 | 0,846 | 0,007 |
| QLSNP_115 | DENS_rd_19200 | SLP_2400 | TEMP_300 | PD_28800 | PLAND_Br_14400 | 0,863 | 0,846 | 0,017 |
| QLSNP_116 | DENS_riv_4800 | DISS_600 | TEMP_300 | PD_28800 | AREA_AM_Shr_19200 | 0,836 | 0,845 | -0,009 |
| QLSNP_117 | DENS_rd_19200 | DISS_600 | TEMP_300 | AI_28800 | PLAND_Br_14400 | 0,857 | 0,845 | 0,012 |
| QLSNP_118 | DENS_rd_19200 | CTI_9600 | TEMP_300 | AI_28800 | GYR_AM_Sn_9600 | 0,861 | 0,845 | 0,016 |
| QLSNP_119 | DENS_rd_19200 | ROUGH_600 | TEMP_300 | PD_28800 | AREA_AM_Shr_19200 | 0,843 | 0,844 | -0,001 |
| QLSNP_120 | DENS_rd_19200 | ROUGH_600 | TEMP_300 | PD_28800 | GYR_AM_Gr_4800 | 0,843 | 0,844 | -0,001 |
| QLSNP_121 | DENS_set_28800 | ROUGH_600 | TEMP_300 | PD_28800 | GYR_AM_Sn_9600 | 0,844 | 0,844 | 0,000 |
| QLSNP_122 | DENS_riv_4800 | ROUGH_600 | TEMP_300 | AI_28800 | GYR_AM_Sn_9600 | 0,849 | 0,844 | 0,005 |
| QLSNP_123 | DENS_rd_19200 | DISS_600 | TEMP_300 | AI_28800 | GYR_AM_NLF_28800 | 0,863 | 0,844 | 0,019 |
| QLSNP_124 | DENS_rd_19200 | SLP_2400 | TEMP_300 | PD_28800 | GYR_AM_Sn_9600 | 0,871 | 0,844 | 0,027 |
| QLSNP_125 | DENS_rd_19200 | ROUGH_600 | TEMP_300 | PD_28800 | AREA_AM_Br_9600 | 0,849 | 0,843 | 0,006 |
| QLSNP_126 | DENS_rd_19200 | ROUGH_600 | TEMP_300 | PD_28800 | PLAND_Gr_14400 | 0,842 | 0,842 | 0,000 |
| QLSNP_127 | DENS_riv_4800 | SLP_2400 | TEMP_300 | PD_28800 | PLAND_Br_14400 | 0,851 | 0,842 | 0,009 |
| QLSNP_128 | DENS_riv_4800 | DISS_600 | TEMP_300 | PD_28800 | PLAND_Br_14400 | 0,843 | 0,841 | 0,002 |
| QLSNP_129 | DENS_rd_19200 | SLP_2400 | TEMP_300 | AI_28800 | PLAND_Br_14400 | 0,86 | 0,841 | 0,019 |
| QLSNP_130 | DENS_rd_19200 | SLP_2400 | TEMP_300 | AI_28800 | GYR_AM_NLF_28800 | 0,872 | 0,841 | 0,031 |
| QLSNP_131 | DENS_set_28800 | ROUGH_600 | TEMP_300 | PD_28800 | AREA_AM_Br_9600 | 0,834 | 0,84 | -0,006 |
| QLSNP_132 | DENS_rd_19200 | ROUGH_600 | TEMP_300 | AI_28800 | AREA_AM_Shr_19200 | 0,846 | 0,84 | 0,006 |
| QLSNP_133 | DENS_rd_19200 | DISS_600 | TEMP_300 | PD_28800 | GYR_AM_NLF_28800 | 0,855 | 0,84 | 0,015 |
| QLSNP_134 | DENS_set_28800 | DISS_600 | TEMP_300 | PD_28800 | PLAND_Br_14400 | 0,839 | 0,839 | 0,000 |
| QLSNP_135 | DENS_riv_4800 | DISS_600 | TEMP_300 | AI_28800 | PLAND_Br_14400 | 0,842 | 0,839 | 0,003 |
| QLSNP_136 | DENS_set_28800 | DISS_600 | TEMP_300 | AI_28800 | GYR_AM_NLF_28800 | 0,845 | 0,839 | 0,006 |
| QLSNP_137 | DENS_riv_4800 | DISS_600 | TEMP_300 | AI_28800 | GYR_AM_NLF_28800 | 0,85 | 0,839 | 0,011 |
| QLSNP_138 | DENS_riv_4800 | ROUGH_600 | TEMP_300 | PD_28800 | AREA_AM_Br_9600 | 0,831 | 0,838 | -0,007 |
| QLSNP_139 | DENS_set_28800 | DISS_600 | TEMP_300 | AI_28800 | PLAND_Br_14400 | 0,839 | 0,838 | 0,001 |
| QLSNP_140 | DENS_riv_4800 | SLP_2400 | TEMP_300 | AI_28800 | PLAND_Br_14400 | 0,849 | 0,838 | 0,011 |
| QLSNP_141 | DENS_rd_19200 | ROUGH_600 | TEMP_300 | AI_28800 | AREA_AM_Br_9600 | 0,854 | 0,838 | 0,016 |
| QLSNP_142 | DENS_riv_4800 | SLP_2400 | TEMP_300 | AI_28800 | GYR_AM_NLF_28800 | 0,862 | 0,838 | 0,024 |
| QLSNP_143 | DENS_set_28800 | ELEV_19200 | TEMP_300 | AI_28800 | PLAND_Gr_14400 | 0,817 | 0,837 | -0,020 |
| QLSNP_144 | DENS_set_28800 | ROUGH_600 | TEMP_300 | AI_28800 | AREA_AM_Br_9600 | 0,841 | 0,837 | 0,004 |
| QLSNP_145 | DENS_set_28800 | SLP_2400 | TEMP_300 | PD_28800 | PLAND_Br_14400 | 0,847 | 0,837 | 0,010 |
| QLSNP_146 | DENS_riv_4800 | DISS_600 | TEMP_300 | PD_28800 | GYR_AM_Sn_9600 | 0,852 | 0,837 | 0,015 |
| QLSNP_147 | DENS_rd_19200 | SLP_2400 | TEMP_300 | PD_28800 | GYR_AM_NLF_28800 | 0,863 | 0,837 | 0,026 |
| QLSNP_148 | DENS_set_28800 | DISS_600 | TEMP_300 | PD_28800 | GYR_AM_NLF_28800 | 0,839 | 0,836 | 0,003 |
| QLSNP_149 | DENS_rd_19200 | ROUGH_600 | TEMP_300 | PD_28800 | PLAND_Br_14400 | 0,853 | 0,836 | 0,017 |
| QLSNP_150 | DENS_set_28800 | SLP_2400 | TEMP_300 | AI_28800 | GYR_AM_NLF_28800 | 0,858 | 0,836 | 0,022 |
| QLSNP_151 | DENS_rd_19200 | DISS_600 | TEMP_300 | PD_28800 | GYR_AM_Sn_9600 | 0,861 | 0,836 | 0,025 |
| QLSNP_152 | DENS_riv_4800 | ROUGH_600 | TEMP_300 | AI_28800 | AREA_AM_Br_9600 | 0,84 | 0,835 | 0,005 |
| QLSNP_153 | DENS_set_28800 | SLP_2400 | TEMP_300 | AI_28800 | PLAND_Br_14400 | 0,846 | 0,835 | 0,011 |
| QLSNP_154 | DENS_riv_4800 | DISS_600 | TEMP_300 | PD_28800 | GYR_AM_NLF_28800 | 0,842 | 0,834 | 0,008 |
| QLSNP_155 | DENS_set_28800 | SLP_2400 | TEMP_300 | PD_28800 | GYR_AM_NLF_28800 | 0,848 | 0,834 | 0,014 |
| QLSNP_156 | DENS_riv_4800 | SLP_2400 | TEMP_300 | PD_28800 | GYR_AM_NLF_28800 | 0,851 | 0,834 | 0,017 |
| QLSNP_157 | DENS_set_28800 | ELEV_19200 | TEMP_300 | AI_28800 | GYR_AM_Sn_9600 | 0,827 | 0,833 | -0,006 |
| QLSNP_158 | DENS_set_28800 | ROUGH_600 | TEMP_300 | PD_28800 | GYR_AM_NLF_28800 | 0,839 | 0,832 | 0,007 |
| QLSNP_159 | DENS_rd_19200 | ROUGH_600 | TEMP_300 | AI_28800 | GYR_AM_Sn_9600 | 0,855 | 0,832 | 0,023 |
| QLSNP_160 | DENS_rd_19200 | ROUGH_600 | TEMP_300 | AI_28800 | GYR_AM_NLF_28800 | 0,861 | 0,832 | 0,029 |
| QLSNP_161 | DENS_set_28800 | ELEV_19200 | TEMP_300 | PD_28800 | GYR_AM_Gr_4800 | 0,785 | 0,831 | -0,046 |
| QLSNP_162 | DENS_set_28800 | ROUGH_600 | TEMP_300 | PD_28800 | PLAND_Br_14400 | 0,838 | 0,831 | 0,007 |
| QLSNP_163 | DENS_set_28800 | ROUGH_600 | TEMP_300 | AI_28800 | GYR_AM_NLF_28800 | 0,846 | 0,831 | 0,015 |
| QLSNP_164 | DENS_set_28800 | ELEV_19200 | TEMP_300 | AI_28800 | GYR_AM_Gr_4800 | 0,814 | 0,83 | -0,016 |
| QLSNP_165 | DENS_riv_4800 | ELEV_19200 | TEMP_300 | AI_28800 | PLAND_Gr_14400 | 0,818 | 0,83 | -0,012 |
| QLSNP_166 | DENS_riv_4800 | ROUGH_600 | TEMP_300 | PD_28800 | GYR_AM_Sn_9600 | 0,835 | 0,829 | 0,006 |
| QLSNP_167 | DENS_riv_4800 | ROUGH_600 | TEMP_300 | PD_28800 | PLAND_Br_14400 | 0,836 | 0,828 | 0,008 |
| QLSNP_168 | DENS_riv_4800 | ROUGH_600 | TEMP_300 | AI_28800 | GYR_AM_NLF_28800 | 0,846 | 0,828 | 0,018 |
| QLSNP_169 | DENS_rd_19200 | ROUGH_600 | TEMP_300 | AI_28800 | PLAND_Br_14400 | 0,85 | 0,827 | 0,023 |
| QLSNP_170 | DENS_set_28800 | ELEV_19200 | TEMP_300 | PD_28800 | PLAND_Gr_14400 | 0,787 | 0,826 | -0,039 |
| QLSNP_171 | DENS_riv_4800 | ROUGH_600 | TEMP_300 | PD_28800 | GYR_AM_NLF_28800 | 0,835 | 0,826 | 0,009 |
| QLSNP_172 | DENS_set_28800 | ROUGH_600 | TEMP_300 | AI_28800 | PLAND_Br_14400 | 0,837 | 0,825 | 0,012 |
| QLSNP_173 | DENS_rd_19200 | ROUGH_600 | TEMP_300 | PD_28800 | GYR_AM_Sn_9600 | 0,849 | 0,825 | 0,024 |
| QLSNP_174 | DENS_riv_4800 | ELEV_19200 | TEMP_300 | AI_28800 | GYR_AM_Gr_4800 | 0,817 | 0,824 | -0,007 |
| QLSNP_175 | DENS_riv_4800 | ROUGH_600 | TEMP_300 | AI_28800 | PLAND_Br_14400 | 0,835 | 0,824 | 0,011 |
| QLSNP_176 | DENS_set_28800 | ELEV_19200 | TEMP_300 | PD_28800 | GYR_AM_Sn_9600 | 0,809 | 0,821 | -0,012 |
| QLSNP_177 | DENS_riv_4800 | ELEV_19200 | TEMP_300 | AI_28800 | GYR_AM_Sn_9600 | 0,83 | 0,821 | 0,009 |
| QLSNP_178 | DENS_rd_19200 | ELEV_19200 | TEMP_300 | AI_28800 | PLAND_Gr_14400 | 0,834 | 0,821 | 0,013 |
| QLSNP_179 | DENS_rd_19200 | ELEV_19200 | TEMP_300 | AI_28800 | GYR_AM_Gr_4800 | 0,829 | 0,818 | 0,011 |
| QLSNP_180 | DENS_riv_4800 | ELEV_19200 | TEMP_300 | PD_28800 | GYR_AM_Gr_4800 | 0,781 | 0,817 | -0,036 |
| QLSNP_181 | DENS_riv_4800 | ELEV_19200 | TEMP_300 | PD_28800 | PLAND_Gr_14400 | 0,784 | 0,815 | -0,031 |
| QLSNP_182 | DENS_set_28800 | ELEV_19200 | TEMP_300 | PD_28800 | AREA_AM_Shr_19200 | 0,782 | 0,812 | -0,030 |
| QLSNP_183 | DENS_rd_19200 | ELEV_19200 | TEMP_300 | AI_28800 | GYR_AM_Sn_9600 | 0,838 | 0,811 | 0,027 |
| QLSNP_184 | DENS_set_28800 | ELEV_19200 | TEMP_300 | AI_28800 | AREA_AM_Shr_19200 | 0,796 | 0,808 | -0,012 |
| QLSNP_185 | DENS_rd_19200 | ELEV_19200 | TEMP_300 | PD_28800 | GYR_AM_Gr_4800 | 0,802 | 0,806 | -0,004 |
| QLSNP_186 | DENS_rd_19200 | ELEV_19200 | TEMP_300 | PD_28800 | PLAND_Gr_14400 | 0,802 | 0,805 | -0,003 |
| QLSNP_187 | DENS_riv_4800 | ELEV_19200 | TEMP_300 | PD_28800 | AREA_AM_Shr_19200 | 0,777 | 0,802 | -0,025 |
| QLSNP_188 | DENS_riv_4800 | ELEV_19200 | TEMP_300 | PD_28800 | GYR_AM_Sn_9600 | 0,802 | 0,802 | 0,000 |
| QLSNP_189 | DENS_riv_4800 | ELEV_19200 | TEMP_300 | AI_28800 | AREA_AM_Shr_19200 | 0,797 | 0,8 | -0,003 |
| QLSNP_190 | DENS_rd_19200 | ELEV_19200 | TEMP_300 | PD_28800 | AREA_AM_Br_9600 | 0,81 | 0,799 | 0,011 |
| QLSNP_191 | DENS_rd_19200 | ELEV_19200 | TEMP_300 | AI_28800 | AREA_AM_Br_9600 | 0,826 | 0,798 | 0,028 |
| QLSNP_192 | DENS_rd_19200 | ELEV_19200 | TEMP_300 | AI_28800 | AREA_AM_Shr_19200 | 0,815 | 0,797 | 0,018 |
| QLSNP_193 | DENS_rd_19200 | ELEV_19200 | TEMP_300 | PD_28800 | GYR_AM_Sn_9600 | 0,819 | 0,797 | 0,022 |
| QLSNP_194 | DENS_set_28800 | ELEV_19200 | TEMP_300 | PD_28800 | AREA_AM_Br_9600 | 0,784 | 0,793 | -0,009 |
| QLSNP_195 | DENS_riv_4800 | ELEV_19200 | TEMP_300 | PD_28800 | AREA_AM_Br_9600 | 0,786 | 0,792 | -0,006 |
| QLSNP_196 | DENS_rd_19200 | ELEV_19200 | TEMP_300 | PD_28800 | PLAND_Br_14400 | 0,816 | 0,79 | 0,026 |
| QLSNP_197 | DENS_set_28800 | ELEV_19200 | TEMP_300 | AI_28800 | AREA_AM_Br_9600 | 0,801 | 0,789 | 0,012 |
| QLSNP_198 | DENS_riv_4800 | ELEV_19200 | TEMP_300 | AI_28800 | AREA_AM_Br_9600 | 0,806 | 0,789 | 0,017 |
| QLSNP_199 | DENS_rd_19200 | ELEV_19200 | TEMP_300 | PD_28800 | AREA_AM_Shr_19200 | 0,797 | 0,788 | 0,009 |
| QLSNP_200 | DENS_rd_19200 | ELEV_19200 | TEMP_300 | AI_28800 | PLAND_Br_14400 | 0,817 | 0,788 | 0,029 |
| QLSNP_201 | DENS_rd_19200 | ELEV_19200 | TEMP_300 | AI_28800 | GYR_AM_NLF_28800 | 0,826 | 0,785 | 0,041 |
| QLSNP_202 | DENS_riv_4800 | ELEV_19200 | TEMP_300 | PD_28800 | PLAND_Br_14400 | 0,796 | 0,783 | 0,013 |
| QLSNP_203 | DENS_set_28800 | ELEV_19200 | TEMP_300 | PD_28800 | PLAND_Br_14400 | 0,795 | 0,782 | 0,013 |
| QLSNP_204 | DENS_riv_4800 | ELEV_19200 | TEMP_300 | AI_28800 | PLAND_Br_14400 | 0,797 | 0,779 | 0,018 |
| QLSNP_205 | DENS_set_28800 | ELEV_19200 | TEMP_300 | AI_28800 | PLAND_Br_14400 | 0,794 | 0,778 | 0,016 |
| QLSNP_206 | DENS_set_28800 | ELEV_19200 | TEMP_300 | AI_28800 | GYR_AM_NLF_28800 | 0,804 | 0,778 | 0,026 |
| QLSNP_207 | DENS_rd_19200 | ELEV_19200 | TEMP_300 | PD_28800 | GYR_AM_NLF_28800 | 0,812 | 0,777 | 0,035 |
| QLSNP_208 | DENS_riv_4800 | ELEV_19200 | TEMP_300 | AI_28800 | GYR_AM_NLF_28800 | 0,805 | 0,776 | 0,029 |
| QLSNP_209 | DENS_set_28800 | ELEV_19200 | TEMP_300 | PD_28800 | GYR_AM_NLF_28800 | 0,792 | 0,775 | 0,017 |
| QLSNP_210 | DENS_riv_4800 | ELEV_19200 | TEMP_300 | PD_28800 | GYR_AM_NLF_28800 | 0,79 | 0,769 | 0,021 |

Supplementary Table S3. List of 210 multivariate models in Qilian Shan National Park, ranked by decreasing values of AUC (first ranking order). Models showing equal AUC values have been then ranked following increasing AUC_diff_ values (second ranking order).

| **QMLNR** |  |  |  |  |  |  |  |  |
| --- | --- | --- | --- | --- | --- | --- | --- | --- |
| **Model number** | **Linear/point features** | **Topographic** | **Climatic** | **Landcover Landscape-level** | **Landcover Class-level** | **AUC train** | **AUC test** | **AUC_diff_** |
| **QMLNR_1** | **Dens_riv_19200** | **CTI_14400** | **TEMP_300** | **CWED_28800** | **PLAND_Gr_14400** | **0,977** | **0,975** | **0,002** |
| **QMLNR_2** | **Dens_riv_19200** | **CTI_14400** | **TEMP_300** | **CWED_28800** | **GYR_AM_ Gr_19200** | **0,972** | **0,973** | **-0,001** |
| **QMLNR_3** | **Dens_riv_19200** | **SLP_28800** | **TEMP_300** | **CWED_28800** | **PLAND_Br_28800** | **0,968** | **0,972** | **-0,004** |
| **QMLNR_4** | **Dens_riv_19200** | **CTI_14400** | **TEMP_300** | **CWED_28800** | **GYR_AM_Br_28800** | **0,972** | **0,972** | **0,000** |
| **QMLNR_5** | **Dens_riv_19200** | **CTI_14400** | **TEMP_300** | **CWED_28800** | **GYR_AM_NLF_14400** | **0,973** | **0,972** | **0,001** |
| **QMLNR_6** | **Dens_riv_19200** | **CTI_14400** | **TEMP_300** | **PD_19200** | **PLAND_Gr_14400** | **0,974** | **0,972** | **0,002** |
| **QMLNR_7** | **Dens_set_9600** | **CTI_14400** | **TEMP_300** | **CWED_28800** | **PLAND_Gr_14400** | **0,975** | **0,972** | **0,003** |
| **QMLNR_8** | **Dens_riv_19200** | **SLP_28800** | **TEMP_300** | **CWED_28800** | **GYR_AM_Br_28800** | **0,963** | **0,971** | **-0,008** |
| **QMLNR_9** | **Dens_set_9600** | **CTI_14400** | **TEMP_300** | **CWED_28800** | **GYR_AM_ Gr_19200** | **0,97** | **0,971** | **-0,001** |
| **QMLNR_10** | **Dens_set_9600** | **CTI_14400** | **TEMP_300** | **CWED_28800** | **GYR_AM_Br_28800** | **0,971** | **0,971** | **0,000** |
| QMLNR_11 | Dens_riv_19200 | CTI_14400 | TEMP_300 | CWED_28800 | PLAND_Br_28800 | 0,972 | 0,971 | 0,001 |
| QMLNR_12 | Dens_riv_19200 | SLP_28800 | TEMP_300 | PD_19200 | AREA_AM_Shr_28800 | 0,963 | 0,97 | -0,007 |
| QMLNR_13 | Dens_riv_19200 | SLP_28800 | TEMP_300 | PD_19200 | PLAND_Br_28800 | 0,965 | 0,97 | -0,005 |
| QMLNR_14 | Dens_riv_19200 | CTI_14400 | TEMP_300 | PD_19200 | GYR_AM_Br_28800 | 0,969 | 0,97 | -0,001 |
| QMLNR_15 | Dens_set_9600 | SLP_28800 | TEMP_300 | CWED_28800 | GYR_AM_Br_28800 | 0,961 | 0,969 | -0,008 |
| QMLNR_16 | Dens_riv_19200 | SLP_28800 | TEMP_300 | CWED_28800 | GYR_AM_NLF_14400 | 0,966 | 0,969 | -0,003 |
| QMLNR_17 | Dens_set_9600 | CTI_14400 | TEMP_300 | PD_19200 | GYR_AM_Br_28800 | 0,968 | 0,969 | -0,001 |
| QMLNR_18 | Dens_riv_19200 | CTI_14400 | TEMP_300 | PD_19200 | PLAND_Br_28800 | 0,968 | 0,969 | -0,001 |
| QMLNR_19 | Dens_set_9600 | CTI_14400 | TEMP_300 | CWED_28800 | PLAND_Br_28800 | 0,971 | 0,969 | 0,002 |
| QMLNR_20 | Dens_riv_19200 | CTI_14400 | TEMP_300 | PD_19200 | GYR_AM_NLF_14400 | 0,971 | 0,969 | 0,002 |
| QMLNR_21 | Dens_riv_19200 | CTI_14400 | TEMP_300 | CWED_28800 | AREA_AM_Shr_28800 | 0,971 | 0,969 | 0,002 |
| QMLNR_22 | Dens_riv_19200 | CTI_14400 | TEMP_300 | CWED_28800 | AREA_AM_Sn_28800 | 0,974 | 0,969 | 0,005 |
| QMLNR_23 | Dens_set_9600 | SLP_28800 | TEMP_300 | CWED_28800 | PLAND_Br_28800 | 0,963 | 0,968 | -0,005 |
| QMLNR_24 | Dens_riv_19200 | SLP_28800 | TEMP_300 | PD_19200 | GYR_AM_NLF_14400 | 0,965 | 0,968 | -0,003 |
| QMLNR_25 | Dens_riv_19200 | SLP_28800 | TEMP_300 | CWED_28800 | AREA_AM_Sn_28800 | 0,968 | 0,968 | 0,000 |
| QMLNR_26 | Dens_riv_19200 | CTI_14400 | TEMP_300 | PD_19200 | AREA_AM_Shr_28800 | 0,969 | 0,968 | 0,001 |
| QMLNR_27 | Dens_set_9600 | CTI_14400 | TEMP_300 | PD_19200 | PLAND_Gr_14400 | 0,97 | 0,968 | 0,002 |
| QMLNR_28 | Dens_set_9600 | CTI_14400 | TEMP_300 | CWED_28800 | GYR_AM_NLF_14400 | 0,971 | 0,968 | 0,003 |
| QMLNR_29 | Dens_riv_19200 | CTI_14400 | TEMP_300 | AI_19200 | PLAND_Gr_14400 | 0,971 | 0,968 | 0,003 |
| QMLNR_30 | Dens_riv_19200 | SLP_28800 | TEMP_300 | PD_19200 | GYR_AM_Br_28800 | 0,961 | 0,967 | -0,006 |
| QMLNR_31 | Dens_riv_19200 | SLP_28800 | TEMP_300 | AI_19200 | PLAND_Br_28800 | 0,964 | 0,967 | -0,003 |
| QMLNR_32 | Dens_rd_28800 | CTI_14400 | TEMP_300 | CWED_28800 | GYR_AM_ Gr_19200 | 0,974 | 0,967 | 0,007 |
| QMLNR_33 | Dens_rd_28800 | CTI_14400 | TEMP_300 | CWED_28800 | PLAND_Gr_14400 | 0,975 | 0,967 | 0,008 |
| QMLNR_34 | Dens_set_9600 | SLP_28800 | TEMP_300 | PD_19200 | PLAND_Br_28800 | 0,962 | 0,966 | -0,004 |
| QMLNR_35 | Dens_riv_19200 | SLP_28800 | TEMP_300 | CWED_28800 | PLAND_Gr_14400 | 0,963 | 0,966 | -0,003 |
| QMLNR_36 | Dens_riv_19200 | SLP_28800 | TEMP_300 | CWED_28800 | GYR_AM_ Gr_19200 | 0,963 | 0,966 | -0,003 |
| QMLNR_37 | Dens_riv_19200 | CTI_14400 | TEMP_300 | AI_19200 | GYR_AM_Br_28800 | 0,964 | 0,966 | -0,002 |
| QMLNR_38 | Dens_set_9600 | CTI_14400 | TEMP_300 | PD_19200 | PLAND_Br_28800 | 0,966 | 0,966 | 0,000 |
| QMLNR_39 | Dens_set_9600 | CTI_14400 | TEMP_300 | CWED_28800 | AREA_AM_Shr_28800 | 0,969 | 0,966 | 0,003 |
| QMLNR_40 | Dens_riv_19200 | CTI_14400 | TEMP_300 | PD_19200 | GYR_AM_ Gr_19200 | 0,969 | 0,966 | 0,003 |
| QMLNR_41 | Dens_riv_19200 | CTI_14400 | TEMP_300 | PD_19200 | AREA_AM_Sn_28800 | 0,97 | 0,966 | 0,004 |
| QMLNR_42 | Dens_set_9600 | CTI_14400 | TEMP_300 | CWED_28800 | AREA_AM_Sn_28800 | 0,972 | 0,966 | 0,006 |
| QMLNR_43 | Dens_set_9600 | SLP_28800 | TEMP_300 | PD_19200 | GYR_AM_Br_28800 | 0,958 | 0,965 | -0,007 |
| QMLNR_44 | Dens_riv_19200 | SLP_28800 | TEMP_300 | CWED_28800 | AREA_AM_Shr_28800 | 0,962 | 0,965 | -0,003 |
| QMLNR_45 | Dens_riv_19200 | SLP_28800 | TEMP_300 | PD_19200 | PLAND_Gr_14400 | 0,963 | 0,965 | -0,002 |
| QMLNR_46 | Dens_riv_19200 | CTI_14400 | TEMP_300 | AI_19200 | PLAND_Br_28800 | 0,966 | 0,965 | 0,001 |
| QMLNR_47 | Dens_rd_28800 | CTI_14400 | TEMP_300 | CWED_28800 | GYR_AM_NLF_14400 | 0,973 | 0,965 | 0,008 |
| QMLNR_48 | Dens_rd_28800 | CTI_14400 | TEMP_300 | CWED_28800 | GYR_AM_Br_28800 | 0,973 | 0,965 | 0,008 |
| QMLNR_49 | Dens_rd_28800 | CTI_14400 | TEMP_300 | PD_19200 | GYR_AM_Br_28800 | 0,971 | 0,964 | 0,007 |
| QMLNR_50 | Dens_rd_28800 | CTI_14400 | TEMP_300 | CWED_28800 | PLAND_Br_28800 | 0,971 | 0,964 | 0,007 |
| QMLNR_51 | Dens_riv_19200 | SLP_28800 | TEMP_300 | AI_19200 | GYR_AM_Br_28800 | 0,957 | 0,963 | -0,006 |
| QMLNR_52 | Dens_set_9600 | CTI_14400 | TEMP_300 | AI_19200 | GYR_AM_Br_28800 | 0,962 | 0,963 | -0,001 |
| QMLNR_53 | Dens_set_9600 | CTI_14400 | TEMP_300 | PD_19200 | GYR_AM_ Gr_19200 | 0,965 | 0,963 | 0,002 |
| QMLNR_54 | Dens_set_9600 | CTI_14400 | TEMP_300 | PD_19200 | GYR_AM_NLF_14400 | 0,966 | 0,963 | 0,003 |
| QMLNR_55 | Dens_set_9600 | CTI_14400 | TEMP_300 | AI_19200 | PLAND_Gr_14400 | 0,966 | 0,963 | 0,003 |
| QMLNR_56 | Dens_riv_19200 | CTI_14400 | TEMP_300 | AI_19200 | GYR_AM_NLF_14400 | 0,966 | 0,963 | 0,003 |
| QMLNR_57 | Dens_rd_28800 | CTI_14400 | TEMP_300 | PD_19200 | PLAND_Br_28800 | 0,969 | 0,963 | 0,006 |
| QMLNR_58 | Dens_rd_28800 | CTI_14400 | TEMP_300 | PD_19200 | PLAND_Gr_14400 | 0,972 | 0,963 | 0,009 |
| QMLNR_59 | Dens_rd_28800 | CTI_14400 | TEMP_300 | CWED_28800 | AREA_AM_Sn_28800 | 0,973 | 0,963 | 0,010 |
| QMLNR_60 | Dens_set_9600 | SLP_28800 | TEMP_300 | AI_19200 | PLAND_Br_28800 | 0,959 | 0,962 | -0,003 |
| QMLNR_61 | Dens_set_9600 | CTI_14400 | TEMP_300 | AI_19200 | PLAND_Br_28800 | 0,964 | 0,962 | 0,002 |
| QMLNR_62 | Dens_rd_28800 | CTI_14400 | TEMP_300 | PD_19200 | GYR_AM_NLF_14400 | 0,971 | 0,962 | 0,009 |
| QMLNR_63 | Dens_riv_19200 | SLP_28800 | TEMP_300 | PD_19200 | AREA_AM_Sn_28800 | 0,965 | 0,961 | 0,004 |
| QMLNR_64 | Dens_rd_28800 | CTI_14400 | TEMP_300 | AI_19200 | GYR_AM_Br_28800 | 0,968 | 0,961 | 0,007 |
| QMLNR_65 | Dens_rd_28800 | CTI_14400 | TEMP_300 | CWED_28800 | AREA_AM_Shr_28800 | 0,971 | 0,961 | 0,010 |
| QMLNR_66 | Dens_set_9600 | SLP_28800 | TEMP_300 | AI_19200 | GYR_AM_Br_28800 | 0,953 | 0,96 | -0,007 |
| QMLNR_67 | Dens_set_9600 | SLP_28800 | TEMP_300 | CWED_28800 | GYR_AM_ Gr_19200 | 0,958 | 0,96 | -0,002 |
| QMLNR_68 | Dens_riv_19200 | SLP_28800 | TEMP_300 | AI_19200 | GYR_AM_NLF_14400 | 0,959 | 0,96 | -0,001 |
| QMLNR_69 | Dens_riv_19200 | SLP_28800 | TEMP_300 | PD_19200 | GYR_AM_ Gr_19200 | 0,96 | 0,96 | 0,000 |
| QMLNR_70 | Dens_set_9600 | CTI_14400 | TEMP_300 | PD_19200 | AREA_AM_Shr_28800 | 0,962 | 0,96 | 0,002 |
| QMLNR_71 | Dens_rd_28800 | SLP_28800 | TEMP_300 | PD_19200 | PLAND_Br_28800 | 0,965 | 0,96 | 0,005 |
| QMLNR_72 | Dens_rd_28800 | SLP_28800 | TEMP_300 | CWED_28800 | AREA_AM_Sn_28800 | 0,965 | 0,96 | 0,005 |
| QMLNR_73 | Dens_rd_28800 | SLP_28800 | TEMP_300 | CWED_28800 | PLAND_Br_28800 | 0,966 | 0,96 | 0,006 |
| QMLNR_74 | Dens_set_9600 | CTI_14400 | TEMP_300 | PD_19200 | AREA_AM_Sn_28800 | 0,966 | 0,96 | 0,006 |
| QMLNR_75 | Dens_rd_28800 | CTI_14400 | TEMP_300 | AI_19200 | PLAND_Br_28800 | 0,967 | 0,96 | 0,007 |
| QMLNR_76 | Dens_rd_28800 | CTI_14400 | TEMP_300 | AI_19200 | PLAND_Gr_14400 | 0,972 | 0,96 | 0,012 |
| QMLNR_77 | Dens_set_9600 | SLP_28800 | TEMP_300 | CWED_28800 | AREA_AM_Sn_28800 | 0,962 | 0,959 | 0,003 |
| QMLNR_78 | Dens_riv_19200 | CTI_14400 | TEMP_300 | AI_19200 | GYR_AM_ Gr_19200 | 0,964 | 0,959 | 0,005 |
| QMLNR_79 | Dens_rd_28800 | SLP_28800 | TEMP_300 | CWED_28800 | GYR_AM_Br_28800 | 0,966 | 0,959 | 0,007 |
| QMLNR_80 | Dens_rd_28800 | CTI_14400 | TEMP_300 | PD_19200 | AREA_AM_Shr_28800 | 0,968 | 0,959 | 0,009 |
| QMLNR_81 | Dens_rd_28800 | CTI_14400 | TEMP_300 | PD_19200 | GYR_AM_ Gr_19200 | 0,971 | 0,959 | 0,012 |
| QMLNR_82 | Dens_set_9600 | SLP_28800 | TEMP_300 | CWED_28800 | GYR_AM_NLF_14400 | 0,956 | 0,958 | -0,002 |
| QMLNR_83 | Dens_set_9600 | CTI_14400 | TEMP_300 | AI_19200 | GYR_AM_ Gr_19200 | 0,961 | 0,958 | 0,003 |
| QMLNR_84 | Dens_rd_28800 | SLP_28800 | TEMP_300 | PD_19200 | GYR_AM_Br_28800 | 0,966 | 0,958 | 0,008 |
| QMLNR_85 | Dens_rd_28800 | CTI_14400 | TEMP_300 | PD_19200 | AREA_AM_Sn_28800 | 0,97 | 0,958 | 0,012 |
| QMLNR_86 | Dens_riv_19200 | SLP_28800 | TEMP_300 | AI_19200 | AREA_AM_Sn_28800 | 0,961 | 0,957 | 0,004 |
| QMLNR_87 | Dens_rd_28800 | SLP_28800 | TEMP_300 | CWED_28800 | PLAND_Gr_14400 | 0,965 | 0,957 | 0,008 |
| QMLNR_88 | Dens_rd_28800 | SLP_28800 | TEMP_300 | CWED_28800 | GYR_AM_ Gr_19200 | 0,966 | 0,957 | 0,009 |
| QMLNR_89 | Dens_rd_28800 | CTI_14400 | TEMP_300 | AI_19200 | GYR_AM_NLF_14400 | 0,967 | 0,957 | 0,010 |
| QMLNR_90 | Dens_set_9600 | SLP_28800 | TEMP_300 | CWED_28800 | PLAND_Gr_14400 | 0,952 | 0,956 | -0,004 |
| QMLNR_91 | Dens_set_9600 | CTI_14400 | TEMP_300 | AI_19200 | GYR_AM_NLF_14400 | 0,961 | 0,956 | 0,005 |
| QMLNR_92 | Dens_rd_28800 | SLP_28800 | TEMP_300 | AI_19200 | PLAND_Br_28800 | 0,963 | 0,956 | 0,007 |
| QMLNR_93 | Dens_riv_19200 | SLP_28800 | TEMP_300 | AI_19200 | GYR_AM_ Gr_19200 | 0,956 | 0,955 | 0,001 |
| QMLNR_94 | Dens_riv_19200 | SLP_28800 | TEMP_300 | AI_19200 | AREA_AM_Shr_28800 | 0,948 | 0,954 | -0,006 |
| QMLNR_95 | Dens_riv_19200 | SLP_28800 | TEMP_300 | AI_19200 | PLAND_Gr_14400 | 0,954 | 0,954 | 0,000 |
| QMLNR_96 | Dens_rd_28800 | SLP_28800 | TEMP_300 | AI_19200 | GYR_AM_Br_28800 | 0,962 | 0,954 | 0,008 |
| QMLNR_97 | Dens_riv_19200 | CTI_14400 | TEMP_300 | AI_19200 | AREA_AM_Sn_28800 | 0,963 | 0,953 | 0,010 |
| QMLNR_98 | Dens_set_9600 | SLP_28800 | TEMP_300 | CWED_28800 | AREA_AM_Shr_28800 | 0,946 | 0,952 | -0,006 |
| QMLNR_99 | Dens_set_9600 | SLP_28800 | TEMP_300 | PD_19200 | GYR_AM_NLF_14400 | 0,95 | 0,952 | -0,002 |
| QMLNR_100 | Dens_riv_19200 | CTI_14400 | TEMP_300 | AI_19200 | AREA_AM_Shr_28800 | 0,953 | 0,952 | 0,001 |
| QMLNR_101 | Dens_rd_28800 | CTI_14400 | TEMP_300 | AI_19200 | GYR_AM_ Gr_19200 | 0,966 | 0,952 | 0,014 |
| QMLNR_102 | Dens_set_9600 | SLP_28800 | TEMP_300 | PD_19200 | AREA_AM_Shr_28800 | 0,942 | 0,951 | -0,009 |
| QMLNR_103 | Dens_rd_28800 | SLP_28800 | TEMP_300 | CWED_28800 | GYR_AM_NLF_14400 | 0,961 | 0,95 | 0,011 |
| QMLNR_104 | Dens_set_9600 | CTI_14400 | TEMP_300 | AI_19200 | AREA_AM_Shr_28800 | 0,947 | 0,948 | -0,001 |
| QMLNR_105 | Dens_rd_28800 | SLP_28800 | TEMP_300 | PD_19200 | GYR_AM_NLF_14400 | 0,957 | 0,948 | 0,009 |
| QMLNR_106 | Dens_rd_28800 | CTI_14400 | TEMP_300 | AI_19200 | AREA_AM_Shr_28800 | 0,96 | 0,948 | 0,012 |
| QMLNR_107 | Dens_set_9600 | SLP_28800 | TEMP_300 | PD_19200 | PLAND_Gr_14400 | 0,943 | 0,947 | -0,004 |
| QMLNR_108 | Dens_rd_28800 | SLP_28800 | TEMP_300 | PD_19200 | PLAND_Gr_14400 | 0,96 | 0,946 | 0,014 |
| QMLNR_109 | Dens_rd_28800 | CTI_14400 | TEMP_300 | AI_19200 | AREA_AM_Sn_28800 | 0,964 | 0,946 | 0,018 |
| QMLNR_110 | Dens_rd_28800 | SLP_28800 | TEMP_300 | CWED_28800 | AREA_AM_Shr_28800 | 0,958 | 0,945 | 0,013 |
| QMLNR_111 | Dens_rd_28800 | SLP_28800 | TEMP_300 | PD_19200 | AREA_AM_Shr_28800 | 0,954 | 0,943 | 0,011 |
| QMLNR_112 | Dens_set_9600 | CTI_14400 | TEMP_300 | AI_19200 | AREA_AM_Sn_28800 | 0,956 | 0,943 | 0,013 |
| QMLNR_113 | Dens_set_9600 | SLP_28800 | TEMP_300 | AI_19200 | GYR_AM_ Gr_19200 | 0,94 | 0,942 | -0,002 |
| QMLNR_114 | Dens_set_9600 | SLP_28800 | TEMP_300 | AI_19200 | GYR_AM_NLF_14400 | 0,941 | 0,941 | 0,000 |
| QMLNR_115 | Dens_set_9600 | SLP_28800 | TEMP_300 | PD_19200 | GYR_AM_ Gr_19200 | 0,944 | 0,941 | 0,003 |
| QMLNR_116 | Dens_set_9600 | SLP_28800 | TEMP_300 | PD_19200 | AREA_AM_Sn_28800 | 0,952 | 0,941 | 0,011 |
| QMLNR_117 | Dens_rd_28800 | SLP_28800 | TEMP_300 | PD_19200 | GYR_AM_ Gr_19200 | 0,959 | 0,941 | 0,018 |
| QMLNR_118 | Dens_rd_28800 | SLP_28800 | TEMP_300 | AI_19200 | GYR_AM_NLF_14400 | 0,95 | 0,938 | 0,012 |
| QMLNR_119 | Dens_rd_28800 | SLP_28800 | TEMP_300 | PD_19200 | AREA_AM_Sn_28800 | 0,958 | 0,938 | 0,020 |
| QMLNR_120 | Dens_rd_28800 | SLP_28800 | TEMP_300 | AI_19200 | GYR_AM_ Gr_19200 | 0,953 | 0,935 | 0,018 |
| QMLNR_121 | Dens_set_9600 | SLP_28800 | TEMP_300 | AI_19200 | PLAND_Gr_14400 | 0,931 | 0,933 | -0,002 |
| QMLNR_122 | Dens_rd_28800 | SLP_28800 | TEMP_300 | AI_19200 | PLAND_Gr_14400 | 0,951 | 0,933 | 0,018 |
| QMLNR_123 | Dens_set_9600 | SLP_28800 | TEMP_300 | AI_19200 | AREA_AM_Sn_28800 | 0,943 | 0,932 | 0,011 |
| QMLNR_124 | Dens_set_9600 | SLP_28800 | TEMP_300 | AI_19200 | AREA_AM_Shr_28800 | 0,915 | 0,926 | -0,011 |
| QMLNR_125 | Dens_rd_28800 | SLP_28800 | TEMP_300 | AI_19200 | AREA_AM_Shr_28800 | 0,937 | 0,923 | 0,014 |
| QMLNR_126 | Dens_rd_28800 | SLP_28800 | TEMP_300 | AI_19200 | AREA_AM_Sn_28800 | 0,947 | 0,922 | 0,025 |

Supplementary Table S4. List of 126 multivariate models in Qomolangma National Nature Reserve, ranked by decreasing values of AUC (first ranking order). Models showing equal AUC values have been then ranked following increasing AUC_diff_ values (second ranking order).


|  |  | **Best scale RAW** | | **SR_1200** | | **SR_2400** | | **SR_4800** | | **SR_9600** | | **GK_1200** | | **GK_2400** | | **GK_4800** | | **GK_9600** | |
| --- | --- | --- | --- | --- | --- | --- | --- | --- | --- | --- | --- | --- | --- | --- | --- | --- | --- | --- | --- |
| *Model* | *Variables* | *Percent* | *Permutation* | *Percent* | *Permutation* | *Percent* | *Permutation* | *Percent* | *Permutation* | *Percent* | *Permutation* | *Percent* | *Permutation* | *Percent* | *Permutation* | *Percent* | *Permutation* | *Percent* | *Permutation* |
| **QLSNP_1** | DENS_set_28800 | 8 | 5,3 | 6,6 | 4,8 | 6,3 | 1,9 | 4,7 | 3,1 | 3 | 3 | 7,6 | 4,6 | 7,2 | 4,2 | 6 | 3,3 | 4,5 | 2,4 |
|  | SLP_2400 | 51,1 | 33,7 | 52,5 | 2935 | 54,7 | 32,4 | 61,9 | 44,7 | 64,4 | 51,6 | 52 | 33,4 | 53,3 | 33,9 | 57,6 | 35,8 | 63,3 | 40,1 |
|  | TEMP_300 | 28,1 | 46,4 | 32 | 48,7 | 31 | 51,8 | 29,9 | 44,7 | 28,7 | 51,6 | 29 | 47,9 | 28,4 | 48,6 | 28,3 | 49,6 | 27,6 | 51,2 |
|  | AI_28800 | 5,8 | 6,7 | 4,4 | 10,4 | 3,5 | 9,5 | 0,1 | 0,4 | 0,1 | 0,1 | 5,5 | 6,9 | 5 | 6,6 | 3,4 | 5,8 | 0,9 | 2,3 |
|  | GYR_AM_Gr_4800 | 7 | 7,9 | 4,5 | 6,6 | 4,4 | 4,4 | 3,5 | 1,2 | 3,9 | 0,7 | 4,9 | 7,3 | 6,1 | 6,7 | 4,7 | 5,5 | 3,7 | 4 |
| **QLSNP_2** | DENS_set_28800 | 7,1 | 5,3 | 6,2 | 4,9 | 6,3 | 2 | 4,8 | 3,7 | 3,3 | 3,7 | 7,1 | 4,6 | 6,6 | 4,3 | 5,7 | 3,5 | 4,3 | 2,9 |
|  | SLP_2400 | 53,7 | 31,7 | 53,3 | 27,9 | 55,6 | 32,8 | 62,3 | 45,7 | 65 | 51,1 | 53,9 | 31,1 | 54,8 | 31,9 | 58,3 | 34,4 | 63,4 | 39,8 |
|  | TEMP_300 | 31,6 | 53,6 | 35,1 | 52,7 | 33,4 | 54,7 | 31,2 | 50,3 | 30,5 | 38,3 | 32,4 | 54,5 | 32,1 | 54,4 | 31,5 | 54,1 | 30,6 | 54,4 |
|  | AI_28800 | 5,4 | 8,8 | 4 | 11,5 | 3,5 | 9,4 | 0,4 | 0,1 | 0,7 | 1,6 | 5 | 9 | 4,9 | 8,7 | 3,2 | 7,6 | 0,7 | 2,9 |
|  | PLAND_Gr_14400 | 2,2 | 0,6 | 1,3 | 3,1 | 1,1 | 1 | 1,4 | 0,2 | 0,6 | 5,2 | 1,6 | 0,8 | 1,7 | 0,7 | 1,2 | 0,4 | 1 | 0 |
| **QLSNP_4** | DENS_riv_4800 | 1,5 | 1 | 1,8 | 2 | 1,5 | 0,7 | 0,9 | 2,3 | 0,5 | 0,6 | 1,7 | 1 | 1,5 | 1 | 1,5 | 0,8 | 0,8 | 5 |
|  | SLP_2400 | 55,2 | 32,5 | 55,1 | 28,6 | 57,7 | 32,7 | 63,9 | 46,8 | 65,8 | 53,6 | 54,9 | 32,3 | 55,9 | 33 | 59,1 | 35,1 | 64,2 | 40,2 |
|  | TEMP_300 | 34,4 | 54,6 | 37,2 | 52,7 | 36,1 | 54,2 | 34 | 50,9 | 32,6 | 40,4 | 35,8 | 55,4 | 35,9 | 55,2 | 34,8 | 54,6 | 33,7 | 55 |
|  | AI_28800 | 6,6 | 10,6 | 4,7 | 12,5 | 3,8 | 10,3 | 0,2 | 0 | 0,3 | 1,1 | 5,9 | 10,1 | 5,3 | 9,7 | 3,6 | 8,6 | 0,6 | 4,1 |
|  | PLAND_Gr_14400 | 2,2 | 1,3 | 1,1 | 4,2 | 0,8 | 2,1 | 1,2 | 0 | 0,8 | 4,3 | 1,8 | 1,3 | 1,4 | 1,1 | 1,1 | 0,9 | 0,7 | 0,1 |
| **QLSNP_5** | DENS_rd_19200 | 9,6 | 8,8 | 7,5 | 8,8 | 6 | 6,4 | 4,3 | 3,7 | 5,9 | 9 | 9,4 | 8,5 | 9,3 | 8,2 | 7,5 | 7,2 | 5,6 | 6,7 |
|  | SLP_2400 | 50,2 | 27,7 | 51,6 | 25,2 | 55,3 | 29,7 | 61,3 | 45,1 | 62 | 46,6 | 50,3 | 27,8 | 51,4 | 28,7 | 55,6 | 31,3 | 61 | 36,1 |
|  | TEMP_300 | 31,9 | 51,5 | 36 | 49,8 | 33,9 | 51,6 | 33 | 50,9 | 31,3 | 39 | 33,4 | 52,6 | 33,6 | 52,8 | 33 | 53,1 | 32,1 | 54,1 |
|  | AI_28800 | 5,9 | 8,8 | 3,8 | 11 | 3,7 | 9,2 | 0,2 | 0 | 0,2 | 0,9 | 4,9 | 8,4 | 4,1 | 8 | 2,5 | 6,9 | 0,4 | 2,7 |
|  | PLAND_Gr_14400 | 2,4 | 3,1 | 1,1 | 5,2 | 1,1 | 3,1 | 1,2 | 0,3 | 0,5 | 4,5 | 2 | 2,7 | 1,7 | 2,3 | 1,4 | 1,4 | 0,9 | 0,3 |
| **QLSNP_6** | DENS_rd_19200 | 10,1 | 8 | 7,3 | 8,9 | 6 | 6,2 | 4,2 | 3,9 | 5,4 | 10,3 | 10 | 7,7 | 9,4 | 7,3 | 7,7 | 6,5 | 5,5 | 6 |
|  | SLP_2400 | 49,6 | 31,4 | 50,4 | 27,7 | 53,7 | 30,3 | 59,8 | 43,5 | 61,4 | 45,6 | 50 | 31,2 | 51,9 | 31,6 | 55,7 | 33,5 | 60,8 | 37,1 |
|  | TEMP_300 | 29,2 | 46,9 | 34,6 | 48 | 33,4 | 50,1 | 32,2 | 50,2 | 29,8 | 43 | 30,2 | 48,3 | 30 | 48,8 | 30,2 | 49,9 | 30,1 | 52 |
|  | AI_28800 | 5,1 | 7 | 3,5 | 9,1 | 2,8 | 8,8 | 0 | 0,6 | 0 | 0,2 | 5,2 | 6,6 | 4,2 | 6,6 | 2,9 | 5,4 | 0,3 | 1,7 |
|  | GYR_AM_Gr_4800 | 5,9 | 6,6 | 4,2 | 6,2 | 4,1 | 4,6 | 3,8 | 1,7 | 3,3 | 0,9 | 4,7 | 6,1 | 4,5 | 5,7 | 3,4 | 4,7 | 3,2 | 3,2 |

Supplementary Table S5. Variables percent contribution and permutation importance in the top five performing models at their best performing scale (Table 6) across different corrections, in Qilianshan National Park. SR (Spatial rarefaction) and GK (Gaussian density kernel) are followed by radius in meters.

|  |  | **Best scale RAW** | | **SR_1200** | | **SR_2400** | | **SR_4800** | | **SR_9600** | | **GK_1200** | | **GK_2400** | | **GK_4800** | | **GK_9600** | |
| --- | --- | --- | --- | --- | --- | --- | --- | --- | --- | --- | --- | --- | --- | --- | --- | --- | --- | --- | --- |
| *Model* | *Variables* | *Percent* | *Permutation* | *Percent* | *Permutation* | *Percent* | *Permutation* | *Percent* | *Permutation* | *Percent* | *Permutation* | *Percent* | *Permutation* | *Percent* | *Permutation* | *Percent* | *Permutation* | *Percent* | *Permutation* |
| **QMLNR_1** | DENS_riv_19200 | 2,8 | 1,8 | 4,3 | 3,1 | 4,6 | 6,8 | 1,7 | 4 | 0,3 | 0,5 | 2,7 | 7,1 | 3 | 4,5 | 2,1 | 2,2 | 1,7 | 3 |
|  | CTI_14400 | 25,3 | 28,1 | 23,5 | 21,6 | 26 | 24,6 | 23,6 | 30,2 | 15,1 | 13,7 | 26,1 | 21,5 | 28,1 | 27,2 | 29,3 | 28,7 | 28,7 | 26,3 |
|  | TEMP_300 | 35,4 | 37,5 | 38,4 | 49 | 42,5 | 42,5 | 53,9 | 47,2 | 66,5 | 74,5 | 35,1 | 39,7 | 36,6 | 42,2 | 41,2 | 41,6 | 49,4 | 47,1 |
|  | CWED_28800 | 12,8 | 19,5 | 13,1 | 15,1 | 10,6 | 13,3 | 7 | 12,5 | 2,3 | 4,5 | 13 | 18 | 11,8 | 11,8 | 10,1 | 17,5 | 7,7 | 15,5 |
|  | PLAND_Gr_14400 | 23,7 | 13,1 | 20,7 | 11,1 | 16,3 | 12,9 | 13,7 | 6,1 | 15,7 | 6,8 | 23,1 | 13,7 | 20,5 | 14,2 | 17,4 | 10 | 12,6 | 8,1 |
| **QMLNR_2** | DENS _riv_19200 | 2,4 | 1,1 | 2,8 | 5,5 | 3,1 | 4,4 | 0,7 | 1,1 | 0,2 | 0,1 | 2,1 | 2 | 1,9 | 2,9 | 1,5 | 2,2 | 0,9 | 3,6 |
|  | CTI_14400 | 18 | 18,6 | 17 | 21,7 | 19,1 | 22,2 | 17,7 | 30,3 | 12,2 | 10,9 | 18,9 | 17,8 | 20,3 | 25,7 | 21,2 | 21,4 | 21,3 | 20,9 |
|  | TEMP_300 | 34,7 | 46,8 | 36,9 | 53,4 | 43,8 | 57,7 | 52,7 | 56,5 | 61,9 | 74,5 | 35,6 | 51,9 | 33,9 | 43,9 | 34,9 | 52,4 | 37,4 | 55,5 |
|  | CWED_28800 | 10,5 | 23,4 | 11,6 | 13,2 | 6,5 | 10,5 | 2,8 | 6,1 | 0,9 | 1,8 | 9,1 | 17,7 | 7,7 | 18,5 | 4,9 | 15,7 | 3,2 | 11,9 |
|  | GYR_AM_ Gr_19200 | 34,4 | 10,1 | 31,6 | 6,3 | 27,4 | 5,2 | 26,2 | 6 | 24,8 | 12,7 | 34,3 | 10,7 | 36,2 | 8,9 | 37,5 | 8,3 | 37,2 | 8,2 |
| **QMLNR_4** | DENS _riv_19200 | 4,4 | 0,5 | 5,3 | 2,3 | 4,2 | 3,2 | 1,7 | 3,3 | 2 | 0,7 | 5,6 | 1 | 6,8 | 0,5 | 3,1 | 0,2 | 1,4 | 1 |
|  | CTI_14400 | 10,3 | 22,7 | 9,8 | 20,7 | 16,2 | 22,6 | 21,6 | 19,9 | 20,8 | 14,7 | 10 | 15,5 | 9,4 | 15,1 | 8,8 | 18,3 | 8,6 | 16,9 |
|  | TEMP_300 | 31 | 37,3 | 34,7 | 40,4 | 41,8 | 35,9 | 53,9 | 51,9 | 66,3 | 69,6 | 29,4 | 33,7 | 28,9 | 39,3 | 35,5 | 43,3 | 42 | 48,8 |
|  | CWED_28800 | 17,1 | 16,8 | 18,1 | 18,4 | 17,5 | 24,4 | 15,6 | 17,3 | 9,3 | 10,1 | 18,2 | 15,5 | 18,2 | 15 | 18,6 | 16,5 | 15,6 | 15,1 |
|  | GYR_AM_Br_28800 | 37,2 | 22,7 | 32,1 | 18,1 | 20,3 | 13,9 | 7,2 | 7,6 | 1,6 | 5 | 36,8 | 27,5 | 36,7 | 30,3 | 34,1 | 21,8 | 32,4 | 18,2 |
| **QMLNR_5** | DENS _riv_19200 | 2,9 | 3,7 | 4,3 | 6,8 | 3,9 | 3,7 | 0,7 | 0,8 | 0,3 | 0,1 | 3,2 | 4,2 | 2,5 | 4,1 | 2 | 3 | 1,5 | 3,5 |
|  | CTI_14400 | 26,9 | 22 | 26,1 | 21,5 | 26,9 | 16,5 | 24,9 | 24,5 | 19,5 | 17 | 27,8 | 26,4 | 28,7 | 17,7 | 29,7 | 24,8 | 28,5 | 23,9 |
|  | TEMP_300 | 40,4 | 40,1 | 42,3 | 42,9 | 46,3 | 40,9 | 54,7 | 56,8 | 63,8 | 67,7 | 40,9 | 36,8 | 41,9 | 50,1 | 43,3 | 46,9 | 47,2 | 52,3 |
|  | CWED_28800 | 8,1 | 20 | 9,1 | 19,7 | 5,4 | 11,4 | 4,5 | 5,1 | 1,9 | 0,8 | 6,7 | 15,2 | 6,2 | 15,9 | 4,7 | 13,5 | 3,4 | 10,3 |
|  | GYR_AM_NLF_14400 | 21,8 | 14,2 | 18,3 | 9,2 | 17,6 | 27,4 | 15,2 | 15,8 | 14,5 | 14,4 | 21,4 | 17,4 | 20,7 | 12,2 | 20,2 | 11,9 | 19,3 | 10 |
| **QMLNR_6** | DENS _riv_19200 | 3,5 | 4,1 | 5,4 | 6,9 | 4,4 | 12,4 | 2 | 3,4 | 0,1 | 0,2 | 4 | 5,5 | 3,3 | 4,4 | 2,3 | 3,3 | 1,4 | 2,9 |
|  | CTI_14400 | 25,6 | 24,7 | 24,1 | 16,2 | 24,5 | 16,6 | 20,9 | 23,3 | 14,2 | 10,4 | 26,6 | 24,6 | 27,1 | 20,7 | 27,8 | 20,6 | 26,5 | 17,7 |
|  | TEMP_300 | 40,7 | 43,9 | 42,8 | 49,3 | 47,2 | 52,5 | 56,2 | 52,2 | 65,7 | 79,4 | 41 | 42,5 | 42,1 | 51,4 | 44,5 | 52 | 50,8 | 58,1 |
|  | PD_19200 | 21,8 | 15,6 | 19,7 | 15,8 | 17,9 | 13,3 | 12,6 | 19,5 | 5,6 | 5,1 | 21,6 | 12,1 | 22 | 13,2 | 20,9 | 16,2 | 18,3 | 15,3 |
|  | PLAND_Gr_14400 | 8,3 | 11,8 | 7,9 | 11,8 | 6 | 5,3 | 8,3 | 1,5 | 14,4 | 4,8 | 6,8 | 15,4 | 5,5 | 10,4 | 4,5 | 7,9 | 3 | 6 |

Supplementary Table S6. Variables percent contribution and permutation importance in the top 5 performing models at their best performing scale (Table 6) across different corrections, in Qomolangma National Nature Reserve. SR (Spatial rarefaction) and GK (Gaussian density kernel) are followed by radius in meters.
